# A weight-sharing Bayesian neural network for consistent feature selection with applications in cancer gene expression data

**DOI:** 10.1186/s12859-026-06397-0

**Published:** 2026-02-27

**Authors:** Akanksha Mishra, Wei Xia, Clint Pazhayidam George

**Affiliations:** 1 School of Mathematics and Computer Science, Indian Institute of Technology Goa, Ponda, Goa 403401 India; 2 School of Interdisciplinary Life Sciences, Indian Institute of Technology Goa, Ponda, Goa 403401 India; 3 Amazon, Seattle, WA USA

**Keywords:** High-dimensional data, Spike-and-slab priors, Variational Bayes, Posterior consistency, Cancer gene expression, Biomarker identification, Predictive modeling

## Abstract

****Background**:**

Advances in sequencing technologies generate extensive genetic information. Datasets such as the Cancer Genome Atlas (TCGA) BRCA facilitate large-scale cancer gene expression analyses, providing insights into the molecular mechanisms driving tumor progression. Effective feature selection—identifying cancer or its subtype-related genes from expression profiles—is critical, as it enhances diagnostic accuracy and guides personalized therapies. However, feature selection is hindered by dimensionality, low sample sizes, and nonlinear interactions within the data, making traditional models, including LASSO and its Bayesian counterparts, inadequate for interpretable feature selection. We thus propose a novel weight-sharing Bayesian neural network (wsBNN) leveraging shared spike-and-slab priors within a neural network framework to enable adaptive weight shrinkage for efficient and interpretable feature selection.

****Results**:**

We incorporate a scalable variational Bayes inference embedded in backpropagation while ensuring effective feature selection. Studying the theoretical properties of the variational posterior shows insights into the performance and theoretical guarantees of wsBNN. Both simulated and real-world dataset experiments show that wsBNN surpasses state-of-the-art nonlinear methods, including frequentist neural networks, in terms of predictive performance and consistency in feature selection. Furthermore, it competes well with classical methods such as Random Forests and Gradient Boosting. TCGA BRCA data study highlights wsBNN’s practical applicability in identifying key biomarkers, particularly in Breast Cancer analysis. The model effectively captured cancer-associated genes and pathways—particularly those related to ERBB2 and PI3K/AKT signaling, immune regulation, and cell cycle control—showing superior biological relevance and interpretability compared to baseline methods.

****Conclusions**:**

Our findings—e.g., consistently identifying relevant biomarkers—position wsBNN as a promising approach for feature selection in high-dimensional genomic datasets and show its potential to advance precision medicine. By integrating weight-grouping with shared spike-and-slab priors within a Bayesian neural network, wsBNN effectively balances sparsity, interpretability, and scalability. wsBNN’s ability to recover biologically relevant genes and pathways highlights its importance for interpretable genomic analysis.

## Background

Gene expression data, such as that from *The Cancer Genome Atlas* [1, TCGA], measures the transcriptional activity of thousands of genes across different biological samples, offering a comprehensive snapshot of cellular functions. The biological samples could be cells, tissues, or organisms, and the activity levels are often quantified using high-throughput techniques such as microarrays or RNA sequencing (RNA-seq) to quantify mRNA abundance. In cancer research, gene expression profiling allows researchers to identify molecular signatures associated with different cancer types, subtypes, and stages, aiding in diagnosis, prognosis, and treatment selection. A key challenge to working with such data is the high dimensionality, i.e., the number of genes (*d*) profiled is often large and often exceeds the number of samples (*n*) available, leading to statistical and computational challenges such as overfitting, poor generalization, and difficulty in extracting biologically meaningful features.

Feature selection aims to identify an optimal subset $s^* \ll d$ from a large feature pool. However, its combinatorial search space of $2^d$ makes exhaustive methods impractical, particularly for large *d* or neural networks [2, 3]. Existing methods fall into three categories: *filter* methods, which remove irrelevant features before model training; *wrapper* methods, which evaluate feature subsets by retraining models; and *embedded* methods, which integrate selection into model learning [e.g. 4].

In high-dimensional, low-sample-size settings, traditional embedded methods, such as penalized Bayesian ordinal response models [5], have been used for gene selection. However, they lack neural network-based flexibility to capture complex nonlinear interactions. These methods are limited to ordinal outcomes and rely on computationally expensive Markov Chain Monte Carlo [6, MCMC] sampling. Moreover, they do not explicitly address overfitting, a challenge mitigated by Bayesian neural networks with spike-and-slab priors through regularization and uncertainty quantification. An alternative approach to high-dimensional data is partitioning features into biologically informed subgroups, fitting submodels, and aggregating predictions via stacking. A non-negative spike-and-slab LASSO model [7] serves as the super learner, but its effectiveness depends on selecting appropriate base learners to prevent overfitting.

### Related work

Two widely used shrinkage methods are Ridge regression and LASSO [8], which focus on the linear dependency between input features and outcome variables and are known for interpretability. Ridge regression minimizes the combination of the residual sum of squares (RSS) and a penalty $\lambda \sum _{j=1}^{p}\beta _{j}^2$, controlled by $\lambda$. Here, $\beta _{j}$ are the linear model coefficients of interest. The penalty term tends to shrink all coefficients toward zero. In contrast, LASSO minimizes $\text {RSS} + \lambda \sum _{j=1}^{p}|\beta _{j}|$. The penalty term encourages sparsity in the model by setting some coefficients exactly to zero, effectively performing variable selection. Group LASSO [9] extends this idea by encouraging sparsity at the group level, making it suitable for structured data domains such as genomics or image analysis—an idea we adopt in this work.

A probabilistic alternative, Bayesian LASSO [8, 10] incorporates a shrinkage prior for coefficients that enforce sparsity while quantifying uncertainty. It is instrumental in limited data settings but can be computationally demanding for large datasets. The choice of prior distributions can further introduce subjectivity, and the results may depend on the chosen prior specifications. Popular shrinkage priors, including the Laplace, horseshoe, and spike-and-slab priors [11, 12], enforce sparsity but differ in shrinkage behavior, adaptability, and assumptions. Chief among them is spike-and-slab prior, which offers a structured approach to variable selection in high-dimensional data by shrinking irrelevant features toward zero while preserving relevant ones with nonzero coefficients.

**Fig. 1:**
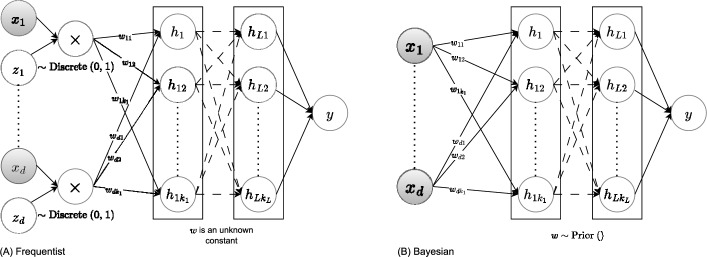
Illustrates state-of-the-art Frequentist (**A**) vs. Bayesian (**B**) neural network approaches for modeling sparsity. In **A**, we sample stochastic gate indicators *z*’s via truncated Gaussian [14] or logistic [15] and learn the unknown network weights and biases via maximizing the likelihood. In **B**, we assume priors such as horseshoe [16] and Spike-and-Slab [17] for the network weights and biases, and learn them via posterior computation

Deep neural network models have shown promising performance in cancer prediction using gene sequencing data [13]. However, their black-box nature poses a major challenge in identifying key genes driving specific predictions, limiting their clinical interpretability. Thus, there is a need for interpretable models that medical professionals can rely upon. Recent studies have proposed a range of neural network–based approaches aimed at modeling sparsity and performing non-linear feature selection. These models—summarized in Fig. 1—embody different design choices and methodological innovations, which we group into frequentist and Bayesian perspectives.

From a frequentist perspective—Part 1A, [15] approximate the $l_0$ penalty on the network parameters using stochastic gate variables (which are differentiable and non-negative) to enable direct weight selection. They employ the *Hard Concrete* (HC) distribution, a modified binary concrete distribution [18, 19], with the parameters $\phi = (\log \alpha , \beta )$. Here, $\alpha$ is a learnable location parameter determining whether the gate is active. This distribution is stretched to the $(\tau , \zeta )$ interval with $\tau < 0$ and $\zeta > 1$, and then a hard sigmoid is applied. For each dimension *j*, we have the gate $z_{j} = \min (1, \max (0, \overline{s}))$ where $\overline{s} = s(\zeta - \tau ) + \tau$, and $s = {{\,\textrm{Sigmoid}\,}}((\log u - \log (1-u) + \log \alpha ) / \beta )$. This is based on logistic distribution, which has a heavier tail than the frequently used Gaussian distribution, resulting in instability during training [14].

Building on this idea, [14, STG] propose an embedded nonlinear feature selection method, employing stochastic gates to the input layer of a neural network. Based on relevance, the stochastic gates activate (1) or deactivate (0) each input feature to the rest of the network. STG uses Gaussian-based continuous relaxation for the Bernoulli random variables, termed stochastic gates, as follows. For each dimension *d*, we have the gate $z_{j} = \max (0, \min (1, \mu _{j} + \epsilon _{j}))$ where $\epsilon _{j} \sim \mathcal {N}(0, \sigma ^2)$, $\sigma$ is a user-defined parameter, $\mu _j$ is a learnable parameter.

In contrast, Bayesian Neural Networks (BNNs) [6, 20] offer a principled probabilistic framework by placing distributions over model parameters and predictions, enabling uncertainty quantification and improving robustness, particularly in data-scarce settings; see Part 1B. However, traditional BNNs face computational complexity and scalability challenges, limiting their applicability to large datasets and complex models. Techniques such as sparse network selection through discrete priors [16] offer a solution, but computational inefficiencies persist. Variational methods emerged as a natural solution in such a setting [21], transforming high-dimensional Bayesian posterior problems into tractable optimization frameworks with a variational distribution that can be efficiently solved using methods like backpropagation.

Structured sparsity techniques in neural networks: node and edge selection enable dimension reduction, enhance computational efficiency, and reduce storage requirements. Priors like spike-and-slab or horseshoe induce sparsity by shrinking irrelevant feature coefficients toward zero, preventing overfitting in high-dimensional settings and guiding feature selection. For instance, [17, sBNN] leverage spike-and-slab priors with the Dirac spike function on network weights for inducing sparsity across all network layers. This prior helps to activate or deactivate an edge while training.

Node selection in Bayesian frameworks [16, 22] often relies on ad-hoc pruning (e.g., threshold-based) that requires fine-tuning. Ghosh et al. [16] use Bayesian neural networks with a regularized horseshoe prior [23], $w \sim \mathcal {N}(0, c^2\mathbb {I})$, where $\mathbb {I}$ is the identity matrix and *c* acts as a weight-decay hyperparameter. This regularization softly truncates heavy tails by penalizing large weights through $\mathcal {N}(0, c^2\mathbb {I})$. Variational inference is performed via non-centered reparametrization of the Cauchy distribution to avoid high-variance gradients from thick tails. Although theoretically appealing, the regularized horseshoe prior offers guarantees for shrinkage rather than explicit feature selection. Neural net edge selection has been studied in theory and practice [17, 24, 25], but gaps remain due to rigid network structure assumptions. Bergen et al. [26] employs a two-layer neural network with a spike-and-slab prior on the first-layer weights, using MCMC for inference. However, it is not a deep neural network, is not motivated by the group LASSO framework, and lacks convergence guarantees. Moreover, the reliance on MCMC sampling renders the approach computationally expensive, limiting its scalability to large-scale genomic datasets and extensions to deeper network architectures.

Sparse BNNs have been studied theoretically from frequentist and Bayesian perspectives [27–30]. Schmidt-Hieber [29] analyzed the approximation error of sparsely connected neural networks for Holder smooth functions. Sun et al. [30] proposed a frequentist-like method for learning sparse DNNs and justified its consistency within a Bayesian framework. Liang et al. [27] and Polson and Ročková [28] demonstrated posterior consistency for BNNs under some restrictive conditions.

***Paper contributions and novelty***

We propose a novel weight-sharing Bayesian neural network (wsBNN) for feature selection, drawing inspiration from the principles of group LASSO [e.g. 9, 31]. We employ a spike-and-slab prior to the weights originating from the input nodes, where the spike is a Dirac delta function, and the slab is a normal distribution. This prior encourages sparsity by shrinking the weights of irrelevant features to zero, effectively facilitating feature selection. We adopt a normal prior on the weights for the subsequent feedforward neural network layers. Unlike previous works [e.g. 17], which assume equal-width network structures, our model imposes no architectural restrictions, offering greater flexibility and aligning more closely with theoretical principles.

In wsBNN, all connections from a given feature (e.g., gene BRCA1) to the first hidden layer share a common weight distribution with shared parameters. This design (a) uniformly propagates each feature’s influence across all hidden nodes, which further enhances feature selection stability and interpretability, and (b) reduces trainable weights, mitigating overfitting in high-dimensional, low-sample-size settings.

Additionally, we establish the consistency of the variational posterior in the proposed wsBNN model by outlining key conditions and providing mathematical guarantees. Specifically, we derive a bound on the covering number for wsBNN, which aids in theoretical analysis. We further establish bounds that differentiate the true distribution from an alternate variational distribution over the parameter space, ensuring that the prior places sufficient mass around the true parameter. Moreover, we demonstrate that the variational family is expressive enough to approximate the true posterior within a Kullback–Leibler (KL) neighborhood.

Empirical evaluations on simulated data, benchmark datasets from the feature selection literature, and the TCGA cancer gene expression dataset demonstrate the effectiveness of wsBNN. Our model achieves competitive predictive performance compared to state-of-the-art feature selection methods while exhibiting greater consistency in feature selection across multiple runs. Moreover, in the analysis of gene expression data, wsBNN demonstrates a strong ability to identify biologically meaningful and cancer-specific genes, including HORMAD1, AGR3, ESR1, and ANKRD30A, which are well-established markers of breast cancer, outperforming a nonlinear feature selection baseline.

Reactome pathway analysis revealed that wsBNN-identified genes were significantly enriched in ERBB2- and PI3K/AKT-associated signaling cascades, including Signaling by ERBB2 in Cancer, PI3K/AKT Signaling in Cancer, and PIP3 activates AKT signaling. Although wsBNN did not necessarily identify the ERBB2 or AKT genes themselves, its selected feature set mapped to multiple components of these pathways—indicating that wsBNN captures functionally coherent molecular processes central to HER2-driven and PI3K/AKT-mediated oncogenesis in breast cancer. Complementary GO enrichment analysis highlighted processes related to immune response, cell cycle regulation, and protein autophosphorylation, reinforcing the model’s sensitivity to core cancer biology. Collectively, wsBNN demonstrated superior biological interpretability and robustness compared to STG and RF, which emphasized developmental and DNA-repair-related pathways, respectively.

***Paper organization*** Next, in Sect. 3, we present an overview of nonparametric regression and classification using neural networks and Bayesian neural networks. The proposed model, wsBNN (Sect. 3.2), is then introduced, highlighting its prior and posterior formulations and comparing it to related frequentist and Bayesian approaches. Posterior inference, described in Sect. 3.3, employs mean-field variational inference for wsBNN, with Gumbel-Softmax for discrete variable approximation. The theoretical properties of wsBNN’s variational posterior, including consistency as the sample size increases, are investigated in Sect. 3.4. Section 4 evaluates wsBNN on simulated and real-world datasets, comparing its feature selection performance with baseline methods. Also, a case study on BRCA gene expression demonstrates the model’s practical and biological relevance, supported by pathway and functional enrichment analysis. Section 5 discusses the contributions, addresses limitations, and suggests directions for future research.

## Methods

### Neural network regression and classification

The nonparametric regression model with *d*-dimensional input variable ${\boldsymbol{X}}\in \mathcal{X} \subseteq \mathbb {R}^d$ and outcome variable $Y \in \mathcal{Y} \subseteq \mathbb {R}$ is defined as

2.1 $$\begin{aligned} Y = f_0({\boldsymbol{X}}) + \epsilon , \quad \epsilon \sim {{\,\mathrm{\mathcal {N}}\,}}(0,\sigma ^2) \end{aligned}$$

where $f_0:\mathcal {X} \rightarrow \mathcal {Y}$. Let $\mathcal {D} = \{({\boldsymbol{x}}_1,y_1), ({\boldsymbol{x}}_2,y_2), \ldots , ({\boldsymbol{x}}_n,y_n)\}$ represent the dataset comprising *n* observations, where ${\boldsymbol{x}}_i$ denotes the input vector and $y_i$ represents the corresponding response variable. Given the model, we write the conditional density of $Y = y \,|\,{\boldsymbol{X}}={\boldsymbol{x}}$ as

2.2 $$\begin{aligned} p_0(y|{\boldsymbol{x}}) = \frac{1}{\sqrt{2\pi \sigma ^2}} \exp \left( - \frac{y-f_0({\boldsymbol{x}})^2}{2\sigma ^2}\right) . \end{aligned}$$

Artificial neural networks are known to be good function approximators [32]. We define a neural network with *L* hidden layers to approximate $f_0$ in (2.1). For each hidden layer $l = 1, \ldots , L$, $k_l$ denotes the number of neurons in the layer. We use $W_l \in \mathbb {R}^{k_{l-1}\times k_l}$ and $b_l \in \mathbb {R}^{k_l}$ to denote the weight matrix and bias parameter of layer *l*. We then write the neural network as a mapping function, $f_{\boldsymbol{\theta }}: \mathcal {X} \rightarrow \mathcal {Y}$, of network parameters,

2.3 $$\begin{aligned} f_{\boldsymbol{\theta }}({\boldsymbol{x}}) = W_{L+1} \psi (W_L \psi (\ldots \psi (W_1 {\boldsymbol{x}}+ b_1) \ldots + b_{L-1}) + b_L) + b_{L+1}, \end{aligned}$$

where $\boldsymbol{\theta }= (\theta _1,\ldots ,\theta _T)'$ is a vector containing all coefficients $W_l$’s and $b_l$’s, $T:= \sum _{l=1}^{L-1}k_{l+1}(k_l+1) + k_1(k_0+1)+(k_L+1)$, $k_0=d$, $k_{L+1}=1$, and $\psi (x)$ represents the activation function. Approximating the true function $f_0$ by the neural network function $f_{\boldsymbol{\theta }}$ induces the following conditional density

2.4 $$\begin{aligned} p_{\boldsymbol{\theta }}(y|{\boldsymbol{x}}) = \frac{1}{\sqrt{2\pi \sigma ^2}} \exp \left( - \frac{y-f_{\boldsymbol{\theta }}({\boldsymbol{x}})^2}{2\sigma ^2}\right) . \end{aligned}$$

The data likelihood under the neural network model and the true model is given by (assuming independence)

2.5 $$\begin{aligned} \ell _{{{\,\mathrm{\mathcal {D}}\,}}}({\boldsymbol{\theta }}) = \prod _{i=1}^{n} p_{\boldsymbol{\theta }}(y_i|{\boldsymbol{x}}_i), \quad \quad \ell _{{{\,\mathrm{\mathcal {D}}\,}}}(\boldsymbol{\theta }_0) = \prod _{i=1}^{n} p_0(y_i|{\boldsymbol{x}}_i). \end{aligned}$$

For neural network classification, we consider a network similar to the regression setting but tailored to output probabilities for class labels. Let $\mathcal {C}$ be the set of possible classes. The neural network output for classification is formulated by: $\hat{p}_{\boldsymbol{\theta }}({\boldsymbol{x}}) = \text {Softmax}(f_{\boldsymbol{\theta }}({\boldsymbol{x}}))$, where $\hat{p}_{\boldsymbol{\theta }}({\boldsymbol{x}}) = ( \hat{p}_{\boldsymbol{\theta }, 1}({\boldsymbol{x}}), \hat{p}_{\boldsymbol{\theta }, 2}({\boldsymbol{x}}), \ldots )$ is a vector of probabilities for each class $c \in \mathcal {C}$. The corresponding likelihood function for the neural network classification model and true classification model is given by:

2.6 $$\begin{aligned} \ell _\mathcal {D}(\boldsymbol{\theta }) = \prod _{i=1}^{n} \prod _{c \in \mathcal {C}} \hat{p}_{\boldsymbol{\theta }, c}({\boldsymbol{x}}_i)^{\mathbb {I}(y_i = c)} , \quad \quad \ell _{{{\,\mathrm{\mathcal {D}}\,}}}(\boldsymbol{\theta }_0) = \prod _{i=1}^{n} \prod _{c \in \mathcal {C}} p_{0, c}({\boldsymbol{x}}_i)^{\mathbb {I}(y_i = c)}, \end{aligned}$$

where $\mathbb {I}(.)$ is the indicator function.

In Bayesian neural networks [e.g. 6, 20, BNN], network parameters—weights and biases, are treated as random variables with prior distributions for modeling uncertainty in parameters. Specifically, we assume a prior distribution $\pi (\boldsymbol{\theta })$ over the parameters $\boldsymbol{\theta }$. The posterior distribution given the data $\mathcal {D}$ is obtained via Bayes’ theorem:

2.7 $$\begin{aligned} \nu (\boldsymbol{\theta }\,|\,\mathcal {D}) = \frac{\ell _\mathcal {D}(\boldsymbol{\theta }) \, \pi (\boldsymbol{\theta })}{m(\mathcal {D})}, \end{aligned}$$

where $\ell _\mathcal {D}(\boldsymbol{\theta })$ is the likelihood of the data given parameters $\boldsymbol{\theta }$ as in (2.5) or (2.6), and $m(\mathcal {D})$ is the marginal likelihood.

BNNs have gained attention for modeling prediction uncertainties and supporting robust decision-making. However, traditional BNNs face scalability challenges due to their high parameter demands. To address this, we propose feature selection within BNNs by assessing the relevance of each feature $x_i$. By optimizing the prior structure for network parameters, the model computes the posterior inclusion probability of each feature, indicating its relevance (see Sect. 3.2 for details). BNNs offer a robust framework for feature selection by incorporating parameter uncertainty and enabling probabilistic inference for regression and classification, with likelihoods tailored to the response variable.

### Weight-sharing Bayesian Neural Network (wsBNN)

**Fig. 2:**
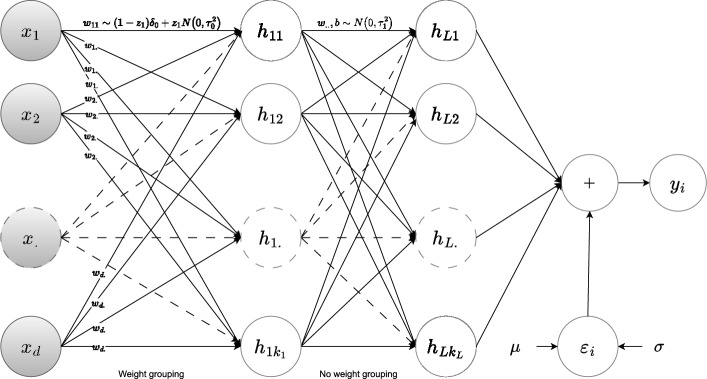
Weight-sharing Bayesian neural network, via a shared inclusion variable $z_i$ and spike-and-slab prior for each input feature *i*. Biases $\boldsymbol{b}$ are omitted for clarity in the illustration

*Sparsity inducing prior.* We propose a neural network (see Fig. 2 for illustration) that performs feature selection by adapting the weights from input nodes in the first layer as follows. Let $z_{i} \in \{0, 1\}$ be a binary variable indicating the significance of the $i^\text {th}$ input node or a feature. Specifically, a value of $z_{i} = 1$ indicates that the node is significant and should be included in the forward pass, while $z_{i} = 0$ means it is excluded. We assume that weights originating from each input node follow a spike-and-slab prior [11, 12] with shared parameters. For the $i^\text {th}$ node in the input layer, we have

2.8 $$\begin{aligned} (a) \quad z_i \sim {{\,\textrm{Bernoulli}\,}}(\lambda _0), \qquad (b) \quad w^{(0)}_{ij} \,|\,z_i \sim (1-z_i) \, \underbrace{\delta _0}_\text {spike} + z_i \, \underbrace{{{\,\mathrm{\mathcal {N}}\,}}(0, \tau _0^2)}_\text {slab} \end{aligned}$$

The weight $w^{(0)}_{ij}$ connects the $i^\text {th}$ input node to $j^\text {th}$ node in the first hidden layer. The spike component $\delta _0$, a Dirac delta function centered at 0, assigns all probability mass to $w^{(0)}_{i.} = 0$, and becomes active when $z_i = 0$. The slab component, on the other hand, follows a Gaussian distribution with zero mean and variance $\tau _0^2$, and becomes active when $z_i = 1$. The binary variable $z_i$ is sampled once for a node *i* and governed by the inclusion probability $\lambda _0$, a user-defined parameter that controls the mixing between the spike and slab components. When node *i* is selected by the model, i.e., $z_i = 1$, the slab component is activated, and the weights $w_{i.}$ originating from node *i* share the same Gaussian prior.

All nodes, except those in the input layer, are fully connected, and their corresponding weights and biases follow a Gaussian prior. For the $i^\text {th}$ node in layer $l > 0$, we then have the weight and bias as

2.9 $$\begin{aligned} w^{(l)}_{ij}, b_{i}^{(l)} \sim {{\,\mathrm{\mathcal {N}}\,}}(0, \tau _1^2), \end{aligned}$$

where $w^{(l)}_{ij}$ represents the weight connecting the $i^\text {th}$ node in layer *l* to every $j^\text {th}$ node in layer *l*+1.

This can be interpreted as selecting the Gaussian slab with probability 1 (i.e., an inclusion probability $\lambda _0 = 1$), in a spike-and-slab setting (2.8).

The weights connecting the input layer to the first hidden layer of the neural network (Fig. 2) are drawn from the spike-and-slab prior (2.8), while the weights and biases in the remaining layers follow a normal prior (2.9). This distinction in prior distributions enables feature selection in the first layer, where the spike-and-slab prior determines whether specific features are selected or ignored. Inspired by the group LASSO, the wsBNN model groups weights connecting each feature $x_{i}$ to all nodes in the first hidden layer, as governed by the spike-and-slab prior (2.8) indexed by a single inclusion variable $z_i$. This shared weighting scheme ensures that the contribution of each feature $x_i$ is considered collectively across all nodes in the first hidden layer.

Group LASSO promotes sparsity at the group level by organizing related features into groups and applying a shared regularization penalty to each group, while still allowing individual feature weights within the group to vary. In contrast, using spike-and-slab priors with a single inclusion variable, wsBNN enforces weight-sharing for all weights (connections) originating from a single input feature. This design ensures that all weights linked to the same feature are governed by a common relevance indicator, which effectively assigns a unified importance to all hidden nodes. As a result, the relationship between input features and their contribution to the model remains explicit and interpretable, eliminating the need for post-hoc feature attribution methods.

In contrast to wsBNN, STG [14] neither incorporates weight sharing in the first layer nor benefits from the uncertainty quantification and regularization advantages of Bayesian modeling. The baseline Bayesian model, sBNN [17], applies spike-and-slab priors across all network weights, enforcing sparsity throughout the architecture. While this strategy is comprehensive, it considerably increases computational complexity. wsBNN model takes a different approach: (i) we use the spike-and-slab prior exclusively in the first layer, employing inclusion variables for grouping, which emphasizes feature-level selection rather than imposing global sparsity, and (ii) our architecture is flexible, permitting varying widths (i.e., the number of nodes) for hidden layers. In contrast, sBNN assumes equal-width layers across the entire network.

*Posterior.* Let $\boldsymbol{\theta }= ({\boldsymbol{w}}^{(0)}, {\boldsymbol{w}}^{(0)}, \ldots , {\boldsymbol{w}}^{(L)}, \boldsymbol{b})$ be the neural network weights and biases, and ${\boldsymbol{z}}$ be the vector of indicator variables. We can write the nonparametric regression model induced by the proposed neural network as

$$\begin{aligned} Y = f_{\boldsymbol{\theta }}({\boldsymbol{X}}) + \epsilon , \quad \epsilon \sim {{\,\mathrm{\mathcal {N}}\,}}(0,\sigma ^2)\end{aligned}$$

See (2.4) for the corresponding density. Let $\pi (\boldsymbol{\theta },{\boldsymbol{z}}) = \pi (\boldsymbol{\theta }\,|\,{\boldsymbol{z}}) \pi ({\boldsymbol{z}})$ be the joint distribution of $\boldsymbol{\theta }$ and ${\boldsymbol{z}}$ representing the prior induced by (2.8) and (2.9).

Given the likelihood function $\ell _\mathcal {D}(\boldsymbol{\theta })$ in (2.5) and the observed data $\mathcal {D}$, the posterior distribution of $(\boldsymbol{\theta }, {\boldsymbol{z}})$ is given by

2.10 $$\begin{aligned} \nu (\boldsymbol{\theta },{\boldsymbol{z}}\,|\,\mathcal {D}) = \frac{\ell _\mathcal {D}(\boldsymbol{\theta }) \, \pi (\boldsymbol{\theta },{\boldsymbol{z}})}{\sum _{{\boldsymbol{z}}} \int \ell _\mathcal {D}(\boldsymbol{\theta }) \, \pi (\boldsymbol{\theta },{\boldsymbol{z}}) d\boldsymbol{\theta }} = \frac{\ell _\mathcal {D}(\boldsymbol{\theta }) \, \pi (\boldsymbol{\theta },{\boldsymbol{z}})}{m(\mathcal {D})} \end{aligned}$$

Let $A \subseteq \Theta$, where $\Theta$ is the parameter space of $\boldsymbol{\theta }$. We define the prior probability distribution function $\pi (A)$

2.11 $$\begin{aligned} \pi (A) = \int _{\boldsymbol{\theta }\in A} \pi (\boldsymbol{\theta }) d\boldsymbol{\theta }, \end{aligned}$$

corresponding to the marginal prior $\pi (\boldsymbol{\theta }) = \sum _z \pi (\boldsymbol{\theta },{\boldsymbol{z}})$.

Similarly, we define the probability distribution function of the posterior as

2.12 $$\begin{aligned} \nu (A \,|\,\mathcal {D}) = \int _{\boldsymbol{\theta }\in A} \nu (\boldsymbol{\theta }\,|\,\mathcal {D}) d\boldsymbol{\theta }\end{aligned}$$

corresponding to the marginal posterior $\nu (\boldsymbol{\theta }\,|\,\mathcal {D}) = \sum _{{\boldsymbol{z}}} \nu (\boldsymbol{\theta }, {\boldsymbol{z}}\,|\,\mathcal {D})$.

### Sparse Bayesian learning of neural network parameters

In the Bayesian procedure, we make statistical inferences from the posterior of the parameters of interest. In complex probabilistic models, especially those with high-dimensional parameter spaces, calculating the exact posterior distribution is intractable due to the need to evaluate complex integrals. Specifically, the joint posterior of interest, $\nu (\boldsymbol{\theta },{\boldsymbol{z}}| \mathcal {D})$, where $\boldsymbol{\theta }$ denotes the model parameters and ${\boldsymbol{z}}$ denotes latent variables, cannot be computed in closed form. The normalization constant necessitates integrating over all possible values of $\boldsymbol{\theta }$ and ${\boldsymbol{z}}$, which becomes computationally prohibitive.

Various approximate posterior inference methods, such as MCMC and Variational Bayes [33, VB], are employed to tackle this intractability. MCMC methods generate samples from the posterior by constructing a Markov chain with the desired posterior as its equilibrium distribution. However, they can be computationally expensive and slow to converge, particularly for high-dimensional models with complex posteriors. In contrast, VB approximates the intractable posterior with a simpler, parameterized variational distribution, transforming the inference problem into an optimization task. VB is typically faster, scalable to large datasets, and well-suited for neural networks centered on a latent variable framework [21, 34], as discussed here. We employ the mean-field variational inference,

where the variational distribution $q(\boldsymbol{\theta }) \in \mathcal {Q}^\text {MF}$ over parameters $\boldsymbol{\theta }$ is factorized into independent distributions $q_i (\theta _i)$ for each parameter $\theta _i$ as $q(\boldsymbol{\theta }) = \prod _{i=1}^{T} q_i (\theta _i)$.

We assume that the variational family $\mathcal {Q}^\text {MF}$ follows a spike-and-slab family as follows. Each weight $\theta _{ij}$, originating from $i^\text {th}$ input feature, is distributed as

2.13 $$\begin{aligned} (a) \quad \quad z_i \sim {{\,\textrm{Bernoulli}\,}}(\lambda _i) \qquad (b) \quad \theta _{ij} \,|\,z_i \sim (1-z_i) \, \delta _0 + z_i \, {{\,\mathrm{\mathcal {N}}\,}}(\mu _{ij}, \tau _{ij}^2), \end{aligned}$$

where $\lambda _i \in (0, 1), i = 1, 2, \ldots , d, j = 1, 2, \ldots , k_1$. We define $T_0:= k_0 \cdot k_1$ and $T_r:= \sum _{l=1}^{L}k_{l+1} \cdot (k_l+1)+(k_L+1)$. Each parameter $\theta _i, i = 1, 2, \ldots , T_r$—weights and biases for all layers except the ones defined in (2.13)—follow

2.14 $$\begin{aligned} \theta _{i} \sim {{\,\mathrm{\mathcal {N}}\,}}(\mu _i, \tau _i^2), \end{aligned}$$

i.e., the spike-and-slab prior with the Gaussian slab with probability 1. We denote the variational parameters by $\phi _i = (\lambda _i, \mu _i, \tau _i)$. The variational distribution, also referred to as a recognition model [21], is implemented here as a neural network. We outline a method for jointly learning the parameters of both the recognition model (encoder) and the true posterior (decoder).

Our objective is to find a variational distribution $q_\phi (\boldsymbol{\theta }, {\boldsymbol{z}}) \in \mathcal {Q}^\text {MF}$ that minimizes the Kullback–Leibler (KL) divergence—which we denote by ${{\,\mathrm{d_{\text {KL}}}\,}}$—between the variational posterior $q_\phi (\boldsymbol{\theta }, {\boldsymbol{z}})$ and the true posterior $\nu (\boldsymbol{\theta },{\boldsymbol{z}}\,|\,\mathcal {D})$,

2.15 $$\begin{aligned} q_\phi ^\star (\boldsymbol{\theta }, {\boldsymbol{z}}) = \mathop {\mathrm {arg\,min}}\limits _{q_\phi (\boldsymbol{\theta }, {\boldsymbol{z}}) \in \mathcal {Q}^\text {MF}} {{\,\mathrm{d_{\text {KL}}}\,}}(q_\phi (\boldsymbol{\theta }, {\boldsymbol{z}}) \,\Vert \,\nu (\boldsymbol{\theta },{\boldsymbol{z}}\,|\,\mathcal {D})). \end{aligned}$$

However, this objective is challenging to compute because it requires computing the evidence, *m*(*D*) (2.10), which is intractable. Thus, we work on an objective that is equivalent to the one in (2.15), up to an added constant with respect to *q*, the negative of the Evidence Lower Bound [see Supplementary Section S2 of 35, ELBO], given by

2.16 $$\begin{aligned} \text {ELBO}(q) = \mathbb {E}_{q_\phi (\boldsymbol{\theta }, {\boldsymbol{z}})}\left[ \log \ell _\mathcal {D}(\boldsymbol{\theta })\right] - {{\,\mathrm{d_{\text {KL}}}\,}}(q_\phi (\boldsymbol{\theta }, {\boldsymbol{z}}) \,\Vert \,\nu (\boldsymbol{\theta },{\boldsymbol{z}})), \end{aligned}$$

The ELBO is a sum of the expected log-likelihood of the data under the model and the regularization term that penalizes the complexity of the approximate posterior against the prior. The first expectation in (2.16) is also called the reconstruction error [21] in auto-encoder literature. We can expand the second expectation as [35, Section S2]

2.17 $$\begin{aligned} \begin{aligned} {{\,\mathrm{d_{\text {KL}}}\,}}(q_\phi (\boldsymbol{\theta }, {\boldsymbol{z}}) \,\Vert \,\nu (\boldsymbol{\theta },{\boldsymbol{z}}))&= \sum _{i=1}^{T_0} {{\,\mathrm{d_{\text {KL}}}\,}}(q(z_i) \,\Vert \,\pi (z_i)) \\&\quad + \sum _{i=1}^{T_0} q(z_i = 1){{\,\mathrm{d_{\text {KL}}}\,}}\left( {{\,\mathrm{\mathcal {N}}\,}}(\mu _i, \tau ^2_i)\,\Vert \,{{\,\mathrm{\mathcal {N}}\,}}(0,\tau ^2_0)\right) \\&\quad + \sum _{i=1}^{T_r}{{\,\mathrm{d_{\text {KL}}}\,}}\left( {{\,\mathrm{\mathcal {N}}\,}}(\mu _i, \tau ^2_i)\,\Vert \,{{\,\mathrm{\mathcal {N}}\,}}(0,\tau ^2_1)\right) \end{aligned} \end{aligned}$$

The first two terms serve as regularizers for weight learning in the first layer, while the last term acts as a regularizer for the remaining layers. Closed-form expression are available for both divergence components: the KL divergence between Bernoulli distributions for ${{\,\mathrm{d_{\text {KL}}}\,}}\left( q(z_i) \,\Vert \,\pi (z_i) \right)$ and the KL divergence between Gaussian distributions ${{\,\mathrm{d_{\text {KL}}}\,}}\left( {{\,\mathrm{\mathcal {N}}\,}}(\mu _i, \tau ^2_i) \,\Vert \,{{\,\mathrm{\mathcal {N}}\,}}(0,\tau ^2_{0, 1})\right)$, [35, Section S2].

The wsBNN model takes the negative of the ELBO as the loss function $\Omega$.

The approximation error arising from the KL divergence between the true posterior and the restricted family of approximating distributions may impact posterior inference and prediction accuracy (Sect. 3.4).

***Reparametrization of stochastic variables*** Typically, the reconstruction error is estimated using Monte Carlo expectation, while regularization terms are computed analytically where possible [21]. We reparameterize distributions in (2.16): random variable $w \sim q_\phi (w | \cdot )$ as a deterministic transformation $w = g_\phi (\zeta , \cdot )$, where $\zeta$ is an auxiliary random variable with independent marginal density, and $g_\phi$ is a differentiable function parameterized by $\phi$. The normal random variables, ${{\,\mathrm{\mathcal {N}}\,}}(\mu , \sigma ^2)$, in wsBNN, for example, are reparametrized by the linear function $\mu + \zeta \cdot \sigma ; \quad \zeta {\mathop {\sim }\limits ^{\text {iid}}}{{\,\mathrm{\mathcal {N}}\,}}(0, 1)$. This approach enables efficient gradient back-propagation and optimization of $\Omega$ via stochastic gradients [21].

The above reparameterization strategy is quite practical for evaluating stochastic objectives in models with certain continuous latent variables. However, computing the regularizer term in (2.17) requires evaluation over discrete variables $z_i \in \{0,1\}$, which poses challenges for gradient-based optimization due to the undefined gradients of discrete variables. An option is to approximate $z_i$ in a continuous form, allowing for gradient back-propagation. A popular approach for this is the Gumbel-Softmax reparameterization [18, 19], which approximates the discrete variable $z_i$ by a continuous variable $\widetilde{z_i}$ derived from the Gumbel-Softmax $(\lambda _i, \gamma )$ distribution, preserving an approximation of discrete behavior, as follows.

2.18 $$\begin{aligned} \widetilde{z_i} = {\left( 1+ \exp {\left( -\frac{\eta _i}{\gamma } \right) } \right) }^{-1}, \quad \quad \eta _i = \log {\frac{\lambda _i}{1-\lambda _i}} + \log {\frac{u_i}{1-u_i}}, \quad \quad u_i \sim \mathcal {U}(0, 1) \end{aligned}$$

Consequently, we replace the term $z_i$ in (2.17) with $\widetilde{z_i}$ to compute the ELBO, enabling smooth gradients for back-propagation, for VB inference.

The distribution described in (2.18) is parameterized by the temperature $\gamma$, which modulates the degree of approximation: as $\gamma \rightarrow 0$, $\widetilde{z_i}$ approximates a binary indicator, while higher $\gamma$ yields a smoother, continuous approximation. By applying the Gumbel-Softmax trick, we retain the stochastic nature of discrete sampling within a differentiable framework, maintaining coherence with the Bayesian framework.

The Gumbel-Softmax reparameterization introduces approximation error because $\widetilde{z_i}$ remains a continuous variable even as $\gamma \rightarrow 0$. While lower $\gamma$ makes $\widetilde{z_i}$ closer to a binary output (approaching hard thresholds near 0 and 1), the approximation never fully captures the binary nature of the discrete distribution exactly. This mismatch can affect model accuracy, as the smoothed values do not perfectly emulate the hard 0 or 1 outcomes of true Bernoulli samples. Our sensitivity analysis over $\gamma$ indicates that model performance is largely robust to this approximation (Sect. 4.2).

### Consistency of the variational posterior

In Bayesian inference, we aim to draw conclusions about a parameter $\boldsymbol{\theta }$ made under the posterior distribution, $\nu (\boldsymbol{\theta }\,|\,{{\,\mathrm{\mathcal {D}}\,}})$, given observed data ${{\,\mathrm{\mathcal {D}}\,}}$ under a specified model. The posterior $\nu (\boldsymbol{\theta }\,|\,{{\,\mathrm{\mathcal {D}}\,}})$ is considered consistent if it eventually concentrates as a point mass around the true data-generating parameter $\boldsymbol{\theta }_0$, as the sample size *n* grows, i.e., $n \rightarrow \infty$. This implies that the posterior distribution increasingly concentrates in neighborhoods of $\boldsymbol{\theta }_0$, reflecting growing certainty about the true parameter.

For consistent estimators, we are also interested in “how fast” this consistency happens, i.e., “the rate of convergence”. One way to define posterior consistency is in terms of neighborhoods around $\delta _{\boldsymbol{\theta }_0}$, the point mass at $\boldsymbol{\theta }_0$. An example is the Hellinger neighborhood ${{\,\mathrm{\mathbb {H}}\,}}_{\epsilon } (\delta _{\boldsymbol{\theta }_0})$ [35, Supplementary Section S1]. We provide an example in the supplement [35] to illustrate the theoretical setup and related terms.

In the general theory of posterior consistency, see, e.g., [36], sufficient conditions for ensuring consistency are established using the idea of “test functions”—functions of the data that mapping to [0, 1]—in decision theory. Intuitively, for a consistent posterior, we should be able to construct test functions capable of discriminating between two scenarios: when the data is generated by the true parameter $\boldsymbol{\theta }_0$ and when it is not. In hypothesis testing, this corresponds to the hypotheses $H_0: \boldsymbol{\theta }= \boldsymbol{\theta }_0$, $H_1: \boldsymbol{\theta }\ne \boldsymbol{\theta }_0$. The aim is to have a decision rule with the errors $P[\text {reject } H_0 \,|\,H_0 \text { is true}]$ and $P[\text {accept } H_0 \,|\,H_1 \text { is true}]$ low. Let $\phi _n$ be the probability that we reject $H_0$. We want $\phi _n$ to be small when $\boldsymbol{\theta }= \boldsymbol{\theta }_0$ and high when $\boldsymbol{\theta }\ne \boldsymbol{\theta }_0$. Writing in terms of expectations, we want $\mathbb {E}_{{{\,\mathrm{\nu (\boldsymbol{\theta }_0)}\,}}}(\phi _n)$ and $\mathbb {E}_{{{\,\mathrm{\nu (\boldsymbol{\theta }^{\complement }_0)}\,}}}(1-\phi _n)$ to be close to zero, where ${{\,\mathrm{\nu (\boldsymbol{\theta }_0)}\,}}$ is the true posterior of interest. Furthermore, [37, 38] study that the existence of such test functions is pivotal for deriving posterior contraction rates. These test functions form a flexible framework for expressing convergence properties and analyzing the behavior of posterior distributions, as we presented in this section.

*Definitions.* We define two parameters: ${\boldsymbol{s}}= (s_1, \ldots , s_L)$, representing layer-wise sparsity, i.e., number of non-zero weights (or connections) in each layer, and ${\boldsymbol{B}}= (B_1, \ldots , B_L)$, representing layer-wise constraints on $L_1$ norms of weights and biases. For the remaining layers except the first layer, we use $s_2 = k_2 \cdot k_3, \ldots , s_L=k_L \cdot k_{L+1}$ (recall $k_l$ is the number of hidden nodes in a layer *l*). We define the sieve ( Section S1 in [35]; adapted from [39]) of neural networks with respect to ${\boldsymbol{s}}$ and ${\boldsymbol{B}}$ as:

2.19 $$\begin{aligned} {{\,\mathrm{\mathcal {F}}\,}}_{\zeta } = \{ f_{\boldsymbol{\theta }}({\boldsymbol{x}}) : {\Vert {\boldsymbol{w}}_l \Vert }_0 \le s_l, {\Vert {\boldsymbol{w}}_l \Vert }_\infty \le B_l \}, \end{aligned}$$

which captures the constraints on the $L_0$ and $L_\infty$ norms of the weights in each layer of the neural network, ensuring both sparsity and boundedness in weight magnitudes. Intuitively, the sieve ${{\,\mathrm{\mathcal {F}}\,}}_{\zeta }$ represents a restricted subset of neural networks within the whole parameter space—whose weights are sufficiently sparse (fewer nonzero connections) and not excessively large in magnitude. By increasing $s_l$ and $B_l$, the sieve expands to yield richer function classes that better approximate the true model while maintaining complexity control.

Let $\epsilon _n$ be a sequence. We then define the number of active connections, $s_l^\circ + 1$ (1 for bias term), the complexity of the $l^\text {th}$ layer, $u_l$, and $B_l^\circ$, for $l = 1, \ldots , L$, with respect to the proposed the neural network (for Lemma 2.2 and 2.3) as

2.20 $$\begin{aligned} s_l^\circ + 1&= \frac{n \epsilon _n^2}{ u_l}, \quad u_l = (L+1) (\log n + \log (L+1) + \log k_{l+1}) \end{aligned}$$

2.21 $$\begin{aligned} \log B_l^\circ&= \frac{n \epsilon _n^2}{(L+1) (s_l^\circ + 1)} \end{aligned}$$

As layer complexity $u_l$ increases, greater sparsity is introduced at each layer to reduce overfitting, as shown in $s_l^\circ + 1$. The term $(L+1)$ influences the network depth’s impact on its complexity, which is crucial for deep networks. Additionally, $B_l^\circ$ acts as a weight constraint, inversely scaling with sparsity: with fewer zero weights, $B_l^\circ$ imposes stricter weight norms, limiting the complexity of representations learned by each layer (Table 1).

**Table 1:** Summary of frequently used notation

$s_l$	Layer-wise sparsity for layer *l*, (2.20)
$u_l$	Layer-wise complexity for layer *l*, (2.20)
$B_l$	Constraint on $L_\infty$ norms of the weights in layer *l*, (2.21)
$\xi$	Approximation error between the true and neural network functions, (2.25)
*r*	Variational error comes by approximating true posterior with variational posterior, (2.26)
*v*	Complexity term capturing the network structure, (2.27)

Next, we aim to establish a rigorous statistical foundation for the wsBNN model by proving posterior consistency under a spike-and-slab prior with weight grouping and shared parameters in the first layer. The parameters, such as layer-wise sparsity *s*, layer complexity *u*, and weight constraints *B*, will help us in deriving the covering number bounds, which will directly influence the model’s convergence behavior.

*Existence of test functions for wsBNN.* Lemma 2.1 (Proof in the supplement [35]), presents an upper bound on the covering number, ${{\,\textrm{CN}\,}}(\delta ,{{\,\mathrm{\mathcal {F}}\,}},\Vert \cdot \Vert )$ [35, Section S1], for the proposed neural network. Intuitively, this bound quantifies the number of $\delta$-radius “balls” required to cover the function class ${{\,\mathrm{\mathcal {F}}\,}}$ (2.19) under a specified norm $\Vert \cdot \Vert$, serving as a key result for our subsequent analysis. Weight grouping with shared parameters in wsBNN reduces the effective number of parameters, eventually leading to a smaller covering number bound and improved posterior convergence. In Bayesian neural networks for sparsity, such as sBNN, each feature weight is learned independently, resulting in a larger parameter space exploration and impacting convergence. While the general framework follows [17, 29, 37, 40], the result here is tailored to the proposed model, reflecting its specific structure and constraints.

#### Lemma 2.1

*Let *$\psi$* be a 1-Lipschitz continuous activation function satisfying* $\psi (x) \le x$* for all* $x \ge 0$.* Then, for the function class* ${{\,\mathrm{\mathcal {F}}\,}}$* induced by wsBNN, the covering number* ${{\,\textrm{CN}\,}}(\delta , {{\,\mathrm{\mathcal {F}}\,}}, \Vert \cdot \Vert )$* under the norm* $\Vert \cdot \Vert$* is bounded as follows:*

2.22 $$\begin{aligned} {{\,\textrm{CN}\,}}(\delta , {{\,\mathrm{\mathcal {F}}\,}}, \Vert \cdot \Vert )&\le \left[ 2 \delta ^{-1} (L+1) \prod _{j=0}^L B_j \cdot k_1 \right] ^{s_0} \cdot \prod _{l=1}^{L} \left[ 2 \delta ^{-1} (L+1) \prod _{j=0}^L B_j \cdot k_{l+1} \right] ^{k_l \cdot k_{l+1}} \end{aligned}$$

We now present three main conditions for achieving posterior consistency in Lemmas 2.2–2.4 (Proofs and other details in the supplement [35]). We closely follow the work of [17, 29, 37–39], but we show that conditions to lead the results are tailored here to the proposed model for each result, reflecting its specific structure and constraints.

Lemma 2.2 offers bounds on a test function $\phi$ for distinguishing between the true distribution ${{\,\mathrm{\nu (\boldsymbol{\theta }_0)}\,}}$ and an alternate distribution ${{\,\mathrm{\nu (\boldsymbol{\theta })}\,}}$ over the parameter space. We obtain a constant $C_1$ and $C_2$, which is specific to the proposed network wsBNN.

#### Lemma 2.2

*Suppose we have a sequence* $\epsilon _n$* such that* $\epsilon _n \rightarrow 0$* and* $n{\epsilon _n}^2 \rightarrow \infty$,* where* *n is the sample size and* $\epsilon _n > 0$* is a small positive constant. There exist a test function* $\phi \in [0,1]$* and constants* $C_1, C_2 > 0$* such that the following inequalities hold:*

2.23 $$\begin{aligned} \mathbb {E}_{{{\,\mathrm{\nu (\boldsymbol{\theta }_0)}\,}}}[\phi ]&\le \exp (-C_1 n{\epsilon _n}^2) \end{aligned}$$

2.24 $$\begin{aligned} \sup _{\boldsymbol{\theta }\in {{\,\mathrm{\mathbb {H}}\,}}_{\epsilon _n}^\complement , \, f_{\boldsymbol{\theta }} \in {{\,\mathrm{\mathcal {F}}\,}}_{\zeta ^\circ }} \mathbb {E}_{{{\,\mathrm{\nu (\boldsymbol{\theta })}\,}}}[1-\phi ]&\le \exp (-C_2 n {{\,\mathrm{d^2_\text {H}}\,}}({{\,\mathrm{\nu (\boldsymbol{\theta }_0)}\,}}, {{\,\mathrm{\nu (\boldsymbol{\theta })}\,}})) \end{aligned}$$

*Here*, ${{\,\mathrm{\mathbb {H}}\,}}_{\epsilon _n}^\complement$* represents the complement of the Hellinger neighborhood* ${{\,\mathrm{\mathbb {H}}\,}}_{\epsilon _n} ({{\,\mathrm{\nu (\boldsymbol{\theta }_0)}\,}})$, *and* ${{\,\mathrm{\mathcal {F}}\,}}_{\zeta ^\circ }$* is defined for specific values of* ${\boldsymbol{s}}^\circ$ *and* ${\boldsymbol{B}}^\circ$.

*Prior mass conditions for the marginal prior *(2.11). An ideal prior distribution should allocate sufficient mass to the neighborhoods of the true parameter value. It also ensures that the posterior distribution concentrates around the true parameter as the sample size increases [38]. Specifically, the prior should provide adequate mass in regions relevant to the true parameter and maintain appropriately decaying tails. The conditions in Lemma 2.3 relate to sparsity and boundedness, with the sparsity aspect tailored for wsBNN, resulting in a specific constant $C_3$.

#### Lemma 2.3

*Consider the sequence* $\epsilon _n$ *such that* $\epsilon _n \rightarrow 0$, $n{\epsilon _n}^2 \rightarrow \infty$ *and* $\frac{n{\epsilon _n}^2}{u_0} \rightarrow \infty$, *then for prior* $\pi$ (2.11)* and there exists a constant* $C_3 > 0$ *such that,*

$$\begin{aligned} \pi ({{\,\mathrm{\mathcal {F}}\,}}_{\zeta ^\circ }^c) \le \exp \left\{ \frac{-C_3 n \epsilon _n^2}{u_0} \right\} \end{aligned}$$

*where* ${{\,\mathrm{\mathcal {F}}\,}}_{\zeta ^\circ }^c$* denotes the complement of the class* ${{\,\mathrm{\mathcal {F}}\,}}_{\zeta ^\circ }$.

*KL neighborhood *(${{\,\mathrm{\mathbb {K}}\,}}_\epsilon$,* with radius *$\epsilon$)* of the posterior.* ${{\,\mathrm{\mathbb {K}}\,}}_\epsilon$ of the true density function ${{\,\mathrm{\nu (\boldsymbol{\theta }_0)}\,}}$ must have sufficient probability to ensure the proposed variational posterior effectively captures the true distribution. Bounding the variational posterior’s loss function is essential for convergence, as established in Lemma 2.4. The ${{\,\mathrm{\mathbb {K}}\,}}_\epsilon$-bound is computed for wsBNN to ensure sufficient posterior mass near the true parameter value, in terms of two kinds of errors that occur in the variational BNN inference [25]: (i) the approximation error $\xi$ between the true function and the neural network

2.25 $$\begin{aligned} \xi = \min _{f_{\boldsymbol{\theta }} \in {{\,\mathrm{\mathcal {F}}\,}}_{\zeta }} \Vert f_{\boldsymbol{\theta }} - f_0 \Vert _{\infty }^{2} \end{aligned}$$

and (ii) the variational error $r_l$ introduced by approximating the true posterior with a variational distribution:

2.26 $$\begin{aligned} r_l = \frac{s_l \cdot (k_l + 1) \cdot v_l}{n} \end{aligned}$$

The error $r_l$ depends on the layer’s sparsity $s_l$, the number of neurons $k_l$, and the term $v_l$ reflects the norm constraints on weights and biases (e.g., $B_l$) and complexity (network structure) of layer *l*, given by

2.27 $$\begin{aligned} v_l = \frac{B_l^2}{(k_l+1)} + \sum _{m=0, m \ne l}^L \log B_m + L + \log k_{l+1} + \log (k_l+1) + \log n + \log \left( \sum _{m=0}^L u_m \right) . \end{aligned}$$

#### Lemma 2.4

*Suppose* $\sum _{l=0}^{L} r_l+\xi \rightarrow 0$ *and* $n\left( \sum _{l=0}^{L} r_l+\xi \right) \rightarrow \infty$ *and the following two conditions hold for the prior* $\pi$ (2.11) *and some variational distribution* $q \in \mathcal {Q}^{MF}$

2.28 $$\begin{aligned}&\pi \left( {{\,\mathrm{\mathbb {K}}\,}}_{\sum _{l=0}^{L} r_l+\xi }\right) \ge \exp \left( -C_4n\left( \sum _{l=0}^{L} r_l + \xi \right) \right) \end{aligned}$$

2.29 $$\begin{aligned}&{{\,\mathrm{d_{\text {KL}}}\,}}(q(\boldsymbol{\theta },{\boldsymbol{z}}),\pi (\boldsymbol{\theta },{\boldsymbol{z}})) + n \sum _z \int {{\,\mathrm{d_{\text {KL}}}\,}}({{\,\mathrm{\nu (\boldsymbol{\theta }_0)}\,}},{{\,\mathrm{\nu (\boldsymbol{\theta })}\,}})q(\boldsymbol{\theta },{\boldsymbol{z}}) d\boldsymbol{\theta }\le C_5 n \left( \sum _{l=0}^{L} r_l + \xi \right) \end{aligned}$$

*where* $\pi$ *denotes the prior*, *q* *denotes the variational posterior, and* ${{\,\mathrm{\mathbb {K}}\,}}_{\sum _{l=0}^{L} r_l+\xi }$ *is the KL neighborhood with radius* $\sum _{l=0}^{L} r_l+\xi$ *around the true distribution* ${{\,\mathrm{\nu (\boldsymbol{\theta }_0)}\,}}$.

*The posterior induced by the wsBNN model is consistent.* The key outcome of our development is the posterior consistency (Theorem 2.5, Proof in [35]), which refers to the property that the posterior distribution concentrates on the true parameter value as the sample size increases. The focus is to measure whether the posterior assigns high probability to neighborhoods of the true parameter value. The conditions include prior must give positive probability to neighborhoods of the true parameter (Lemmas 2.3 and 2.4 (2.28)), and the likelihood function should dominate the prior as the sample size increases, ensuring the data derive the posterior distribution (the other lemmas discussed above). Lemmas 2.2, 2.3 and 2.4 (2.28) guarantees the convergence of true posterior. Lemma 2.4 (2.29) helps in guaranteeing the convergence of the variational posterior by bounding the KL divergence between the true and the variational posterior.

#### Theorem 2.5

*Adapted from* [39, Theorem 4.4]. *Let* $q^\star (\boldsymbol{\theta }) = \sum _z q^\star (\boldsymbol{\theta }|{\boldsymbol{z}})q^\star ({\boldsymbol{z}})$ *denotes the marginal variational posterior for* $\boldsymbol{\theta }$, *with the distribution function* $q^\star (\mathcal {A}) = \int _\mathcal {A} q^\star (\boldsymbol{\theta }) d\boldsymbol{\theta }$, *and* ${{\,\mathrm{\mathbb {H}}\,}}_{M_n\epsilon _n} ({{\,\mathrm{\nu (\boldsymbol{\theta }_0)}\,}})$ *be the Hellinger neighborhood of radius* $M_n\epsilon _n$ *around the truth* ${{\,\mathrm{\nu (\boldsymbol{\theta }_0)}\,}}$. *Suppose Lemmas* 2.2, 2.3*and* 2.4*holds for sequence* $\epsilon _n = \sqrt{\left( \sum _{l=0}^L r_l + \xi \right) \sum _{l=0}^L u_l}$, *with constants* $r_l$ *and* $u_l$ *depend on* *n*. *Then for some slowly increasing sequence* $M_n \rightarrow \infty$, $M_n\epsilon _n \rightarrow 0$, *we have*

$$\begin{aligned} q^\star \left( {{\,\mathrm{\mathbb {H}}\,}}_{M_n\epsilon _n}^\complement \right) \buildrel d \over \rightarrow 0, \quad n \rightarrow \infty . \end{aligned}$$

This result leverages variational posterior approximations to establish consistency, which is non-trivial and highlights the strength of the wsBNN framework in balancing computational tractability and theoretical guarantees. The proof strategy appears to align with techniques from Bayesian asymptotics, where posterior consistency is established via prior mass, test function construction, and neighborhood-specific likelihood control (e.g., [38]) in nonparametric settings.

Lemmas 2.1–2.4 and Theorem 2.5 are established to guarantee posterior consistency of wsBNN under spike-and-slab priors for weights with shared parameters. While the proofs focus on asymptotic properties, the covering number bounds have a direct practical implication for convergence behavior. The bounds limit the model’s complexity by restricting the parameter space during posterior inference. A smaller covering number indicates lower model complexity, allowing faster convergence of the posterior to true parameter values with more data. In wsBNN, each input feature’s weights share a single spike-and-slab prior with the same set of parameters, and the network’s assumptions scale and group parameters, tighten the covering number bound, and improve convergence. This makes wsBNN a useful tool for feature selection in high-dimensional settings where posterior consistency is vital for meaningful inference.

## Results

Our experiments span both simulated datasets and popular real-world datasets from the feature selection literature. Additionally, to demonstrate the model’s applicability in practical scenarios, we present a case study on a breast cancer (BRCA) gene expression dataset from the TCGA project. To assess the effectiveness of the proposed model wsBNN in feature selection, we compare its performance against some baseline models. These include frequentist-based methods discussed, such as STG and HC, as well as the Bayesian neural network model sBNN. While sBNN has shown strong predictive performance, its use in feature selection requires post-processing to extract relevant features. For example, positive and negative weights learned while training can lead to misleading results during weight averaging, as their summation may inaccurately represent feature importance, potentially resulting in incorrect feature selection. Due to these limitations, we exclude sBNN from certain analyses focusing on direct feature selection. Additionally, for completeness, we consider two classical machine learning methods—Random Forests (RF) and Gradient Boosting (GB)—although our primary focus remains on deep learning-based nonlinear feature selection approaches.

The models wsBNN, sBNN, STG, and HC are implemented using the PyTorch framework. We use the sklearn implementation of RF and GB in our analysis. All experiments are performed on an NVIDIA DGX Server equipped with 8 NVIDIA A100 GPUs, each with 80 GB of memory. Hyperparameter tuning is performed for all models to ensure optimal performance. This involves grid search and cross-validation to identify the best combinations of learning rates, regularization coefficients, and other model-specific parameters [35, Section S4].

### Datasets and preprocessing

This section presents an overview of synthetic, benchmark, and real-world cancer gene expression datasets used in this work and the preprocessing steps undertaken.

***Synthetic datasets*** We consider a two-class classification (regression example is in the supplement) problem, motivated by [41], where data samples $\boldsymbol{x}_i \in \mathbb {R}^d, y_i \in \{ 0, 1 \}, i = 1, 2, \ldots$ are generated by the hierarchical model

2.1 $$\begin{aligned} \begin{aligned} y&= {\left\{ \begin{array}{ll} 1 & \text {if } \text {logistic}(f(\boldsymbol{x})) > 0.5, \\ 0 & \text {otherwise} \end{array}\right. }\\ f(\boldsymbol{x})&= 10\sin (x_1 x_2)^2 + 20 x_3^2 \quad + 10{{\,\textrm{sign}\,}}(x_4 x_5 - 0.2) + \xi , \\ x_j&\sim \text {Uniform}(0, 1), \quad \xi \sim N(0, 1) \end{aligned} \end{aligned}$$

The non-linear function $f(\boldsymbol{x})$ in (2.1) models interactions among input features while promoting sparsity. Specifically, it involves only a subset of the input *d*-dimensional features: $\boldsymbol{x}[:,0], \boldsymbol{x}[:,1], \boldsymbol{x}[:,2], \boldsymbol{x}[:,3], \boldsymbol{x}[:,4]$. This selective inclusion ensures that the output *y* is influenced solely by these five features, making the generated dataset particularly suitable for evaluating feature selection methods. We generate data points $(x_i, y_i)$, $i = 1, 2, \ldots , 5000$. From each dataset, we took $80\%$ of the samples for training and the remaining $20\%$ for testing at random. The training set is further split into a 9:1 ratio, allocating $90\%$ of the samples for training and $10\%$ for validation.

***Real-world benchmark datasets*** We use three widely used benchmark datasets commonly used in feature selection literature [14, 42].^1^ They include thousands of features and a limited number of samples. PCMAC and Basehock are derived from different categories within the 20 Newsgroups dataset.^2^ The Gisette dataset,^3^ developed as part of the NIPS 2003 feature selection challenge, focuses on handwritten digit recognition. These datasets are pre-processed and publicly available [42] (summary in Table 2).

**Table 2:** Overview of the real-world classification datasets used in the study, including the number of samples, features (for BRCA, the number of genes), classes, and dataset type. All datasets are preprocessed and contain real-valued data

Dataset	*n*	*d*	Classes	Type
BRCA	461	1519	3	RNAseq
PCMAC	1943	3289	2	Text
Gisette	7000	5000	2	Image
Basehock	1992	7862	2	Text

***TCGA BRCA gene expression dataset*** We utilize TCGA, initiated in 2006 by the National Cancer Institute and the National Human Genome Research Institute, which contains genomic data from 33 cancer types. Breast cancer is a major global health concern and one of the leading causes of cancer-related mortality among women. So, we choose the breast cancer dataset (TCGA-BRCA), which includes genomic, epigenomic, transcriptomic, and proteomic data, particularly gene expression data from the GDC Data Portal,^4^ where retrieval is based on the manifest file with the following selection criteria: Data Category - Transcriptome Profiling, Data Type - Gene Expression, Experimental Strategy - RNA-Seq, and Workflow Type - HTSeq-FPKM. Molecular subtypes annotations for each sample are acquired from cBioPortal.^5^

The BRCA dataset includes 461 samples and 20, 531 genes. Tissue samples are classified into molecular subtypes–Luminal A (LumA), Luminal B (LumB), and Basal-like (Basal). Each subtype has unique aggressiveness levels and responses to treatment, emphasizing the importance of molecular characterization in breast cancer research. We refined the data using the coefficient of variation (CV), reducing features to 1, 519.

### Model assessment under simulated conditions and sensitivity analysis

The primary objective of this section is to identify the relevant *ground truth* features while keeping high predictive performance. To assess this, we select the top 10 features using various feature selection methods and then retrain the corresponding models with these features. This process is repeated across 10 independent runs (e.g., using a random seed each run) to enhance the robustness and reliability of the results. To maintain focus in the discussion, we present the classification results in the main text. The synthetic regression experiments produced similar patterns, which are presented in the supplementary materials. Table 3 summarizes the average test accuracy, weighted precision, weighted recall, and weighted F1-score, where each row represents a model (hyperparameter and other execution configurations are in the supplement [35]), and each column denotes a different metric.

**Table 3:** Classification performance of different models on the simulated data

	Error $\hbox {analysis}^{1}$				Feature $\hbox {selection}^{2}$					
Model	Accuracy	Precision	Recall	F1	$\hbox {FC}_1$	$\hbox {FC}_2$	$\hbox {FC}_3$	$\hbox {FC}_4$	$\hbox {FC}_5$	FNR
wsBNN	.95±.06	.95±.04	.95±.06	.95±.06	7	9	10	10	10	0.08±.10
STG	.75±.09	.71±.21	.75±.09	.69±.14	0	0	10	10	10	0.40±.00
HC	.64±.01	.46±.11	.64±.01	.50±.02	0	0	0	0	0	1.00±.00
RF	.94±.01	.94±.01	.94±.01	.94±.01	10	8	10	10	10	0.04±.08
GB	.97±.00	.97±.00	.97±.00	.97±.00	10	10	10	10	10	0.00±.00

$^2$Reported as weighted Precision, Recall, and F1-score (on test sets), averages over ten runs

$^2$Feature selection Consistency (FC), False Negative Rate (FNR), over ten runs

The proposed wsBNN model is specifically designed for feature selection, leveraging a Bayesian framework with prior information and weight sharing in the first layer. Unlike sBNN, which requires post-processing (e.g., weight averaging) for feature selection, wsBNN eliminates this step through its intrinsic weight-sharing mechanism. In contrast, frequentist methods, such as STG and HC, lack prior incorporation, which limits their effectiveness.

wsBNN achieves competitive performance in both predictive accuracy and interpretability when compared with state-of-the-art baselines. In terms of test accuracy and F1-score, wsBNN achieved an average accuracy of 0.94 ± 0.06 and an F1-score of 0.94 ± 0.06, which are on par with ensemble methods such as RF and close to GB, which obtained slightly higher scores (0.97 ± 0.00). This demonstrates that wsBNN offers strong predictive capabilities while providing the flexibility of a deep learning framework to capture nonlinear feature interactions. In contrast, STG and HC displayed noticeably lower accuracies and reduced F1-scores. While HC struggles, highlighting the need for advanced architectures in high-dimensional synthetic scenarios. (See [35] for additional experiments, including linear models). wsBNN may generalize better to real-world genomic data with non-linear dependencies and high-dimensional interactions.

***Estimating feature selection consistency***

We now discuss the ability of a feature selection model to consistently identify the same or a similar set of relevant features across multiple runs of the model on a given dataset. One can study this by comparing the feature sets identified across multiple runs of a feature selection algorithm. We define a binary matrix *S* where $S_{jt}$ is 1 if $j^\text {th}$ feature is selected in $t^\text {th}$ run of the model and 0 otherwise. To quantify the occurrence of each feature, we define the consistency score $\text {FC}_j = \sum _{t=1} S_{jt}$ representing the count of occurrences of feature *j* across all model runs. Table 3 presents the feature selection consistency $\text {FC}_j$ for the first five features, with each row corresponding to a different classification model.

wsBNN displayed high stability across multiple runs, with feature consistency (FC) values reaching the maximum for $f_3$–$f_5$. This suggests that wsBNN reliably identifies the same subset of informative features, an essential property for interpretable and reproducible feature selection. In contrast, STG exhibited inconsistent selection behavior ($\hbox {FC}_1$ and $\hbox {FC}_2$ are 0) and HC showed no consistent feature selection (all FC values = 0). As expected, deterministic ensemble methods RF and GB achieved ideal consistency (all FC values = 10). We evaluate feature selection using the False Negative Rate (FNR) by checking for relevant features in the top 10. True positives (TP) are the relevant features selected, while false negatives (FN) are the unselected ones. wsBNN had a low FNR of 0.08, indicating strong positive sample identification. In contrast, STG had a higher FNR of 0.4, while RF and GB achieved perfect recall (FNR = 0.0).

Overall, the proposed wsBNN achieves competitive predictive accuracy, excellent feature selection consistency, and a low FNR, effectively balancing performance and interpretability. While ensemble methods like GB slightly outperform wsBNN in raw accuracy, wsBNN’s Bayesian inference framework makes it better suited for interpretable machine learning tasks.

***Visualizing feature importance through weight analysis, for deep learning methods wsBNN, sBNN, STG, and HC***

**Fig. 3:**
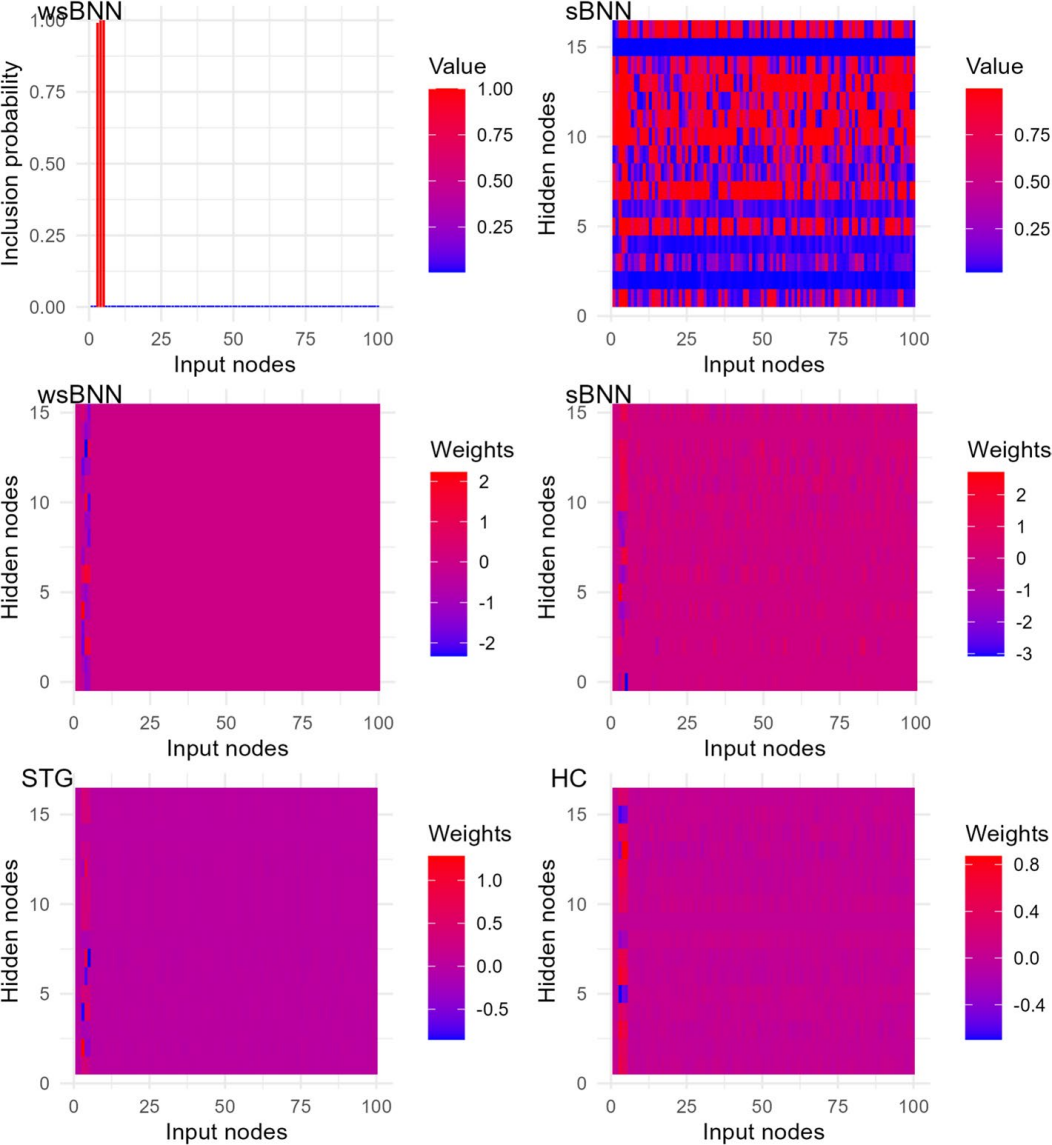
Normalized weights connecting the input layer and first hidden layer for four models on simulated data (2.1). The *x*-axis shows input nodes, while the *y*-axis indicates hidden nodes. wsBNN distinctly highlights the first five important features with clear weights. In contrast, sBNN exhibits noisy weights that need further processing for feature selection. For frequentist models, STG and HC, weights for important features vary without a clear pattern. Inclusion probabilities of weights for both wsBNN (shared inclusion probability for all weights originating from an input node) and sBNN are included for completeness

Figure 3 displays heatmaps of first-layer weights’ inclusion probabilities for wsBNN and sBNN (first row), and weights for four neural network models. This shows clear patterns in how the input features connect to the first hidden layer and how features are selected. For the weights, the *x*-axis corresponds to dataset features (input nodes), while the *y*-axis represents the hidden nodes in the first layer, with Red indicating higher weights and Blue indicating lower weights (as shown in the legend scale ranging from strong negative weights in Blue to strong positive weights in Red). The inclusion probability plots show dataset features (input nodes) on the *x*-axis and corresponding probabilities on the *y*-axis for wsBNN and the first hidden layer nodes of sBNN. Red indicates higher inclusion probability, while blue indicates lower, as per the legend. wsBNN effectively differentiates between relevant and irrelevant features by assigning significantly higher positive weights to informative variables. This clear distinction highlights its capability for precise and interpretable feature selection, unlike the more scattered weight patterns of other models.

***Analysis of sensitivity of hyperparameters and scalability of wsBNN***

We performed sensitivity analysis on two wsBNN hyperparameters: the spike-and-slab prior parameter $\lambda _0$, which governs feature inclusion, and the approximation parameter $\gamma$. Smaller $\lambda _0$ values promote sparsity by favoring the spike component, while larger values permit more features. We set $\lambda _0 =.9$ (considering a large set of features) and run the model by varying the value of temperature $\gamma \in {.1,.3,.5,.7,.9}$. Each experiment is performed for 3 independent runs and for 10000 epochs, and the results are reported in Table 4. We observed that feature selection and performance improve with higher $\gamma$, peaking at $\gamma =.7$. After keeping a constant temperature $\gamma =.7$, we varied $\lambda _0 \in {.1,.3,.5,.7,.9}$. We observed that test accuracy stabilizes around $\lambda _0 =.9$, suggesting the selection of more relevant features. For varying $\lambda _0$, little variability was seen in different runs.

**Table 4:** Sensitivity analysis of the wsBNN hyperparameters inclusion probability ($\lambda _0$) and temperature ($\gamma$)

Inclusion probability ($\lambda _0$)				Temperature ($\gamma$)			
$\lambda _0$	Accuracy	F1	FNR	$\gamma$	Accuracy	F1	FNR
.1	.622±.000	.477±.000	.40±.00	.1	.931±.033	.930±.034	.20±.16
.3	.650±.000	.512±.000	.40±.00	.3	.936±.043	.936±.043	.27±.09
.5	.661±.000	.526±.000	.26±.09	.5	.925±.036	.924±.036	.40±.00
.7	.666±.000	.533±.000	.26±.09	.7	.930±.036	.929±.036	.00±.00
.9	.952±.004	.956±.004	.26±.19	.9	.949±.014	.948±.014	.13±.19

We evaluated wsBNN on simulated classification datasets with varying feature dimensionality, $d \in {50, 100, 1000}$. For each *d*, we report test accuracy, the number of selected features, FNR, average training time per epoch, and peak memory usage, highlighting predictive performance and computational efficiency, as the feature space grows. The results indicate that wsBNN scales efficiently with comparable computational costs and memory usage as *d* increases, while maintaining competitive accuracy and consistent feature selection (Table 5). Additional results and details (with both synthetic and real data) are provided in the supplement [35].

**Table 5:** Scalability analysis of wsBNN with varying input dimensionality (*d*)

*d*	Accuracy	Features selected	FNR	Training time (s)	Memory usage (MB)
50	0.9680	4	0.20	1.7305	983
100	0.9770	5	0.00	0.5694	1100
1000	0.9700	3	0.40	0.6298	1139

***Stability analysis using Kuncheva Index*** We assessed feature selection stability using the Kuncheva Index [43, KI], which quantifies agreement between selected features over multiple runs. Higher KI values indicate better stability. wsBNN achieves high KI (0.93) for the synthetic data and competitive results for real-world datasets, often outperforming STG and matching or exceeding LASSO, RF, and GB in several cases. STG shows extreme behavior, with perfect stability in some datasets but zero in others, while HC displays dataset-dependent performance, often selecting sequential blocks of features rather than informative ones. (details in the supplement [35])

**Fig. 4:**
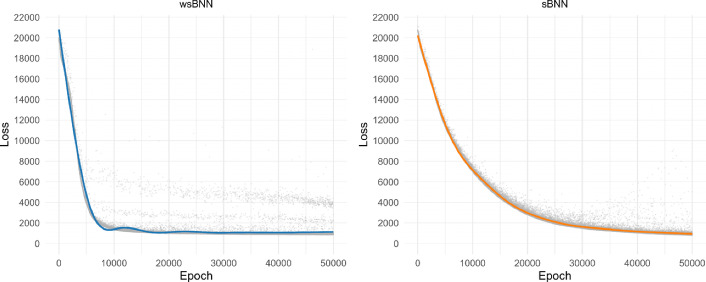
Loss progression of wsBNN and sBNN during training

***ELBO progression during training*** Fig. 4 shows the progression of the negative of ELBO for the Bayesian neural networks wsBNN and sBNN, during model training (for STG and HC, the plots in the supplement). The wsBNN loss declines sharply compared to the sBNN, decreasing rapidly in the early epochs and stabilizing after about 10, 000 iterations. Although there are minor fluctuations due to reconstruction error from stochastic sampling in the Bayesian framework, this pattern indicates that wsBNN effectively learns the underlying data distribution and converges with minimal variability. The sBNN loss exhibits slower convergence and a minor increase in loss in later epochs, indicating higher variance likely due to broader parameter space exploration without structural parameter sharing, like in wsBNN.

### Performance analysis on real-world benchmarking and gene expression datasets

**Fig. 5:**
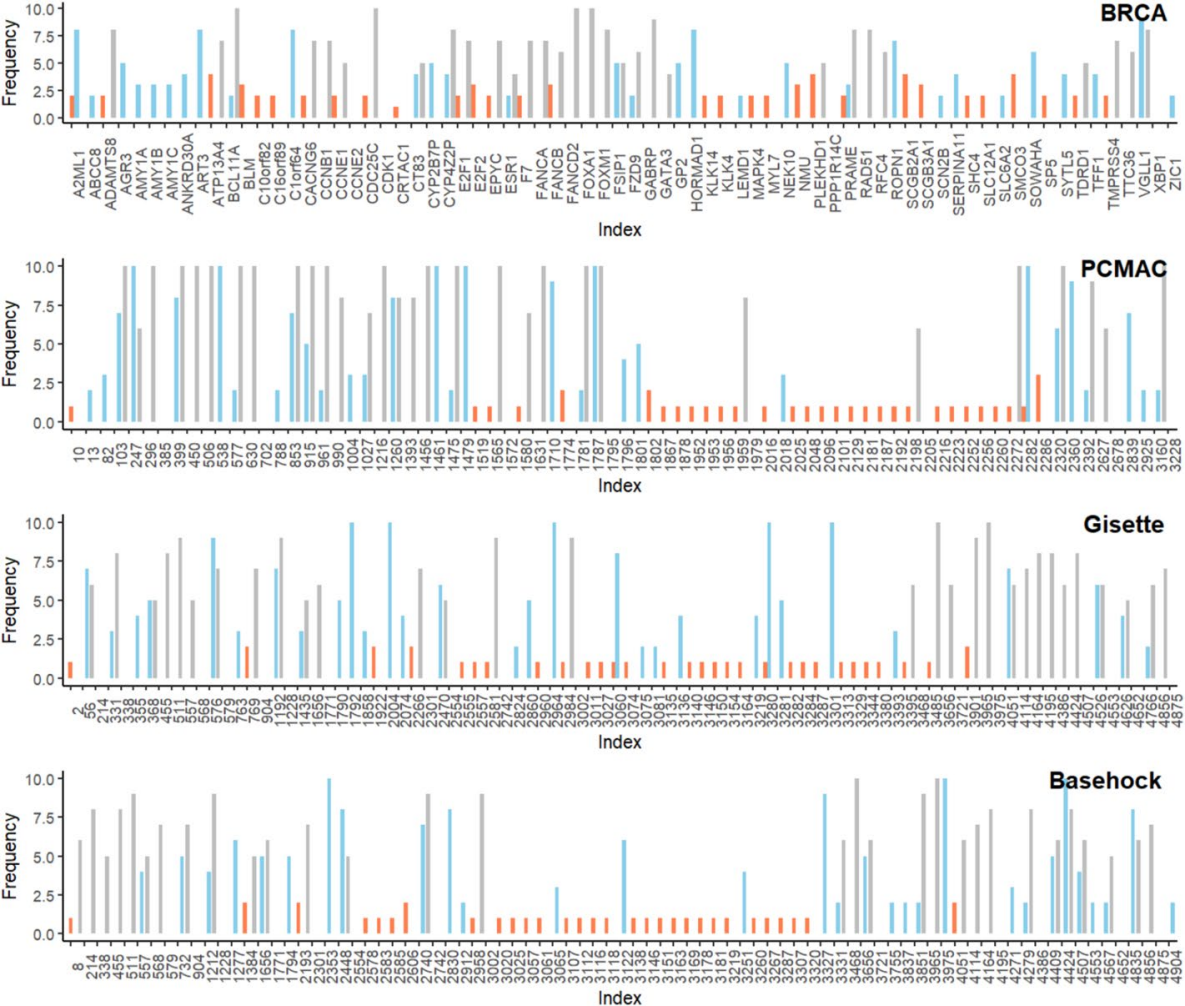
Frequency distribution of the top 30 features selected across 10 independent runs of four real-world datasets: BRCA, PCMAC, Gisette, and Basehock. The *x*-axis shows feature indices (or names), while the *y*-axis indicates the selection frequencies. Color coding – blue: wsBNN, orange: STG, gray: RF

We now compare the real data performance of wsBNN with deep learning-based and classical feature selection methods. To begin with, we study the consistency of feature selection across multiple runs, as stability is crucial to ensure reproducibility and biological interpretability. Figure 5 shows frequency plots illustrating the stability of the top 30 features selected by wsBNN, STG, and RF across four datasets. These three methods were the most effective in our predictive analysis; other approaches are excluded here for better visualization and interpretation. The *x*-axis represents the indices (or names) of the selected features, and the *y*-axis represents the frequency of the selection. Across various cancer gene expression, text, and image datasets, wsBNN consistently selects a smaller set of features while identifying a stable core subset repeatedly. In comparison, RF demonstrates a comparable pattern, with both methods showing considerable overlap in their top-ranked features, indicating agreement on key predictive signals. In contrast, STG exhibits the least consistency, showing significant variability across runs and producing a flatter frequency distribution where many features are represented only once. Further discussion of selection stability for the BRCA dataset is provided in Table 7, and additional results, including comparisons with linear methods such as LASSO, are available in the supplement [35].

**Table 6:** Weighted F1-scores of different models on four real datasets

models	wsBNN	STG	HC	RF	GB
BRCA	.7948±.05	.8558±.04	.7615±.04	.8912±.04	.9055±.03
PCMAC	.8950±.03	.4756±.03	.4913±.03	.8703±.02	.8851±.02
Gisette	.9546±.00	.5846±.09	.7025±.04	.9434±.01	.9594±.00
Basehock	.9320±.02	.5058±.03	.4976±.04	.9423±.01	.9461±.01

To evaluate the performance on real data, we select the top 30 features using various feature selection methods and train an RF classifier with each feature set. The trained classifier is then used for testing. This process is repeated across 10 independent runs to enhance the robustness and reliability of the results. Table 6 reports the weighted F1-scores, averaged over 10 runs, providing a comparative view of predictive performance across four datasets. Classical feature selection methods, RF and GB, show strong predictive performance across all datasets. Nonetheless, the proposed wsBNN shows competitive results, consistently surpassing deep learning methods STG and HC on high-dimensional datasets such as PCMAC, Gisette, and Basehock. This emphasizes its capability to capture nonlinear dependencies and select informative features effectively. For the BRCA dataset, wsBNN shows slightly lower predictive performance compared to STG, suggesting some sensitivity to dataset-specific characteristics.

Beyond its predictive accuracy, wsBNN exhibits better consistency in feature selection across different runs, which is crucial for reproducibility in high-stakes fields like biomedical research.

### Insights from gene selection: a case study on TCGA gene expression profiles

**Table 7:** Cancer-related genes selected by wsBNN, STG, and RF. Validated with the MyGene database [44]

Gene (top 1–19)	wsBNN	STG	RF	Cancer gene?	Gene (top 20–38)	wsBNN	STG	RF	Cancer gene?
HORMAD1	8	0	3	Y	AKT2	0	1	0	Y
ROPN1	7	0	6	Y	BRCA2	0	1	0	Y(B)
AGR3	5	0	8	Y(B)	EPO	0	1	0	Y
ANKRD30A	4	0	0	Y(B)	EREG	0	1	0	Y
PRAME	3	2	0	Y	HABP2	0	1	0	Y
ESR1	2	0	7	Y(B)	PAX7	0	1	0	Y
KLK4	1	2	0	Y	KLK10	0	1	0	Y(B)
GRPR	1	0	1	Y	PLA2G3	0	1	0	Y
DMBT1	1	0	0	Y	PCDH10	0	1	0	Y
NRG1	1	0	0	Y	PALB2	0	1	0	Y(B)
KCNH1	1	0	0	Y	MUC16	0	1	0	Y
RGS6	1	0	0	Y	E2F3	0	0	3	Y
MSLN	1	0	0	Y	MSH2	0	0	2	Y
AFAP1-AS1	1	0	0	Y	BRCA1	0	0	1	Y(B)
FDCSP	1	0	0	Y	MSH6	0	0	1	Y
NMU	0	3	0	Y	PROM1	0	0	1	Y
ADAMTS8	0	2	0	Y	CHEK2	0	0	1	Y
KLK14	0	2	0	Y(B)	MRAS	0	0	1	Y
ELF5	0	1	2	Y(B)	IRX1	0	0	1	Y

Top cancer-related genes selected during 10 independent runs of wsBNN and STG. Column Cancer marks cancer-associated genes: ‘Y’ and breast cancer-specific genes: ‘Y(B)’

We now evaluate the biological applicability and consistency of the proposed wsBNN model for feature selection on real-world gene expression data, using the TCGA-BRCA dataset. To assess robustness, wsBNN, STG, and RF models are executed independently across multiple runs. Features are then ranked based on their selection frequency, and the top-most frequently selected genes are further analyzed. Table 7 presents the results, structured into four columns. Column 1 lists the top 38 genes (1-19 left, 20-38 right) identified by wsBNN, STG, and RF. Column 2 reports the frequency with which each gene was selected across 10 runs of the wsBNN model, and Columns 3 and 4 show the same for the STG and RF models. Column 5 indicates the known cancer relevance of each gene to cancer, with ‘Y’ denoting general cancer genes and ‘Y(B)’ highlighting those specifically associated with breast cancer, as curated from the MyGene database [44].^6^ wsBNN demonstrates higher consistency in selecting cancer-related genes across multiple runs, making it a strong candidate for feature selection in biological datasets. On the other hand, STG shows more variability in selection, which may limit interpretability and dependability in practice. The classical RF method performs moderately, partially overlapping with wsBNN’s selected genes.

wsBNN prioritizes genes like AGR3, ANKRD30A, and ESR1, which are closely linked to breast cancer biology [45, The Human Gene Database]. This suggests that wsBNN is effective in capturing genes critical to disease-specific pathways. Similarly, wsBNN-identified genes, such as HORMAD1, NRG1, and MSLN, are implicated in essential processes like DNA repair, cell signaling, and immune response, which are central to cancer progression and treatment resistance. Genes like ROPN1, FDCSP, and KCNH1 are emerging targets [46, The Human Protein Atlas] in cancer research, demonstrating wsBNN’s ability to highlight potential biomarkers or therapeutic candidates not conventionally prioritized. Several genes, including PRAME and GRPR, have therapeutic implications, either as immune targets or in tumor microenvironment modulation. wsBNN’s focus on these genes underscores its relevance for applications in precision oncology.

Genes such as ADAMTS8 are involved in ECM remodeling, which is crucial for cancer metastasis and invasion. STG effectively highlights genes that facilitate tumor cell migration through changes in the tumor microenvironment. Genes like AKT2 and EREG are associated with critical signal transduction pathways, such as the PI3K/AKT and EGFR pathways. These pathways are heavily implicated in cell proliferation, survival, and therapeutic resistance, making these genes highly relevant for targeted therapies. Genes like BRCA2 and PCDH10 are classical tumor suppressors, emphasizing STG’s capacity to capture genes central to DNA damage repair and metastasis inhibition. Genes such as EPO and NMU highlight links between cancer and metabolic/hormonal pathways, relevant in cancers like breast cancer, where hormonal signaling plays a pivotal role. Overall, we observe that wsBNN tends to focus on breast cancer-specific genes, while STG, on the other hand, identifies genes that are broadly associated with various cancer types. This characteristic may enhance STG’s utility on datasets with various cancer subtypes. Genes like MUC16 are established cancer markers (e.g., ovarian cancer), indicating STG’s potential in diagnostic and prognostic applications.

Now we study genes ELF5, E2F3, and MSH2 that are identified by RF but not by wsBNN. ELF5 encodes an epithelium-specific ETS transcription factor involved in keratinocyte differentiation and the regulation of glandular epithelium genes (e.g., salivary gland, prostate). It also functions as a tumor-suppressive factor in breast cancer [47]. E2F3 encodes a transcription factor interacting with DP partners and pRB to regulate cell cycle genes. Amplification or overexpression of E2F3 is seen in several cancers, including aggressive breast cancer subtypes, but it is not commonly mutated in germline DNA like BRCA. MSH2 is linked to hereditary nonpolyposis colorectal cancer, but its involvement in BRCA-related carcinogenesis is unclear.

**Fig. 6:**
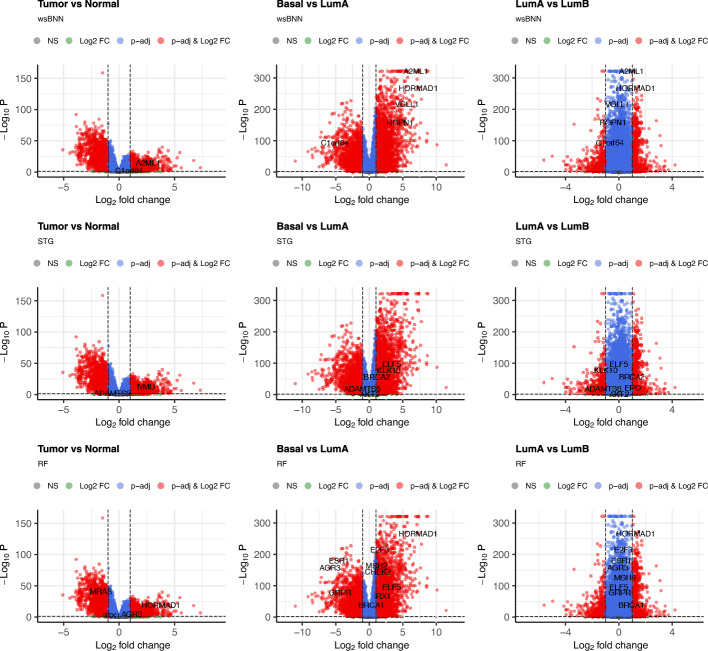
EnhancedVolcano plots of DESeq2 results for Tumor vs Normal (left), Basal vs LumA (center), and LumA vs LumB (right). Rows correspond to wsBNN-selected genes (top), STG-selected genes (middle), and RF-selected genes (bottom). Highlighted points indicate selected genes by $\log _2$ fold change and adjusted *p*-value

#### Differential expression analysis of selected genes

In this analysis, we investigate the expression patterns of breast cancer-associated genes identified through three feature selection methods—wsBNN, STG, and RF. Using gene expression data from the TCGA-BRCA cohort (RNASeq2Gene), we extracted the count matrices and clinical annotations, including tumor/normal status and PAM50 molecular subtypes. For each method, the respective selected genes were evaluated for differential expression with DESeq2, comparing (i) primary tumor versus solid tissue normal samples and (ii) molecular subtypes, such as Basal vs. LumA. Low-count genes were filtered out, and significance was determined using adjusted *p*-values and $\log _2$ fold-change thresholds.

Figure 6 shows EnhancedVolcano plots to visualize the statistical significance and the extent of expression changes, highlighting the genes selected by wsBNN, STG, and RF. This approach allows us to evaluate whether the prioritized genes by wsBNN, STG, and RF demonstrate strong and biologically relevant differential expression across clinically significant breast cancer groups. Each column corresponds to a specific comparison (e.g., Basal vs. LumA), showing how the selected genes are distributed in terms of effect size and significance.

Positive $\hbox {Log}_2$ fold change values indicate higher expression in Group 1, while negative values indicate higher expression in Group 2. Genes appear on both sides of the plot, showing that a particular gene selection method captured group-discriminating features in both directions. Some highlighted genes fall near the center with low $-\log _{10}(\text {p-adj})$ show weak differential expression evidence but may still have predictive value through subtle expression patterns. Our observations are as follows.

- Tumor vs. Normal: No significant patterns are observed, which is expected since the training dataset consisted solely of tumor samples.
- Basal vs. LumA: wsBNN-selected genes show strong discriminatory power, performing competitively with RF-selected genes. STG, however, performs less impressively in this contrast.
- LumA vs. LumB: Most genes group near the center, but wsBNN and RF identify genes with moderately high values, indicating some predictive potential. Again, STG trails behind the other two methods.

Further, enrichment analysis of selected genes using wsBNN, STG, and RF is performed with a focus on Reactome pathway analysis and Gene Ontology biological processes.

#### Pathway enrichment of selected genes

**Fig. 7:**
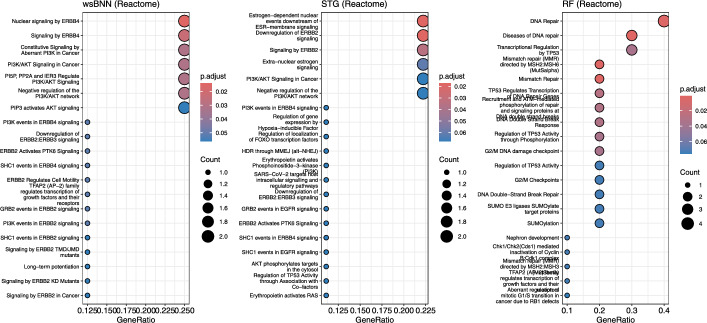
Reactome pathway enrichment analysis of selected genes by wsBNN, STG, and RF. Dot plots display the top enriched pathways (*y*-axis) along with corresponding gene ratios (*x*-axis), where the dot size indicates the number of genes and color represents the adjusted *p*-values

Reactome pathway enrichment analysis was performed on the top genes selected by wsBNN, STG, and RF (Fig. 7). Each subpanel presents a dot plot generated using the clusterProfiler and ReactomePA packages in R, summarizing the significantly enriched biological pathways for each model. The *x*-axis denotes the ratio of selected genes involved in a given pathway to the total number of genes analyzed. The *y*-axis denotes the names of significantly enriched Reactome pathways. Dot size reflects the number of contributing genes to a pathway, while color indicates adjusted *p*-value for enrichment (via Benjamini-Hochberg correction). Darker dots signify higher statistical significance.

Genes selected by wsBNN were significantly associated with ERBB2 (HER2)-mediated signaling and PI3K/AKT activation [48], hallmark oncogenic pathways in breast cancer [49]. Notably, pathways such as *Signaling by ERBB2 in Cancer*, *PI3K/AKT Signaling in Cancer*, and *TFAP2 family regulation of growth factor transcription* were among the most enriched [50], highlighting wsBNN’s ability to capture biologically coherent and clinically relevant molecular processes. Although wsBNN did not necessarily identify the ERBB2 or AKT genes themselves, its selected feature set mapped to multiple components of these pathways—indicating that wsBNN captures functionally coherent molecular processes central to HER2-driven and PI3K/AKT-mediated oncogenesis in breast cancer. In contrast, STG primarily highlighted pathways involved in DNA damage repair and p53-mediated checkpoint control [51], while the RF model emphasized estrogen receptor and growth factor-driven signaling [52]. Overall, wsBNN demonstrated superior biological interpretability by selectively identifying genes central to key HER2 and PI3K/AKT oncogenic pathways, aligning closely with known mechanisms underlying breast tumorigenesis.

#### GO enrichment of selected genes

**Fig. 8:**
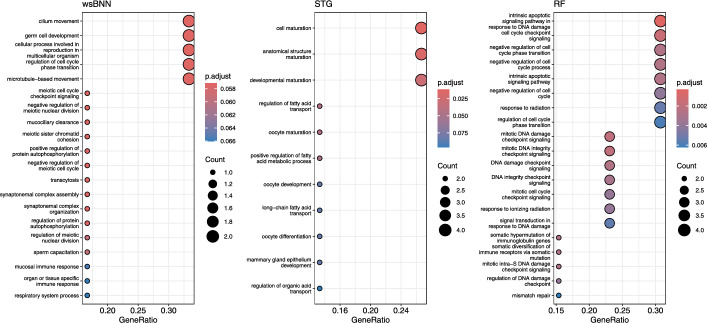
GO enrichment analysis of selected genes by wsBNN, STG, and RF using biological process terms. Dot plots display the top 20 enriched GO terms with significance and gene ratio

Gene Ontology (GO) enrichment analysis was further performed on the genes selected by wsBNN, STG, and RF to complement the Reactome pathway findings. For each method, we applied the enrichGO function (OrgDb: org.Hs.eg.db, keyType: SYMBOL) to identify significantly enriched biological processes, using the Benjamini–Hochberg method for multiple testing correction (with a *p*-value cutoff of 0.1). Figure 8 visualizes the top 20 enriched terms using dot plots to indicate their significance and gene ratio. The complete R workflow, implemented using Bioconductor packages, is available in the source code repository to ensure reproducibility.

The proposed wsBNN model displayed significant enrichment in processes such as immune response, protein autophosphorylation, and cell cycle regulation, which are central to oncogenic signaling and tumor–immune interactions in breast cancer [53]. These processes are linked to key mechanisms like receptor tyrosine kinase activation (e.g., ERBB2) and PI3K/AKT-mediated proliferation, which are critical drivers of HER2-positive breast cancer and regulators of tumor progression and therapeutic resistance [49]. These results support the capacity of wsBNN to identify biologically significant and oncogenically pertinent molecular processes.

In contrast, STG primarily enriched terms related to mammary gland epithelial development, oocyte maturation, and fatty acid metabolism, reflecting tissue-specific and developmental functions with limited direct oncogenic linkage [54]. RF demonstrated strong enrichment in DNA damage response, cell cycle checkpoint, and apoptotic signaling processes, consistent with tumor suppressor and genomic stability pathways that are frequently altered in breast cancer [51]. Collectively, the GO results support the Reactome findings, showing that wsBNN highlights genes and processes key to oncogenic signaling and immune regulation, while STG and RF focus on developmental and DNA repair biology.

## Discussion

This paper presents a weight-sharing Bayesian neural network (wsBNN) for scalable and interpretable feature selection in high-dimensional settings, such as cancer genomics. Incorporating a spike-and-slab prior for input weights with a weight-grouping with shared parameters mechanism, wsBNN encourages sparsity while reducing overfitting. Unlike many neural feature selection methods, wsBNN imposes no architectural constraints and remains computationally tractable through variational inference.

Extensive experiments on synthetic and real-world data that include TCGA-BRCA and high-dimensional real benchmark datasets demonstrate wsBNN’s ability to select consistent and biologically meaningful features. wsBNN outperforms the best completing nonlinear methods such as STG in terms of selection stability and predictive performance—wsBNN consistently prioritizes genes that are closely linked to breast cancer biology, progression, and treatment resistance. These results suggest that wsBNN is useful for applications like personalized medicine, where understanding key genomic markers can guide targeted therapies.

Integrative analyses combining differential expression and enrichment confirmed the biological relevance of wsBNN-selected genes. wsBNN identified several differentially expressed genes significantly enriched in key oncogenic processes, as revealed by both Reactome and GO enrichment analyses. Specifically, wsBNN-selected genes were involved in ERBB2 (HER2)-mediated and PI3K/AKT signaling [48], protein autophosphorylation, cell cycle regulation, and immune response [53], all of which are central to breast cancer progression and therapeutic response. In contrast, STG-selected genes primarily enriched pathways related to DNA repair and p53 checkpoint control [51], while RF-selected genes highlighted hormone receptor and growth factor-mediated signaling. These results collectively demonstrate that wsBNN effectively captures core oncogenic and immune-regulatory mechanisms underlying breast tumorigenesis, indicating its superior capability to select biologically coherent and clinically relevant genes for breast cancer prediction.

On the theoretical front, we establish key conditions for posterior consistency, including verifying the prior mass condition and identifying a KL neighborhood for the posterior. These theoretical results reinforce the reliability of the wsBNN framework for feature selection in high-dimensional data.

Nonetheless, wsBNN has certain limitations. First, the computational cost associated with variational inference in large neural networks can become significant, particularly for datasets with an extremely high number of features. Although our approach is scalable, training the model on large datasets may require considerable resources. Second, while the weight-sharing mechanism improves interpretability and reduces overfitting, it might introduce bias in cases where the assumption of uniform feature importance across hidden units is not ideal. Additionally, the performance of wsBNN is somewhat sensitive to hyperparameter settings, particularly those controlling the spike-and-slab prior, which may necessitate fine-tuning. Furthermore, the mean-field assumption in variational inference limits the model’s ability to capture parameter correlations. While weight grouping and parameter-sharing in wsBNN reduces the parameter space and improves feature selection, it may limit flexibility in situations with heterogeneous or unevenly distributed feature interactions. Nonetheless, wsBNN performs well for genomic data, as shown in this study, where correlated feature groups naturally emerge, and feature selection revealing meaningful biological structures. Future work could explore incorporating partially shared parameters to group these correlated features.

An alternative strategy for parameterizing discrete variables is to approximate Bernoulli random variables with a clipped Gaussian transformation: $z_j = \max (0, \min (1, \mu _j + \epsilon _j))$ where $\epsilon _j \sim {{\,\mathrm{\mathcal {N}}\,}}(0, \sigma ^2)$, $\mu _j$ is a learnable parameter, and $\sigma$ is a user-defined scale parameter [14]. This relaxation enables gradient-based optimization with bounded outputs but has issues like approximation bias, gradient discontinuities at clipping boundaries, and sensitivity to $\sigma$. Other methods, such as Gumbel-based reparameterization, also tackle discrete stochasticity [55]. We plan to explore these in the context of the wsBNN model in the future.

We also plan to incorporate domain knowledge, such as gene pathway annotations [56], into the Bayesian priors to enhance performance on real datasets like BRCA. Experimenting further with modified spike-and-slab priors may help better capture sparsity patterns in noisy datasets. Another direction involves optimizing the computational efficiency of variational inference to handle even larger and more complex data.

## Conclusions

This study introduces wsBNN, a novel Bayesian neural network for feature selection in high-dimensional datasets, demonstrating its effectiveness in both synthetic and real-world genomic data. By integrating a weight-grouping mechanism with a shared spike-and-slab prior for neural network weights, wsBNN balances sparsity and interpretability, while maintaining computational efficiency through variational inference. The empirical results highlight wsBNN’s advantages over existing nonlinear feature selection methods, particularly in terms of selection stability and predictive performance. These contributions have significant implications for domains such as cancer genomics, where identifying key biomarkers is essential for advancing personalized medicine. The theoretical guarantees on posterior consistency further reinforce the reliability of wsBNN for feature selection in complex, high-dimensional data settings.

## Additional file

Supplementary file 1 (pdf 3521 KB)

## Data Availability

The TCGA-BRCA gene expression dataset used in this study was obtained from the GDC Data Portal (https://portal.gdc.cancer.gov) under the TCGA-BRCA project, using RNA-Seq HTSeq-FPKM workflow. Molecular subtype annotations were retrieved from cBioPortal (https://www.cbioportal.org). The benchmark datasets used for feature selection experiments are publicly available from their respective repositories, as detailed in the manuscript and supplementary material. Synthetic datasets were generated according to the standard protocols described in the paper. All source code, preprocessing scripts, and links to the public datasets are available at https://github.com/AkankshaMishra/wsBNN. The supplementary document, containing additional experimental details, further details on the data, proofs of lemmas and theorems, and the theoretical development, is available online Mishra et al. (2026).

## Abbreviations

TCGA: The Cancer Genome Atlas
BRCA: Breast invasive carcinoma
LASSO: Least absolute shrinkage and selection operator
BNN: Bayesian neural network
wsBNN: Weight sharing Bayesian Neural Network
MCMC: Markov chain Monte Carlo
RSS: Residual sum of squares
STG: Stochastic gates
HC: Hard concrete
sBNN: Sparse Bayesian Neural Network
KL: Kullback–Leibler divergence
ELBO: Evidence lower bound

## Footnotes

1 https://jundongl.github.io/scikit-feature/datasets.html

2 http://qwone.com/~jason/20Newsgroups/

3 https://archive.ics.uci.edu/dataset/170/gisette

4 https://portal.gdc.cancer.gov

5 https://www.cbioportal.org

6 https://mygene.info
