# Supplementary material for: A weight-sharing Bayesian neural network for consistent feature selection with applications in cancer gene expression data
Source: BMC Bioinformatics. 2026 Feb 27;27:79. doi: 10.1186/s12859-026-06397-0 (PMC13041486; doi:10.1186/s12859-026-06397-0)
Supplement: Supplementary file 1 — (pdf 3521 KB) [file 12859_2026_6397_MOESM1_ESM.pdf]

Supplement to "A Weight-sharing Bayesian Neural Network for Consistent Feature Selection with Applications in Cancer Gene Expression Data"*

Akanksha Mishra[1], Wei Xia[2], and Clint Pazhayidam George[1]

[1]Indian Institute of Technology Goa, India
[2]Amazon, USA

Abstract

This document serves as supplementary material for Mishra et al. (2026). We first outline the notations and definitions relevant to our work. Next, we present the development of the variational loss function for the wsBNN model. We then provide proof of the lemmas and theorem stated in the main paper. Finally, we include additional experimental details—covering various performance metrics for linear and non-linear methods, hyperparameter tuning for deep learning models, and more details on the TCGA-BRCA dataset and preprocessing.

Note: To distinguish from the main paper, all sections, equations, figures, and tables in this document are prefixed with "S".

Contents

S1 Notations and definitions **2**

S2 The variational loss function for the wsBNN model **5**

S3 Proofs of Lemmas and Theorem 2.5 in Section 2.4 **8**
S3.1 Lemma 2.1 8
S3.2 Lemma 2.2 11
S3.3 Lemma 2.3 14
S3.4 Lemma 2.4 15
S3.5 Theorem 2.5 26
S3.6 Posterior consistency: illustrative example 28
Hellinger distance and neighborhoods 29
Simulation study 29

*Amazon had no role in this study and provided no funding or resources. This work is independent of and unrelated to Wei Xia' positions or affiliations with Amazon.

S4 Additional experimental details **30**
S4.1 Simulation studies for classification and regression 30
Classification performance and feature selection consistency 30
Regression performance and feature selection consistency 31
S4.2 Sensitivity analysis of Inclusion Probability λ_0 32
S4.3 Sensitivity analysis of Temperature γ 34
S4.4 Training loss progression 35
S4.5 Scalability study of wsBNN on real datasets 36
S4.6 Stability analysis using Kuncheva Index 37
S4.7 TCGA-BRCA data 38
S4.8 Hyperparameter tuning for deep learning models 39
S4.9 Classification performance of various feature selection models 40
S4.10Feature selection consistency on real data, for LASSO, STG, wsBNN 41

S1 Notations and definitions

This section presents a comprehensive overview of the notations and definitions used throughout this article and in the main paper. Table S1 summarizes the key notations for quick reference.

Definition 1. *Function class ($\mathcal{F}$). Let $\mathcal{X}$ and $\mathcal{Y}$ denote the input and output spaces respectively. Consider a neural network $f_{\boldsymbol{\theta},\zeta} : \mathcal{X} \to \mathcal{Y}$ with L hidden layers, their layer widths (i.e. the number of nodes in each layer) $\boldsymbol{k} = k_1, k_2, \ldots, k_L$, and activation functions of layers $\sigma_1, \sigma_2, \ldots, \sigma_L$. Here, $\boldsymbol{\theta}$ denotes learnable parameters such as weights and biases, and ζ denotes the configurable parameters such as the number of hidden layers L, layer width $\boldsymbol{k}$, layer-wise sparsity for node selection s and layer-wise norm constraints on weights B. We define the function class $\mathcal{F}_\zeta$ as:*

$$\mathcal{F}_\zeta = \{f_{\boldsymbol{\theta},\zeta} : X \to Y \,|\, \boldsymbol{\theta}, \zeta \in \Theta \times Z\},$$

where $\Theta \times Z$ is the parameter space of the neural network describing all possible network parameters. We drop the subscript ζ unless necessary to keep the notation uncluttered.

Definition 2. *Sieve. A sieve refers to a method to approximate a target function class by a sequence of simpler function classes (see, e.g., Grenander, 1981; Geman and Hwang, 1982). Let $\mathcal{F}$ be the target function class we want to approximate. We define a sequence of nested function classes $\mathcal{F}_1 \subset \mathcal{F}_2 \subset \ldots \subset \mathcal{F}_n$ with increasing flexibility or complexity, allowing it to approximate a wider range of functions. We now define a sieve, a function class, as the union of all function classes in the sequence.*

$$\mathcal{F}_{sieve} = \bigcup_{i=1}^{n} \mathcal{F}_i$$

Definition 3. *Covering Number (CN). Let $\mathcal{F}$ be a function class. and let $\|\cdot\|$ be a norm defined on the space of functions. For a given constant $\epsilon > 0$, the covering number $\mathrm{CN}(\epsilon, \mathcal{F}, \|\cdot\|)$ is the minimum number of ϵ-balls—"balls" of radius ϵ, with respect to the norm $\|\cdot\|$—needed to cover (or approximate) the entire function class $\mathcal{F}$. More formally, we write*

$$\mathrm{CN}(\epsilon, \mathcal{F}, \|\cdot\|) = \min\left\{k \in \mathbb{N} : \exists\{f_i\}_{i=1}^{k} \subset \mathcal{F} \text{ s. t. } \forall f \in \mathcal{F},\ \exists i \in \{1, 2, \ldots, k\} \text{ with } \|f - f_i\| < \epsilon\right\}$$

Table S1: Summary of the notation used in the paper

Notation	Description
n	Number of data points
d	Number of features or input dimension
k_l	Number of nodes in layer l
L	Number of hidden layers
$\mathcal{D}$	Dataset with n data points with d features
$f_0(\boldsymbol{x})$	True function of interest
$f_{\boldsymbol{\theta}}(\boldsymbol{x})$	Neural network function
$p_0(y \mid \boldsymbol{x})$	Conditional density of true model
$p_{\boldsymbol{\theta}}(y \mid \boldsymbol{x})$	Conditional density of the neural network model
$W_l \in \mathbb{R}^{k_{l-1} \times k_l}$	Weight matrix for layer l
$\boldsymbol{b}_l \in \mathbb{R}^{k_l}$	Bias vector
$\psi_l(x)$	The activation function of layer l
T	Total parameters—weight and bias—in the model
$\ell_{\mathcal{D}}(\boldsymbol{\theta}_0)$	Likelihood function of true model
$\ell_{\mathcal{D}}(\boldsymbol{\theta})$	Likelihood function of the neural network model
$\boldsymbol{z}$	Indicator variable
$\pi(\boldsymbol{\theta})$	Prior distribution
$\nu(\boldsymbol{\theta}, \boldsymbol{z} \mid \mathcal{D})$	Posterior distribution
$\pi(\boldsymbol{\theta} \mid \boldsymbol{z})$	Conditional probability density function
$m(\mathcal{D})$	Marginal density of data
$q(\boldsymbol{\theta})$	Variational posterior

where k is the minimum number of ϵ-balls needed to cover $\mathcal{F}$ and $f_1, f_2, \ldots, f_k$ are functions in $\mathcal{F}$ such that every function in $\mathcal{F}$ can be approximated by an ϵ-ball around one of these functions (Pollard, 1991).

Definition 4. Hellinger distance *(d_H). The Hellinger distance between two continuous probability distributions P and Q defined over a sample space $\mathcal{X}$ is*

$$\mathrm{d}_H(P, Q) = \sqrt{\frac{1}{2} \int \left(\sqrt{p(x)} - \sqrt{q(x)} \right)^2 dx}$$

where $p(x)$ and $q(x)$ are the probability density functions, respectively.

Definition 5. Hellinger neighborhood *($\mathbb{H}$). The Hellinger neighborhood $\mathbb{H}$ of a probability distribution $\nu(\boldsymbol{\theta}_0)$, a probability distribution parametrized by $\boldsymbol{\theta}_0$, with respect to a positive constant $\epsilon > 0$ is defined as*

$$\mathbb{H}_\epsilon(\nu(\boldsymbol{\theta}_0)) = \{\boldsymbol{\theta} : \mathrm{d}_H(\nu(\boldsymbol{\theta}), \nu(\boldsymbol{\theta}_0)) \leq \epsilon\},$$

where ϵ is a predefined threshold that determines the size of the neighborhood.

Definition 6. Kullback-Leibler (KL) divergence *(d_{KL}). The KL divergence, or relative entropy, measures how one probability distribution P is different from another probability distribution Q (P and Q are defined on the same sample space):*

$$\mathrm{d}_{KL}(P \parallel Q) = \int p(x) \log \frac{p(x)}{q(x)} dx$$

where $p(x)$ and $q(x)$ represent the probability density functions of distributions P and Q, respectively.

Definition 7. Kullback-Leibler (KL) neighborhood *($\mathbb{K}$). The KL neighborhood $\mathbb{K}_\epsilon(P(\theta_0))$ of a reference distribution $P(\theta_0)$ with respect to a positive threshold $\epsilon > 0$ is defined as*

$$\mathbb{K}_\epsilon(P(\theta_0)) = \{\boldsymbol{\theta} : \mathrm{d}_{KL}(P(\theta_0) \parallel Q(\boldsymbol{\theta})) \leq \epsilon\}$$

where $Q(\boldsymbol{\theta})$ represents another probability distribution on the sample space and ϵ is the radius of the neighborhood.

Definition 8. Bernstein Inequality. *For a sequence of independent random variables $X_1, X_2, \ldots, X_n$ with mean zero ($\mathbb{E}[X_i] = 0$) and satisfying $|X_i| \leq M$ almost surely for all i, the Bernstein inequality states that:*

$$P\left(\sum_{i=1}^{N} X_i \geq t\right) \leq \exp\left(-\frac{t^2}{2\left(\sum_{i=1}^{n} \mathrm{Var}(X_i) + \frac{M}{3}t\right)}\right)$$

where $t > 0$ is a threshold, $\mathrm{Var}(X_i)$ is the variance of the random variable X_i, and $M > 0$ is a bound on the absolute value of each random variable.

Definition 9. Hoeffding's Inequality. *Let $X_1, X_2, \ldots, X_n$ be independent, bounded random variables, i.e., $|X_i| \leq a$ and $\mathbb{E}[X_i] = 0$. Then,*

$$\mathbb{P}\left(\left|\sum_{i=1}^{n} X_i\right| > t\right) \leq 2\exp\left(-\frac{t^2}{2na^2}\right)$$

S2 The variational loss function for the wsBNN model

By the definition of KL divergence, we can write (2.15) as

$$= \underset{q_\phi(\boldsymbol{\theta},\boldsymbol{z})\in\mathcal{Q}}{\arg\min}\, \mathbb{E}_{q_\phi(\boldsymbol{\theta},\boldsymbol{z})}\left[\log\left(\frac{q_\phi(\boldsymbol{\theta},\boldsymbol{z})}{\nu(\boldsymbol{\theta},\boldsymbol{z}|\mathcal{D})}\right)\right] \tag{S2.1}$$

$$= \underset{q_\phi(\boldsymbol{\theta},\boldsymbol{z})\in\mathcal{Q}}{\arg\min}\, \mathbb{E}_{q_\phi(\boldsymbol{\theta},\boldsymbol{z})}\left[\log\left(\frac{q_\phi(\boldsymbol{\theta},\boldsymbol{z})\cdot m(\mathcal{D})}{\ell_{\mathcal{D}}(\boldsymbol{\theta})\pi(\boldsymbol{\theta},\boldsymbol{z})}\right)\right] \tag{S2.2}$$

$$= \underset{q_\phi(\boldsymbol{\theta},\boldsymbol{z})\in\mathcal{Q}}{\arg\min}\, \mathbb{E}_{q_\phi(\boldsymbol{\theta},\boldsymbol{z})}\left[\log\left(\frac{q_\phi(\boldsymbol{\theta},\boldsymbol{z})}{\pi(\boldsymbol{\theta},\boldsymbol{z})}\right) - \log\ell_{\mathcal{D}}(\boldsymbol{\theta}) + \log m(\mathcal{D})\right] \tag{S2.3}$$

$$= \underset{q_\phi(\boldsymbol{\theta},\boldsymbol{z})\in\mathcal{Q}}{\arg\min}\, \left[-\mathbb{E}_{q_\phi(\boldsymbol{\theta},\boldsymbol{z})}\left[\log\ell_{\mathcal{D}}(\boldsymbol{\theta})\right] + \mathrm{d}_{\mathrm{KL}}(q_\phi(\boldsymbol{\theta},\boldsymbol{z}) \,\|\, \pi(\boldsymbol{\theta},\boldsymbol{z}))\right] \tag{S2.4}$$

We get (S2.2) by the definition of posterior distribution. $\log m(\mathcal{D})$ is a constant with respect to $q_\phi(\boldsymbol{\theta},\boldsymbol{z})$, is ignored in the minimization, simplifying (S2.3) to (S2.4). We write the joint distribution $q_\phi(\boldsymbol{\theta},\boldsymbol{z})$ as

$$q_\phi(\boldsymbol{\theta},\boldsymbol{z}) = q_\phi(\boldsymbol{\theta}|\boldsymbol{z})q_\phi(\boldsymbol{z}), \tag{S2.5}$$

where $q_\phi(\boldsymbol{\theta}|\boldsymbol{z})$ and $q_\phi(\boldsymbol{z})$ are the conditional and the marginal distributions, respectively. The first term of (S2.4) can then be expressed as

$$\mathbb{E}_{q_\phi(\boldsymbol{\theta},\boldsymbol{z})}[\log\ell_{\mathcal{D}}(\boldsymbol{\theta})] = \mathbb{E}_{q_\phi(\boldsymbol{\theta}|\boldsymbol{z})q_\phi(\boldsymbol{z})}[\log\ell_{\mathcal{D}}(\boldsymbol{\theta})]. \tag{S2.6}$$

By the definition of the KL divergence, we expand $\mathrm{d}_{\mathrm{KL}}\left(q_\phi(\boldsymbol{\theta}, \boldsymbol{z}) \,\|\, \pi(\boldsymbol{\theta}, \boldsymbol{z})\right)$ in (S2.4) as

$$= \mathbb{E}_{q_\phi(\boldsymbol{\theta},\boldsymbol{z})}\left[\log\left(\frac{q_\phi(\boldsymbol{\theta},\boldsymbol{z})}{\pi(\boldsymbol{\theta},\boldsymbol{z})}\right)\right] \tag{S2.7}$$

$$= \mathbb{E}_{q_\phi(\boldsymbol{\theta},\boldsymbol{z})}\left[\log\left(\frac{q_\phi(\boldsymbol{\theta}|\boldsymbol{z})}{\pi(\boldsymbol{\theta}|\boldsymbol{z})} \times \frac{q_\phi(\boldsymbol{z})}{\pi(\boldsymbol{z})}\right)\right] \tag{S2.8}$$

$$= \mathbb{E}_{q_\phi(\boldsymbol{\theta},\boldsymbol{z})}\left[\log\left(\frac{q_\phi(\boldsymbol{\theta}|\boldsymbol{z})}{\pi(\boldsymbol{\theta}|\boldsymbol{z})}\right) + \log\left(\frac{q_\phi(\boldsymbol{z})}{\pi(\boldsymbol{z})}\right)\right] \tag{S2.9}$$

$$= \mathbb{E}_{q_\phi(\boldsymbol{\theta},\boldsymbol{z})}\left[\log\left(\frac{q_\phi(\boldsymbol{\theta}|\boldsymbol{z})}{\pi(\boldsymbol{\theta}|\boldsymbol{z})}\right)\right] + \mathbb{E}_{q_\phi(\boldsymbol{\theta},\boldsymbol{z})}\left[\log\left(\frac{q_\phi(\boldsymbol{z})}{\pi(\boldsymbol{z})}\right)\right] \tag{S2.10}$$

$$\begin{aligned} &= \mathbb{E}_{q_\phi(\boldsymbol{\theta},\boldsymbol{z})}\left[\log\left(\frac{\prod_{i=1}^{T_0} q_\phi(\theta_i|z_i)}{\prod_{i=1}^{T_0} \pi(\theta_i|z_i)}\right)\right] + \mathbb{E}_{q_\phi(\boldsymbol{\theta},\boldsymbol{z})}\left[\log\left(\frac{\prod_{i=1}^{T_0} q_\phi(z_i)}{\prod_{i=1}^{T_0} \pi(z_i)}\right)\right] \\ &\quad + \mathbb{E}_{q_\phi(\boldsymbol{\theta})}\left[\log\left(\frac{\prod_{i=1}^{T_r} q_\phi(\theta_i)}{\prod_{i=1}^{T_r} \pi(\theta_i)}\right)\right] \end{aligned} \tag{S2.11}$$

$$\begin{aligned} &= \mathbb{E}_{q_\phi(\boldsymbol{\theta},\boldsymbol{z})}\left[\log\left(\prod_{i=1}^{T_0} \frac{q_\phi(\theta_i|z_i)}{\pi(\theta_i|z_i)}\right)\right] + \mathbb{E}_{q_\phi(\boldsymbol{\theta},\boldsymbol{z})}\left[\log\left(\prod_{i=1}^{T_0} \frac{q_\phi(z_i)}{\pi(z_i)}\right)\right] \\ &\quad + \mathbb{E}_{q_\phi(\boldsymbol{\theta})}\left[\log\left(\prod_{i=1}^{T_r} \frac{q_\phi(\theta_i)}{\pi(\theta_i)}\right)\right] \end{aligned} \tag{S2.12}$$

$$\begin{aligned} &= \sum_{i=1}^{T_0} \mathbb{E}_{q_\phi(\boldsymbol{\theta},\boldsymbol{z})}\left[\log\left(\frac{q_\phi(\theta_i|z_i)}{\pi(\theta_i|z_i)}\right)\right] + \sum_{i=1}^{T_0} \mathbb{E}_{q_\phi(\boldsymbol{\theta},\boldsymbol{z})}\left[\log\left(\frac{q_\phi(z_i)}{\pi(z_i)}\right)\right] \\ &\quad + \sum_{i=1}^{T_r} \mathbb{E}_{q_\phi(\boldsymbol{\theta})}\left[\log\left(\frac{q_\phi(\theta_i)}{\pi(\theta_i)}\right)\right] \end{aligned} \tag{S2.13}$$

$$= \sum_{i=1}^{T_0} A_i + \sum_{i=1}^{T_0} B_i + \sum_{i=1}^{T_r} C_i \tag{S2.14}$$

We get (S2.8) by applying the chain rule of probability to decompose the joint distributions $q_\phi(\boldsymbol{\theta}, \boldsymbol{z})$ and $\pi(\boldsymbol{\theta}, \boldsymbol{z})$ into conditional and marginal probabilities. We get (S2.9) by applying logarithms of products. We obtain (S2.10) by using the linearity of expectation. By the model definition, pairs $(\theta_i, z_i),\ i = 1, 2, \ldots, T_0$, are independent and are drawn from the same distribution, satisfying the i.i.d. condition, and $\theta_1, \ldots, \theta_{T_r}$ are independent. We then write

$$q_\phi(\boldsymbol{\theta}|\boldsymbol{z}) = \prod_{i=1}^{T_0} q_\phi(\theta_i|z_i),\ q_\phi(\boldsymbol{z}) = \prod_{i=1}^{T_0} q_\phi(z_i) \qquad \pi(\boldsymbol{\theta}|\boldsymbol{z}) = \prod_{i=1}^{T_0} \pi(\theta_i|z_i),\ \pi(\boldsymbol{z}) = \prod_{i=1}^{T_0} \pi(z_i)$$

$$q_\phi(\boldsymbol{\theta}) = \prod_{i=1}^{T_r} q_\phi(\theta_i) \qquad \pi(\boldsymbol{\theta}) = \prod_{i=1}^{T_r} \pi(\theta_i)$$

We can then rewrite (S2.10) as (S2.11). For the remaining layers except for the first layer, $\boldsymbol{\theta}$ is independent of $\boldsymbol{z}$; hence, the joint distribution is simplified into marginal distribution. We obtain

(S2.12) by extracting the product terms while retaining the structure. The equation (S2.13) is obtained by applying the property of the logarithm. We denote the expectations by shorthands A_i, B_i and C_i in (S2.14). We can expand the term A_i as follows.

$$
\begin{aligned}
A_i \coloneqq\ & \mathbb{E}_{q_\phi(\theta_i, z_i)} \left[\log \left(\frac{q_\phi(\theta_i|z_i)}{\pi(\theta_i|z_i)} \right) \right] && \text{(S2.15)} \\
&= \sum_{z_i \in \{0,1\}} \int_{\theta_i} \log \left(\frac{q_\phi(\theta_i|z_i)}{\pi(\theta_i|z_i)} \right) q_\phi(\theta_i, z_i)\ d\theta_i && \text{(S2.16)} \\
&= \sum_{z_i \in \{0,1\}} \int_{\theta_i} \log \left(\frac{q_\phi(\theta_i|z_i)}{\pi(\theta_i|z_i)} \right) q_\phi(\theta_i|z_i) q_\phi(z_i)\ d\theta_i && \text{(S2.17)} \\
&= \sum_{z_i \in \{0,1\}} q_\phi(z_i) \int_{\theta_i} \log \left(\frac{q_\phi(\theta_i|z_i)}{\pi(\theta_i|z_i)} \right) q_\phi(\theta_i|z_i)\ d\theta_i && \text{(S2.18)} \\
&= \sum_{z_i \in \{0,1\}} q_\phi(z_i)\ \mathrm{d}_{\mathrm{KL}} \left(q_\phi(\theta_i|z_i) \,\|\, \pi(\theta_i|z_i) \right) && \text{(S2.19)} \\
&= q_\phi(z_i = 0)\ \mathrm{d}_{\mathrm{KL}} \left(q_\phi(\theta_i|z_i = 0) \,\|\, \pi(\theta_i|z_i = 0) \right) \\
&\quad + q_\phi(z_i = 1)\ \mathrm{d}_{\mathrm{KL}} \left(q_\phi(\theta_i|z_i = 1) \,\|\, \pi(\theta_i|z_i = 1) \right) && \text{(S2.20)} \\
&= q_\phi(z_i = 1)\ \mathrm{d}_{\mathrm{KL}} \left(\mathcal{N}(\mu_i, \tau_i^2) \,\|\, \mathcal{N}(0, \tau_0^2) \right) && \text{(S2.21)}
\end{aligned}
$$

We get (S2.16) by the defintion of expectation and (S2.17) by expanding the joint density $q_\phi(\theta_i, z_i)$. By moving the terms, $q_\phi(z_i)$'s, that are independent of the integration outside we get (S2.18). We get (S2.19) by the definition of the KL divergence and (S2.19) by expanding the sum. In the first term of (S2.20), the densities $q_\phi(\theta_i|z_i = 0)$ and $\pi(\theta_i|z_i = 0)$ correspond to the spike component, δ_{θ_i}, and thus the KL evaluates to zero. We finally get (S2.21), where we replace $q_\phi(\theta_i|z_i = 1)$ and $\pi(\theta_i|z_i = 1)$ by the corresponding densities $\mathcal{N}(\mu_i, \tau_i^2)$ and $\mathcal{N}(0, \tau_0^2)$.

We expand term B_i (S2.14) as follows

$$
\begin{aligned}
B_i \coloneqq\ & \mathbb{E}_{q_\phi(\boldsymbol{\theta}, \boldsymbol{z})} \left[\log \left(\frac{q_\phi(z_i)}{\pi(z_i)} \right) \right] && \text{(S2.22)} \\
&= \mathbb{E}_{q_\phi(z_i)} \left[\log \left(\frac{q_\phi(z_i)}{\pi(z_i)} \right) \right] \eqqcolon \mathrm{d}_{\mathrm{KL}} \left(q_\phi(z_i) \,\|\, \pi(z_i) \right) && \text{(S2.23)}
\end{aligned}
$$

In (S2.22), the log ratio of densities depends only on z_i. We thus write the expectation as the expectation with respect to the marginal $q_\phi(z_i)$ (S2.23). It is the KL divergence between two Bernoulli distributions $q_\phi(z_i)$ and $\pi(z_i)$, which we can compute in closed form. Lastly, we write term C_i (S2.14) as follows, by the definition of KL,

$$
C_i \coloneqq \mathbb{E}_{q_\phi(\boldsymbol{\theta})} \left[\log \left(\frac{q_\phi(\theta_i)}{\pi(\theta_i)} \right) \right] \eqqcolon \mathrm{d}_{\mathrm{KL}}(\mathcal{N}(\mu_i, \tau_i^2) \,\|\, \pi(0, \tau_1^2)) \qquad \text{(S2.24)}
$$

Plugging in (S2.21), (S2.23), and (S2.24) in (S2.14), we get the regularizer term

$$\begin{aligned} \mathrm{d}_{\mathrm{KL}}\left(q_\phi(\boldsymbol{\theta}, \boldsymbol{z}) \,\|\, \pi(\boldsymbol{\theta}, \boldsymbol{z})\right) = &\sum_{i=1}^{T_0} q_\phi(z_i = 1)\ \mathrm{d}_{\mathrm{KL}}\left(\mathcal{N}(\mu_i, \tau_i^2) \,\|\, \mathcal{N}(0, \tau_0^2)\right) \\ &+ \sum_{i=1}^{T_0} \mathrm{d}_{\mathrm{KL}}\left(q_\phi(z_i) \,\|\, \pi(z_i)\right) \\ &+ \sum_{i=1}^{T_r} \mathrm{d}_{\mathrm{KL}}(\mathcal{N}(\mu_i, \tau_i^2) \,\|\, \mathcal{N}(0, \tau_1^2)) \end{aligned} \tag{S2.25}$$

We get Ω (S2.26) by combining (S2.6) and (S2.25).

$$\begin{aligned} \Omega = &-\mathbb{E}_{q(\boldsymbol{\theta}|z)q(\boldsymbol{z})}\left[\log \ell_{\mathcal{D}}(\boldsymbol{\theta})\right] + \sum_{i=1}^{T_0} \mathrm{d}_{\mathrm{KL}}(q(z_i) \,\|\, \pi(z_i)) \\ &+ \sum_{i=1}^{T_0} q(z_i = 1)\, \mathrm{d}_{\mathrm{KL}}\left(\mathcal{N}(\mu_i, \tau_i^2) \,\|\, \mathcal{N}(0, \tau_0^2)\right) \\ &+ \sum_{i=1}^{T_r} \mathrm{d}_{\mathrm{KL}}\left(\mathcal{N}(\mu_i, \tau_i^2) \,\|\, \mathcal{N}(0, \tau_1^2)\right), \end{aligned} \tag{S2.26}$$

Here, the closed-form expressions for the KL divergence between two Bernoulli distributions and two Gaussian distributions are as follows:

$$\mathrm{d}_{\mathrm{KL}}\left(q_\phi(z_i) \,\|\, \pi(z_i)\right) = q_\phi(z_i) \log \frac{q_\phi(z_i)}{\pi(z_i)} + (1 - q_\phi(z_i)) \log \frac{1 - q_\phi(z_i)}{1 - \pi(z_i)} \tag{S2.27}$$

$$\mathrm{d}_{\mathrm{KL}}\left(\mathcal{N}(\mu_i, \tau_i^2) \,\|\, \mathcal{N}(0, \tau_{0,1}^2)\right) = \log \frac{\tau_{0,1}}{\tau_i} + \frac{\tau_i^2 + \mu_i^2}{2\tau_{0,1}^2} - \frac{1}{2} \tag{S2.28}$$

S3 Proofs of Lemmas and Theorem 2.5 in Section 2.4

We assume that $\boldsymbol{X} \in [0, 1]^d$, considering that we normalize the data before training.

S3.1 Lemma 2.1

Proof. of Lemma 2.1. We closely follow the framework developed in the literature, for example, see Schmidt-Hieber (2020); Jantre et al. (2023), for this proof. Recall the neural network function $f(\boldsymbol{x})$ defined in (2.3). We define two auxiliary functions: $A_l^+ f : [0, 1]^d \to \mathbb{R}^{k_l}$,

$$A_l^+ f(\boldsymbol{x}) = W_{l+1}\psi(W_l\psi(\ldots \psi(W_1\boldsymbol{x} + b_1) \ldots + b_{l-1}) + b_l) + b_{l+1}$$

and $A_l^- f : \mathbb{R}^{k_{l-1}} \to \mathbb{R}^{k_{L+1}}$,

$$A_l^- f(\boldsymbol{x}) = W_{L+1}\psi(W_L\psi(\ldots \psi(W_{l-1}\boldsymbol{x} + b_{l-1}) \ldots + b_{L-1}) + b_L) + b_{L+1}.$$

Step 1: Boundary conditions Define $A_0^+ f(\boldsymbol{x}) = \boldsymbol{x}$ and $A_{L+2}^- f(\boldsymbol{x}) = \boldsymbol{x}$. For $f \in \mathcal{F}(L, k)$, we have $|A_l^+ f(\boldsymbol{x})|_\infty \leq \prod_{l=0}^{l-1} B_l$ where $k = (d, k_1, \ldots, k_L, k_{L+1})$ and $k_{L+1} = 1$.

Step 2: Lipschitz bound on $A_l^- f$ We now derive an upper bound for the Lipschitz constant of $A_l^- f$. For any $\boldsymbol{x}_1, \boldsymbol{x}_2 \in \mathbb{R}^d$:

$$|W_L A_L^+ f(\boldsymbol{x}_1) - W_L A_L^+ f(\boldsymbol{x}_2)|_\infty = |A_l^- f(A_{l-1}^+ f(\boldsymbol{x}_1)) - A_l^- f(A_{l-1}^+ f(\boldsymbol{x}_2))|_\infty \tag{S3.1}$$

The left-hand side is bounded by

$$|W_L A_L^+ f(\boldsymbol{x}_1) - W_L A_L^+ f(\boldsymbol{x}_2)|_\infty \leq \prod_{l=0}^{L} B_l$$

The right-hand side involves the composition of Lipschitz functions $A_l^- f$ and $A_{l-1}^+ f$ with respective Lipschitz constants C_1 and C_2. Hence,

$$|A_l^- f(A_{l-1}^+ f(\boldsymbol{x}_1)) - A_l^- f(A_{l-1}^+ f(\boldsymbol{x}_2))|_\infty \leq C_1 C_2 \|\boldsymbol{x}_1 - \boldsymbol{x}_2\|_\infty \qquad \forall \boldsymbol{x}_1, \boldsymbol{x}_2 \in \mathbb{R}^d$$

Step 3: Final Bound for $\boldsymbol{x} \in [0,1]^d$. Choosing $\boldsymbol{x}_1 = \boldsymbol{x}$ and $\boldsymbol{x}_2 = 0$ yields:

$$|A_l^- f(A_{l-1}^+ f(\boldsymbol{x})) - A_l^- f(A_{l-1}^+ f(\mathbf{0})|_\infty \leq C_1 C_2 \quad \forall \boldsymbol{x} \in [0,1]^d$$

Since C_2 is Lipschitz constant for the function $A_{l-1}^+ f$ and we have the bound:

$$|A_{l-1}^+ f|_\infty \leq \prod_{l=0}^{l-2} B_l$$

It follows that

$$C_2 \leq 2 \prod_{l=0}^{l-2} B_l$$

Substituting this into the previous inequality:

$$|A_l^- f(A_{l-1}^+ f(\boldsymbol{x})) - A_l^- f(A_{l-1}^+ f(\mathbf{0})|_\infty \leq 2C_1 \prod_{l=0}^{l-2} B_l \quad \forall \boldsymbol{x} \in [0,1]^d \tag{S3.2}$$

From Equation (S3.1), the left-hand side can also be bounded by:

$$|A_l^- f(A_{l-1}^+ f(\boldsymbol{x})) - A_l^- f(A_{l-1}^+ f(\mathbf{0})|_\infty \leq 2 \prod_{j=0}^{L} B_j$$

Comparing the bounds from the previous two, we obtain

$$2C_1 \prod_{l=0}^{l-2} B_l \leq 2 \prod_{l=0}^{L} B_l$$

Dividing both sides by $2\prod_{i=0}^{l-2} B_i$ gives:

$$C_1 \le \prod_{j=l-1}^{L} B_j$$

This provides the required bound on the Lipschitz constant C_1.

Let f and f^* be two neural networks. We define $\overline{\delta}_l$ using L_1 norms of the rows of $\overline{D}_l = \overline{W}_l - \overline{W}_l^*$ as follows: $\overline{\delta}_l = (\|\overline{d}_{l1}\|_1, \dots, \|\overline{d}_{lk_{l+1}}\|_1)$.

We choose f, f^* such that $\|\overline{\delta}_l\|_\infty \le \epsilon B_l$, such that all parameters in each layer of these two networks are at most ϵB_l away from each other. Then, we can bound the absolute value of the difference by

$$\begin{aligned}
|f(\boldsymbol{x}) - f^*(\boldsymbol{x})| &\le \sum_{l=1}^{L+1} |A_{l+1}^- f(\psi(b_{l-1} + W_{l-1}A_{l-1}^+ f^*(\boldsymbol{x}))) - A_{l+1}^- f(\psi(b_{l-1}^* + W_{l-1}^* A_{l-1}^+ f^*(\boldsymbol{x})))| \\
&\le \sum_{l=1}^{L+1} \left(\prod_{j=l}^{L} B_j\right) \|\psi(b_{l-1} + W_{l-1}A_{l-1}^+ f^*(\boldsymbol{x})) - \psi(b_{l-1}^* + W_{l-1}^* A_{l-1}^+ f^*(\boldsymbol{x}))\|_\infty \\
&\le \sum_{l=1}^{L+1} \left(\prod_{j=l}^{L} B_j\right) \|b_{l-1} - b_{l-1}^* + (W_{l-1} - W_{l-1}^*)A_{l-1}^+ f^*(\boldsymbol{x})\|_\infty \\
&\le \sum_{l=1}^{L+1} \left(\prod_{j=l}^{L} B_j\right) \|\overline{\delta}_{l-1}\|_\infty \|A_{l-1}^+ f^*(\boldsymbol{x})\|_\infty \\
&\le \sum_{l=1}^{L+1} \left(\prod_{j=l}^{L} B_j\right) \epsilon B_{l-1} \prod_{j=0}^{l-2} B_j = \epsilon(L+1)\prod_{l=0}^{L} B_l
\end{aligned}$$

There is no sparsity at other layers except the first layer, the formulation of the covering number simplifies significantly because sparsity only affects the first layer's parameters. The first layer is sparse with s_0 non-zero parameters out of k_1 total parameters. There are $\binom{k_1}{s_0} \le k_1^{s_0}$ combinations to pick s_0 non-zero parameters in first layer. Since there is no sparsity in other layers, all their parameters are used in the formulation. Thus $k_l \cdot k_{l+1}$ parameters are considered without additional sparsity constraints for layer l.

We discretize non-zero parameters using equal size grid with a size

$$\frac{\delta B_l}{2(L+1)\prod_{j=0}^{L} B_j}, l = 0, \dots, L.$$

The number of possible configurations for the sparse first layer is then

$$\left(\frac{B_0}{\text{grid size}} \cdot k_1\right)^{s_0} = \left(2\delta^{-1}(L+1)\left[\prod_{j=0}^{L} B_j\right] k_1\right)^{s_0} \tag{S3.3}$$

For the remaining dense layers $l > 0$, all parameters are non-zero, so no sparsity summation is needed. The number of configurations for each dense layer l is given by

$$\left(\frac{B_l}{\text{grid size}} \cdot k_{l+1}\right)^{k_l k_{l+1}} = \left(2\delta^{-1}(L+1)\left[\prod_{j=0}^{L} B_j\right] k_{l+1}\right)^{k_l k_{l+1}} \tag{S3.4}$$

The covering number for all layers is obtained by multiplying the contributions of each layer as:

$$\mathrm{CN} \leq \left(2\delta^{-1}(L+1)\left[\prod_{j=0}^{L} B_j\right] k_1\right)^{s_0} \cdot \prod_{l=1}^{L}\left(2\delta^{-1}(L+1)\left[\prod_{j=0}^{L} B_j\right] k_{l+1}\right)^{k_l k_{l+1}}. \tag{S3.5}$$

□

S3.2 Lemma 2.2

Proof. of Lemma 2.2. We follow the framework used by Bai et al. (2020); Jantre et al. (2023) for this proof. Following (Ghosal and van der Vaart, 2007, Lemma 2), there exists a testing function $\varphi \in [0, 1]$ such that

$$\begin{aligned} \mathbb{E}_{\nu(\boldsymbol{\theta}_0)}(\varphi) &\leq \exp\left\{-n\, \mathrm{d}_{\mathrm{H}}^2(\nu(\boldsymbol{\theta}_1), \nu(\boldsymbol{\theta}_0))/2\right\} \\ \mathbb{E}_{\nu(\boldsymbol{\theta})}(1-\varphi) &\leq \exp\left\{-n\, \mathrm{d}_{\mathrm{H}}^2(\nu(\boldsymbol{\theta}_1), \nu(\boldsymbol{\theta}_0))/2\right\} \end{aligned} \tag{S3.6}$$

for all $\ell(\boldsymbol{\theta}) \in \mathcal{F}_{\zeta^\circ}$ satisfying $\mathrm{d}_{\mathrm{H}}(\nu(\boldsymbol{\theta}), \nu(\boldsymbol{\theta}_1)) \leq \mathrm{d}_{\mathrm{H}}(\nu(\boldsymbol{\theta}_0), \nu(\boldsymbol{\theta}_1))/18$.

Let $H = \mathrm{CN}(\epsilon_n/19, \mathcal{F}_{\zeta^\circ}, \mathrm{d}_{\mathrm{H}})$ denotes covering number of $\mathcal{F}_{\zeta^\circ}$. There exist H Hellinger "balls" with radius $\epsilon_n/19$ are used to cover $\mathcal{F}_{\zeta^\circ}$. For any $\nu(\boldsymbol{\theta}) \in \mathcal{F}_{\zeta^\circ}$, without loss of generality we assume $\nu(\boldsymbol{\theta})$ belongs to the Hellinger "ball" centered at $\nu(\boldsymbol{\theta}_{\mathrm{h}})$. We then have the hellinger distance $\mathrm{d}_{\mathrm{H}}(\nu(\boldsymbol{\theta}), \nu(\boldsymbol{\theta}_{\mathrm{h}})) \leq \epsilon_n/19$. If $\mathrm{d}_{\mathrm{H}}(\nu(\boldsymbol{\theta}), \nu(\boldsymbol{\theta}_0)) > \epsilon_n$, we then have that $\mathrm{d}_{\mathrm{H}}(\nu(\boldsymbol{\theta}_0), \nu(\boldsymbol{\theta}_{\mathrm{h}})) > (18/19)\epsilon_n$ using the triangle inequality:

$$\mathrm{d}_{\mathrm{H}}(\nu(\boldsymbol{\theta}), \nu(\boldsymbol{\theta}_0)) \leq \mathrm{d}_{\mathrm{H}}(\nu(\boldsymbol{\theta}), \nu(\boldsymbol{\theta}_{\mathrm{h}})) + \mathrm{d}_{\mathrm{H}}(\nu(\boldsymbol{\theta}_{\mathrm{h}}), \nu(\boldsymbol{\theta}_0))$$

Plugging in to (S3.6), we then have a testing function $\varphi \in [0, 1]$ such that

$$\mathbb{E}_{\nu(\boldsymbol{\theta}_0)}(\varphi_h) \leq \exp\left\{-\frac{n}{2}\, \mathrm{d}_{\mathrm{H}}^2(\nu(\boldsymbol{\theta}_{\mathrm{h}}), \nu(\boldsymbol{\theta}_0))\right\} \tag{S3.7}$$

$$\leq \exp\left\{-\frac{n}{2}(18^2/19^2)\epsilon_n^2\right\} \tag{S3.8}$$

$$\mathbb{E}_{\nu(\boldsymbol{\theta})}(1-\varphi_h) \leq \exp\left\{-\frac{n}{2}\, \mathrm{d}_{\mathrm{H}}^2(\nu(\boldsymbol{\theta}_{\mathrm{h}}), \nu(\boldsymbol{\theta}_0))\right\} \tag{S3.9}$$

$$\leq \exp\left\{-\frac{n}{2}(\mathrm{d}_{\mathrm{H}}(\nu(\boldsymbol{\theta}_0), \nu(\boldsymbol{\theta})) - \epsilon_n/19)^2\right\} \tag{S3.10}$$

$$\leq \exp\left\{-\frac{n}{2}(18^2/19^2)\, \mathrm{d}_{\mathrm{H}}^2(\nu(\boldsymbol{\theta}_0), \nu(\boldsymbol{\theta}))\right\} \tag{S3.11}$$

To derive (S3.11), we express the Hellinger distance in terms of $\mathrm{d}_{\mathrm{H}}(\nu(\boldsymbol{\theta}_0), \nu(\boldsymbol{\theta}))$ rather than $\mathrm{d}_{\mathrm{H}}(\nu(\boldsymbol{\theta}_{\mathrm{h}}), \nu(\boldsymbol{\theta}_0))$ by applying the triangle inequality. We then approximate the expression by focusing on the term involving $\mathrm{d}_{\mathrm{H}}(\nu(\boldsymbol{\theta}_0), \nu(\boldsymbol{\theta}))$, assuming that smaller terms $(\epsilon_n/19)^2$ contribute

insignificantly as $n \to \infty$. We use $\mathrm{d}_{\mathrm{H}}^2(\nu(\boldsymbol{\theta}_0), \nu(\boldsymbol{\theta}))(1 - \frac{2\epsilon_n}{19 \cdot \mathrm{d}_{\mathrm{H}}(\nu(\boldsymbol{\theta}_0), \nu(\boldsymbol{\theta}))})$ is lower bounded by $(18^2/19^2)\, \mathrm{d}_{\mathrm{H}}^2(\nu(\boldsymbol{\theta}_0), \nu(\boldsymbol{\theta}))$ because the exponential function $\exp(-x)$ is decreasing, a smaller value of the argument x gives a larger result.

We define a test function φ_h that is associated with a particular distribution $\nu(\boldsymbol{\theta}_{\mathrm{h}})$ and quantifies the differences between the true distribution $\nu(\boldsymbol{\theta}_0)$ and $\nu(\boldsymbol{\theta}_{\mathrm{h}})$ given the observed data. We define $\phi = \max_{h=1,\ldots,H} \varphi_h$ that represents the overall test function. We write expectation of ϕ under the true distribution $\nu(\boldsymbol{\theta}_0)$ as

$$
\begin{aligned}
\mathbb{E}_{\nu(\boldsymbol{\theta}_0)}(\phi) &= \mathbb{E}_{\nu(\boldsymbol{\theta}_0)}\left[\max_{h=1,\ldots,H} \varphi_h\right] && \text{(S3.12)} \\
&\leq \sum_h \mathbb{E}_{\nu(\boldsymbol{\theta}_0)}(\varphi_h) && \text{(S3.13)} \\
&\leq H \exp\left\{-\frac{18^2}{2 \times 19^2} n\epsilon_n^2\right\} && \text{(S3.14)} \\
&\leq \exp\left\{-\frac{18^2}{2 \times 19^2} n\epsilon_n^2 - \log H\right\} && \text{(S3.15)}
\end{aligned}
$$

We get (S3.13) by the definition of φ_h and the properties of expectation. Plugging in (S3.8) to (S3.13), we get (S3.14) and moving term H inside the exponent, we get (S3.15).

By considering $\mathcal{F}_{\zeta^\circ}$ and Lemma 2.1, we expand $\log H$ as

$$\log H = \log \mathrm{CN}(\epsilon_n, \mathcal{F}_{\zeta^\circ}, \mathrm{d_H}(.,.))$$
$$\leq \log \mathrm{CN}(\sqrt{8}\sigma_e^2\epsilon_n/19, \mathcal{F}_{\zeta^\circ}, \|.\|_\infty) \tag{S3.16}$$
$$\leq \log\left[\left(\frac{38}{\sqrt{8}\sigma_e^2\epsilon_n}(L+1)\left[\prod_{j=0}^{L} B_j\right]k_{l+1}\right)^{s_0^\circ+1}\right]$$
$$+\log\left[\prod_{l=1}^{L}\left(\frac{38}{\sqrt{8}\sigma_e^2\epsilon_n}(L+1)\left[\prod_{j=0}^{L} B_j\right]k_{l+1}\right)^{k_l\cdot k_{l+1}}\right] \tag{S3.17}$$
$$= (s_0^\circ+1)\log\left(\frac{38}{\sqrt{8}\sigma_e^2\epsilon_n}(L+1)\left[\prod_{j=0}^{L} B_j\right]k_{l+1}\right)$$
$$+\sum_{l=1}^{L} k_l\cdot k_{l+1}\log\left(\frac{38}{\sqrt{8}\sigma_e^2\epsilon_n}(L+1)\left[\prod_{j=0}^{L} B_j\right]k_{l+1}\right) \tag{S3.18}$$
$$\leq C_6(s_0^\circ+1)\left(\log\frac{1}{\epsilon_n}+\log(L+1)+\sum_{j=0}^{L}\log B_j+\log k_{l+1}\right)$$
$$+C_7\sum_{l=1}^{L} k_l\cdot k_{l+1}\left(\log\frac{1}{\epsilon_n}+\log(L+1)+\sum_{j=0}^{L}\log B_j+\log k_{l+1}\right) \tag{S3.19}$$
$$\leq C_6(s_0^\circ+1)\left(\log n+\log(L+1)+\sum_{j=0}^{L}\log B_j+\log k_{l+1}\right)$$
$$+C_7\sum_{l=1}^{L} k_l\cdot k_{l+1}\left(\log n+\log(L+1)+\sum_{j=0}^{L}\log B_j+\log k_{l+1}\right) \tag{S3.20}$$
$$\leq C_6 n\epsilon_n^2\left(\frac{(L+1)(\log n+\log(L+1))+\sum_{j=0}^{L}\log k_{j+1}+\log k_1}{(L+1)(\log n+\log(L+1)+\log k_1)}\right)$$
$$+C_7\sum_{l=1}^{L} k_l\cdot k_{l+1}\left((L+1)(\log n+\log(L+1))+\sum_{j=0}^{L}\log k_{j+1}+\log k_{l+1}\right) \tag{S3.21}$$
$$\leq C n\epsilon_n^2 \tag{S3.22}$$

The constants C_6 and C_7 are different in each step. We obtain (S3.16) by using the results $\mathrm{d}_{\mathrm{H}}^2(\nu(\boldsymbol{\theta}), \nu(\boldsymbol{\theta}_0)) \leq 1-\exp\{-\frac{1}{8\sigma_e^2}\|f_0-f_{\boldsymbol{\theta}}\|_\infty^2\}$. We get (S3.17) by applying the covering number lemma 2.1 to (S3.16). We obtain (S3.18) by using log properties. We get (S3.19) by absorbing $\log(\frac{38}{\sqrt{8}\sigma_e^2})$ to the constants. Using $\log\frac{1}{\epsilon_n} \asymp \log n$, we get (S3.20). Putting values of s_0° and B_j into (S3.20) and simplifying, we get (S3.21). The network parameters L, k_l, and k_l+1 are fixed and independent of n. The growth of each term in the inequality is either constant or logarithmic in n. Thus, the expression can be bounded by $n\epsilon_n^2$ asymptotically. Further simplifying and absorbing

constants in C, we get (S3.22). Plugging the result (S3.22) into (S3.15), we get

$$\mathbb{E}_{\nu(\boldsymbol{\theta}_0)}(\phi) \leq \sum_h \mathbb{E}_{\nu(\boldsymbol{\theta}_0)}(\varphi_h) = \exp\{-C_1 n\epsilon_n^2\}$$

for some $C_1 = (18^2/19^2)/2 - C$. On the other hand, for any $\boldsymbol{\theta}$, such that $\mathrm{d}_\mathrm{H}^2(\nu(\boldsymbol{\theta}), \nu(\boldsymbol{\theta}_0)) \geq \epsilon_n$, say $\nu(\boldsymbol{\theta})$ belongs to the hth Hellinger ball, then we have

$$\mathbb{E}_{\nu(\boldsymbol{\theta})}(1-\phi) \leq \mathbb{E}_{\nu(\boldsymbol{\theta})}(1-\varphi_h) \leq \exp\{-C_2 n\, \mathrm{d}_\mathrm{H}^2(\nu(\boldsymbol{\theta}_0), \nu(\boldsymbol{\theta}))\}$$

for some $C_2 = (18^2/19^2)/2$. This concludes the proof. □

S3.3 Lemma 2.3

Proof. of Lemma 2.3. We follow the framework used by Jantre et al. (2023) for this proof. We define

$$s_l^\circ + 1 = \frac{n\epsilon_n^2}{u_l}, \quad \frac{\lambda_0 k_1}{s_0^\circ} \to 0 \tag{S3.23}$$

We use the definition of $\mathcal{F}_\zeta$ and further combine sparsity and boundedness conditions using union bound for all layers. We write

$$\begin{aligned}
\pi(\mathcal{F}_{\zeta^\circ}^c) &\leq \pi\left(\bigcup_{l=0}^{L}\{\|\widetilde{\boldsymbol{w}}_l\|_0 > s_l^\circ\}\right) + \pi\left(\bigcup_{l=0}^{L}\{\|\widetilde{\boldsymbol{w}}_l\|_\infty > B_l^\circ\}\right) && \text{(S3.24)}\\
&\leq \sum_{l=0}^{L} \pi\left(\|\widetilde{\boldsymbol{w}}_l\|_0 > s_l^\circ\right) + \sum_{l=0}^{L} \pi\left(\|\widetilde{\boldsymbol{w}}_l\|_\infty > B_l^\circ\right) && \text{(S3.25)}\\
&= \sum_{l=0}^{L}\sum_{\boldsymbol{z}} \pi\left(\|\widetilde{\boldsymbol{w}}_l\|_0 > s_l^\circ | \boldsymbol{z}\right)\pi\left(\boldsymbol{z}\right) + \sum_{l=0}^{L}\sum_{\boldsymbol{z}} \pi\left(\|\widetilde{\boldsymbol{w}}_l\|_\infty > B_l^\circ | \boldsymbol{z}\right)\pi\left(\boldsymbol{z}\right) && \text{(S3.26)}\\
&\leq \underbrace{\sum_{l=0}^{L} \mathbb{P}\left(\sum_{i=1}^{k_{l+1}} z_{li} > s_l^\circ\right)}_{\text{Part 1}} + \underbrace{\sum_{l=0}^{L} \mathbb{P}\left(\sup_{i=1,\ldots,k_{l+1}} \|w_{li}\|_1 > B_l^\circ | \boldsymbol{z}\right)}_{\text{Part 2}} && \text{(S3.27)}
\end{aligned}$$

where $\widetilde{\boldsymbol{w}}_l = (\|\overline{w}_{l1}\|_1, \ldots, \|\overline{w}_{lk_{l+1}}\|_1)^T$. The prior $\widetilde{\pi}$ depends on the weight vector $\widetilde{\boldsymbol{w}}_l$ and a sparsity indicator vector $\boldsymbol{z}$ where each z_{li} indicates whether a weight w_{li} is non-zero. The prior is decomposed into likelihood term and prior on sparsity pattern using the law of total probability. In Part 1, the last inequality holds since $\pi\left(\|\widetilde{\boldsymbol{w}}_l\|_0 > s_l^\circ | \boldsymbol{z}\right) \leq 1$ (using definition of probability), $\pi(\boldsymbol{z}) \leq 1$ (ensuring prior probabilities are properly normalized) and $\pi\left(\|\widetilde{\boldsymbol{w}}_l\|_0 > s_l^\circ | \boldsymbol{z}\right) = 1$ iff $\sum z_{li} \geq s_l^\circ$. In Part 2, L_∞ norm of $\boldsymbol{w}_l$ is bounded by maximum of L_1 norm of weights, $\sup_{i=1,\ldots,k_{l+1}} \|w_{li}\|_1$.

We derive Part 1 of (S3.27): $\sum_{l=0}^{L}\mathbb{P}\left(\sum_{i=1}^{k_{l+1}} z_{li} > s_l^\circ\right)$ specific to our network as follows.

$$= \sum_{l=0}^{L}\mathbb{P}\left(\sum_{i=1}^{k_{l+1}} z_{li} - k_{l+1}\lambda_l > s_l^\circ - k_{l+1}\lambda_l\right)$$

$$\leq \sum_{l=0}^{L}\exp\left(\frac{-1/2(s_l^\circ - k_{l+1}\lambda_l)^2}{k_{l+1}\lambda_l(1-\lambda_l) + 1/3(s_l^\circ - k_{l+1}\lambda_l)}\right) \tag{S3.28}$$

$$\leq \exp\left(\frac{-1/2\left(s_0^\circ - k_1\lambda_0\right)^2}{k_1\lambda_0\left(1-\lambda_0\right) + 1/3\left(s_0^\circ - k_1\lambda_0\right)}\right) + \sum_{l=1}^{L}\exp\left(\frac{-1/2\left(k_l - k_{l+1}\right)^2}{1/3\left(k_l - k_{l+1}\right)}\right) \tag{S3.29}$$

$$\leq \exp\left(\frac{-s_0^\circ/2\left(1-\frac{k_1\lambda_0}{s_0^\circ}\right)^2}{1/3\left(1+\frac{2k_1\lambda_0}{s_0^\circ}\right)}\right) + \sum_{l=1}^{L}\exp\left(\frac{-3\left(k_l - k_{l+1}\right)}{2}\right) \tag{S3.30}$$

$$\to \exp\left(-\frac{3s_0^\circ}{2}\right) + \sum_{l=1}^{L}\exp\left(-\frac{3\left(k_l - k_{l+1}\right)}{2}\right) \tag{S3.31}$$

$$= \exp\left(-\frac{3}{2}\left(\frac{n{\epsilon_n}^2}{u_0} - 1\right)\right) + \sum_{l=1}^{L}\exp\left(-\frac{3\left(k_l - k_{l+1}\right)}{2}\right) \tag{S3.32}$$

$$\leq e^{(3/2)}\exp\left(-\frac{3n{\epsilon_n}^2}{2u_0}\right) + \sum_{l=1}^{L}\exp\left(-\frac{3\left(k_l - k_{l+1}\right)}{2}\right) \tag{S3.33}$$

$$\leq \exp\left(-\frac{3n{\epsilon_n}^2}{2u_0}\right) + \sum_{l=1}^{L}\exp\left(-\frac{3\left(k_l - k_{l+1}\right)}{2}\right) \tag{S3.34}$$

$$\leq \exp\left(-\frac{n{\epsilon_n}^2}{u_0}\right) + C \tag{S3.35}$$

We obtain (S3.28) by applying the Bernstein inequality (8) to bound the tail probability of the sum of independent Bernoulli random variables. Since there is no feature selection in other hidden layers, we use $s_l = k_l$ and $\lambda_l = 1$ for all layers except the input layer to get (S3.29). We apply assumption S3.23 to (S3.30) to get (S3.31). We put s_0° value in (S3.31) to get (S3.32). Taking out the $e^{(3/2)}$ term as in (S3.33) and ignoring it since it is a constant, we get the upper bound (S3.34). In (S3.34), we can upper bound the first part using $\exp\left(-\frac{n{\epsilon_n}^2}{u_0}\right)$ and the second part is a constant C.

Part 2 of (S3.27): $\sum_{l=0}^{L}\mathbb{P}\left(\sup_{i=1,\ldots,k_{l+1}}\|w_{li}\|_1 > B_l^\circ|\boldsymbol{z}\right)$ is bounded by $\exp\left(-n{\epsilon_n}^2\right)$ (see Jantre et al., 2023, Lemma 4.2). Thus complete the proof. □

S3.4 Lemma 2.4

Proof. of Lemma 2.4, (2.28). We adopt the framework used by Jantre et al. (2023) for the proof of KL conditions for our proposed network.

Step 1: Assumptions.

$$-\log\lambda_0 = O\left\{(k_0+1)v_0\right\}, \quad -\log(1-\lambda_0) = O\left\{\frac{s_0}{k_1}(k_0+1)v_0\right\} \tag{S3.36}$$

Step 2: Express the KL divergence in norm form.

$$\begin{aligned}
\mathrm{d}_{\mathrm{KL}}\left(\nu(\boldsymbol{\theta}_0), \nu(\boldsymbol{\theta})\right) &= \int_{\boldsymbol{x}\in[0,1]^d}\int_{y\in\mathbb{R}} \log\left(\frac{\nu(\boldsymbol{\theta}_0)\,(y,\boldsymbol{x})}{\nu(\boldsymbol{\theta})\,(y,\boldsymbol{x})}\right)\nu(\boldsymbol{\theta}_0)\,(y,\boldsymbol{x})\,dyd\boldsymbol{x} && \text{(S3.37)}\\
&= \int_{\boldsymbol{x}\in[0,1]^d}\int_{y\in\mathbb{R}} \log\left(\exp\left[-\frac{(y-f_0(\boldsymbol{x}))^2}{2}+\frac{(y-f_{\boldsymbol{\theta}}(\boldsymbol{x}))^2}{2}\right]\right)\nu(\boldsymbol{\theta}_0)\,(y,\boldsymbol{x})\,dyd\boldsymbol{x} && \text{(S3.38)}\\
&= \int_{\boldsymbol{x}\in[0,1]^d}\int_{y\in\mathbb{R}} \frac{2y(f_0(\boldsymbol{x})-f_{\boldsymbol{\theta}}(\boldsymbol{x}))-(f_0^2(\boldsymbol{x})-f_{\boldsymbol{\theta}}^2(\boldsymbol{x}))}{2}\,\nu(\boldsymbol{\theta}_0)\,(y,\boldsymbol{x})\,dyd\boldsymbol{x} && \text{(S3.39)}\\
&= \frac{1}{2}\int_{\boldsymbol{x}\in[0,1]^d}\int_{y\in\mathbb{R}} 2y(f_0(\boldsymbol{x})-f_{\boldsymbol{\theta}}(\boldsymbol{x}))\,\nu(\boldsymbol{\theta}_0)\,(y,\boldsymbol{x})\,dyd\boldsymbol{x}\\
&\quad -\frac{1}{2}\int_{\boldsymbol{x}\in[0,1]^d}\int_{y\in\mathbb{R}}(f_0^2(\boldsymbol{x})-f_{\boldsymbol{\theta}}^2(\boldsymbol{x}))\,\nu(\boldsymbol{\theta}_0)\,(y,\boldsymbol{x})\,dyd\boldsymbol{x} && \text{(S3.40)}\\
&= \int_{\boldsymbol{x}\in[0,1]^d} f_0^2(\boldsymbol{x})-f_0(x)f_{\boldsymbol{\theta}}(\boldsymbol{x})d\boldsymbol{x}-\frac{1}{2}\int_{\boldsymbol{x}\in[0,1]^d}(f_0^2(\boldsymbol{x})-f_{\boldsymbol{\theta}}^2(\boldsymbol{x}))d\boldsymbol{x} && \text{(S3.41)}\\
&= \frac{1}{2}\int_{\boldsymbol{x}\in[0,1]^d} 2(f_0^2(\boldsymbol{x})-f_0(x)f_{\boldsymbol{\theta}}(\boldsymbol{x}))-f_0^2(\boldsymbol{x})-f_{\boldsymbol{\theta}}^2(\boldsymbol{x})d\boldsymbol{x} && \text{(S3.42)}\\
&= \frac{1}{2}\int_{\boldsymbol{x}\in[0,1]^d} f_0^2(\boldsymbol{x})-2f_0(x)f_{\boldsymbol{\theta}}(\boldsymbol{x})-f_{\boldsymbol{\theta}}^2(\boldsymbol{x})d\boldsymbol{x} && \text{(S3.43)}\\
&= \frac{1}{2}\int_{\boldsymbol{x}\in[0,1]^d}(f_0(\boldsymbol{x})-f_{\boldsymbol{\theta}}(\boldsymbol{x}))^2d\boldsymbol{x} && \text{(S3.44)}\\
&= \frac{1}{2}\|f_0-f_{\boldsymbol{\theta}}\|_2^2 && \text{(S3.45)}
\end{aligned}$$

Plugging in the densities (2.2)–(2.4) to $\mathrm{d}_{\mathrm{KL}}\left(\nu(\boldsymbol{\theta}_0), \nu(\boldsymbol{\theta})\right)$, assuming $\sigma_e^2 = 1$ and $\boldsymbol{x} \in [0,1]^d$, and simplifying log ratio, expanding terms, separating the two integral terms, evaluating the inner integrals (expectation of y and normalization), simplifying the KL expression and writing in norm expression.

Step 3: Relate to L_1 norms. Let $f_{\boldsymbol{\theta}^\star}(\boldsymbol{x})$ denote the function parametrized by $\boldsymbol{\theta}^\star$ satisfying

$$\boldsymbol{\theta}^\star = \arg\min_{f_{\boldsymbol{\theta}}\in\mathcal{F}_\varsigma}\|f_{\boldsymbol{\theta}}-f_0\|_\infty^2$$

Then,

$$\|f_{\boldsymbol{\theta}^\star}-f_0\|_1 \le \|f_{\boldsymbol{\theta}^\star}-f_0\|_\infty = \sqrt{\xi} \tag{S3.46}$$

Here, we redefine $\overline{\delta_l}$ as a vector containing the L_1 norms of the rows of the matrix $\overline{D_l} = \overline{W_l} - \overline{W_l^*}$. Specifically:

$$\overline{D_l} = (\overline{d_{l1}}^T, \overline{d_{l2}}^T, \ldots, \overline{d_{lk_{l+1}}}^T)^T, \quad \text{and} \quad \overline{\delta_l} = (\|\overline{d_{l1}}\|_1, \ldots, \|\overline{d_{lk_{l+1}}}\|_1).$$

where $\|\overline{d_{li}}\|_1$ denotes the L_1 norm of the ith row of $\overline{D_l}$, calculated as the sum of the absolute value of its elements.

Step 4: Establish neighborhood bound. Next, we define a neighborhood $\mathcal{M}_{\sqrt{\sum r_l}}$ as follows:

$$\mathcal{M}_{\sqrt{\sum r_l}} = \left\{ \boldsymbol{\theta} : \|\overline{d_{li}}\|_1 \leq \frac{\sqrt{\sum r_l} B_l}{(L+1)\left(\prod_{j=0}^{L} B_j\right)}, i \in S_l, \|\overline{d_{li}}\|_1 = 0, i \in S_l^c, l = 0, \ldots, L \right\}$$

where S_l^c is the set where $\|\overline{w_{li}^*}\|_1 = 0, l = 0, \ldots, L$. Then, for every $\boldsymbol{\theta} \in \mathcal{M}_{\sqrt{\sum r_l}}$ using (16), we have

$$\|f_{\boldsymbol{\theta}} - f_{\boldsymbol{\theta}^*}\|_1 \leq \sqrt{\sum r_l} \tag{S3.47}$$

Combining (S3.46) and (S3.47), we get for $\boldsymbol{\theta} \in \mathcal{M}_{\sqrt{\sum r_l}}$,

$$\|f_{\boldsymbol{\theta}} - f_0\|_1 \leq \sqrt{\sum r_l} + \sqrt{\xi} \tag{S3.48}$$

So, we get

$$\mathrm{d}_{\mathrm{KL}}\left(\nu(\boldsymbol{\theta}_0), \nu(\boldsymbol{\theta})\right) \leq \frac{(\sqrt{\sum r_l} + \sqrt{\xi})^2}{2} \leq \sqrt{r_l} + \xi \tag{S3.49}$$

Step 5: Ensure prior mass condition. Since $\boldsymbol{\theta} \in \mathbb{K}_{\sqrt{r_l}+\xi}(\nu(\boldsymbol{\theta}_0))$ for every $\boldsymbol{\theta} \in \mathcal{M}_{\sqrt{\sum r_l}}$; therefore,

$$\int_{\boldsymbol{\theta} \in \mathbb{K}_{\sqrt{r_l}+\xi}} \pi(\boldsymbol{\theta}) d\boldsymbol{\theta} \geq \int_{\boldsymbol{\theta} \in \mathcal{M}_{\sqrt{\sum r_l}}} \pi(\boldsymbol{\theta}) d\boldsymbol{\theta} \tag{S3.50}$$

Define δ_n as the threshold for the neighborhood size and let $A = \{\overline{w_{li}} : \|\overline{w_{li}} - \overline{w_{li}^*}\|_1 \leq \delta_n\}$:

$$\delta_n = \frac{\sqrt{\sum r_l} B_l}{(L+1)\left(\prod_{j=0}^{L} B_j\right)} \tag{S3.51}$$

$$
\begin{aligned}
\pi\left(\mathcal{M}_{\sqrt{\sum r_l}}\right) &= \sum_z \pi\left(\mathcal{M}_{\sqrt{\sum r_l}}|z\right)\pi(z) && \text{(S3.52)}\\
&\geq \sum_{\{z:z_{li}=1, i\in S_l, z_{li}=0, i\in S_l^c, l=0,\ldots,L\}} \pi\left(\mathcal{M}_{\sqrt{\sum r_l}}|z\right)\pi(z) && \text{(S3.53)}\\
&= (1-\lambda_0)^{k_1-s_0}\lambda_0^{s_0}\prod_{i\in S_l}\mathbb{E}\left(1_{\{\overline{w_{li}}\in A\}}|z_{li}=1\right) && \text{(S3.54)}\\
&\geq (1-\lambda_0)^{k_1-s_0}\lambda_0^{s_0}\prod_{i\in S_l}\int_{\{\overline{w_{li}}\in A\}}\left(\frac{1}{2\pi}\right)^{\frac{k_l+1}{2}}\prod_{j=1}^{k_l+1}\exp\left(-\frac{\overline{w}_{lij}^2}{2}\right)d\overline{w}_{lij} && \text{(S3.55)}\\
&\geq (1-\lambda_0)^{k_1-s_0}\lambda_0^{s_0}\prod_{i\in S_l}\left(\frac{1}{2\pi}\right)^{\frac{k_l+1}{2}}\prod_{j=1}^{k_l+1}\int_{\overline{w}_{lij}^*-\frac{\delta_n}{k_l+1}}^{\overline{w}_{lij}^*+\frac{\delta_n}{k_l+1}}\exp\left(-\frac{\overline{w}_{lij}^2}{2}\right)d\overline{w}_{lij} && \text{(S3.56)}\\
&= (1-\lambda_0)^{k_1-s_0}\lambda_0^{s_0}\prod_{i\in S_l}\left(\frac{1}{2\pi}\right)^{\frac{k_l+1}{2}}\prod_{j=1}^{k_l+1}\frac{2\delta_n}{k_l+1}\exp\left(-\frac{\overline{w}_{lij}^2}{2}\right) && \text{(S3.57)}\\
&= (1-\lambda_0)^{k_1-s_0}\lambda_0^{s_0}\prod_{i\in S_l}\exp\left(\frac{k_l+1}{2}\log\frac{1}{2\pi}+(k_l+1)\log\frac{2\delta_n}{k_l+1}-\sum_{j=1}^{k_l+1}\frac{\widehat{w}_{lij}^2}{2}\right) && \text{(S3.58)}\\
&= \exp[-s_0\log\left(\frac{1}{\lambda_0}\right)+(k_1-s_0)\log\left(\frac{1}{1-\lambda_0}\right)\\
&\quad+\sum_{l=0}^{L}\sum_{i\in S_l}\left(-\frac{k_l+1}{2}\log\frac{1}{2\pi}-(k_l+1)\log\frac{2\delta_n}{k_l+1}+\sum_{j=1}^{k_l+1}\frac{\widehat{w}_{lij}^2}{2}\right)] && \text{(S3.59)}\\
&= \exp[-s_0\log\left(\frac{1}{\lambda_0}\right)+(k_1-s_0)\log\left(\frac{1}{1-\lambda_0}\right)-\sum_{l=0}^{L}\frac{s_l(k_l+1)}{2}\log\frac{1}{2\pi}\\
&\quad-\sum_{l=0}^{L}s_l(k_l+1)\log\frac{2\delta_n}{k_l+1}+\sum_{l=0}^{L}\sum_{i\in S_l}\sum_{j=1}^{k_l+1}\frac{\widehat{w}_{lij}^2}{2}] && \text{(S3.60)}\\
&= \exp[-s_0\log\left(\frac{1}{\lambda_0}\right)+(k_1-s_0)\log\left(\frac{1}{1-\lambda_0}\right) && \text{(S3.61)}\\
&\quad-\frac{s_0(k_0+1)}{2}\log\frac{1}{2\pi}-s_0(k_0+1)\log\frac{2\delta_n}{k_0+1}\\
&\quad-\sum_{l=1}^{L}\frac{k_l(k_l+1)}{2}\log\frac{1}{2\pi}-\sum_{l=1}^{L}k_l(k_l+1)\log\frac{2\delta_n}{k_l+1}]\cdot\exp\left[\sum_{l=0}^{L}\sum_{i\in S_l}\sum_{j=1}^{k_l+1}\frac{\widehat{w}_{lij}^2}{2}\right]
\end{aligned}
$$

where third inequality (S3.56) follows since $\mathbb{E}(1_{\{\overline{w}_{li}\in A\}}|z_{li}=0)=1$ since $\|\overline{w}_{li}^*\|_1=0$ for $i\in S_l^c$. The last inequality (S3.57) is by mean value theorem, $\widehat{w}_{lij}\in\left[\overline{w}_{lij}^*-\frac{\delta_n}{k_l+1},\overline{w}_{lij}^*+\frac{\delta_n}{k_l+1}\right]$. Now, we will break the above intermediate results (S3.61) into two parts.

Part 1. The first exponent from (S3.61).

$$s_0 \log\left(\frac{1}{\lambda_0}\right) + (k_1 - s_0)\log\left(\frac{1}{1-\lambda_0}\right) - \frac{s_0(k_0+1)}{2}\log\frac{1}{2\pi} - s_0(k_0+1)\log\frac{2\delta_n}{k_0+1}$$
$$-\sum_{l=1}^{L}\frac{k_l(k_l+1)}{2}\log\frac{1}{2\pi} - \sum_{l=1}^{L}k_l(k_l+1)\log\frac{2\delta_n}{k_l+1} \tag{S3.62}$$
$$\leq \sum_{i=0}^{L} Cnr_l + s_0(k_0+1)\log\left(\frac{2\frac{\sqrt{\sum r_l}B_l}{(L+1)(\prod_{j=0}^{L}B_j)}}{k_0+1}\right) - \sum_{l=1}^{L}k_l(k_l+1)\left(\log\frac{2\frac{\sqrt{\sum r_l}B_l}{(L+1)(\prod_{j=0}^{L}B_j)}}{k_l+1}\right) \tag{S3.63}$$
$$\leq \sum_{i=0}^{L} Cnr_l + \frac{s_0(k_0+1)}{2}\left(2\log(k_0+1) + 2\log(L+1) + 2\sum_{m=0,m\neq l}^{L}\log B_m - \log\sum r_l\right)$$
$$+\sum_{l=1}^{L}\frac{k_l(k_l+1)}{2}\left(2\log(k_0+1) + 2\log(L+1) + 2\sum_{m=0,m\neq l}^{L}\log B_m - \log\sum r_l\right) \tag{S3.64}$$
$$\leq Cn\sum r_l \tag{S3.65}$$
$$\leq n(\sum r_l + \xi) \tag{S3.66}$$

The first inequality follows from bounding using (S3.36) and expanding δ_n. The second inequality follows from simplifying the expression. The last inequality follows since $n\sum r_l \to \infty$ which implies $-\log\sum r_l = O(\log n)$.
Part 2. The second exponent from (S3.61).

$$\sum_{l=0}^{L}\sum_{i\in S_l}\sum_{j=1}^{k_l+1}\frac{\widehat{w}_{lij}^2}{2} \leq \frac{1}{2}\sum_{l=0}^{L}\sum_{i\in S_l}\sum_{j=1}^{k_l+1}\max\left(\left(\overline{w}_{lij}^* - \frac{\delta_n}{k_l+1}\right)^2, \left(\overline{w}_{lij}^* + \frac{\delta_n}{k_l+1}\right)^2\right) \tag{S3.67}$$
$$\leq \sum_{l=0}^{L}\sum_{i\in S_l}\sum_{j=1}^{k_l+1}\left({\overline{w}_{lij}^*}^2 + \frac{\delta_n^2}{(k_l+1)^2}\right) \tag{S3.68}$$
$$\leq \sum_{l=0}^{L}\sum_{i\in S_l}\|\overline{w}_{li}^*\|_1^2 + \sum_{l=0}^{L}\sum_{i\in S_l}\frac{\delta_n^2}{k_l+1} \tag{S3.69}$$
$$\leq \sum_{l=0}^{L}s_l(B_l^2+1) \tag{S3.70}$$
$$\leq n\sum r_l \tag{S3.71}$$
$$\leq n(\sum r_l + \xi) \tag{S3.72}$$

where the above line uses $\delta_n \to 0$. Finally, putting (S3.66) and (S3.72) into (S3.61) concludes the proof. □

Proof. of Lemma 2.4, (2.29). We follow the framework used by Jantre et al. (2023) for this proof.

We define

$$-\log \lambda_0 = O\{(k_0+1)\,v_0\}, -\log(1-\lambda_0) = O\{\left(\frac{s_0}{k_1}\right)(k_0+1)\,v_0\} \tag{S3.73}$$

Suppose there exists $q \in \mathcal{Q}^{MF}$ such that

$$\mathrm{d_{KL}}(q,\pi) \le C_1 n \sum r_l, \tag{S3.74}$$

$$\sum_{\boldsymbol{z}} \int_{\Theta} \|f_{\boldsymbol{\theta}} - f_{\boldsymbol{\theta}^\star}\|_2^2 q(\boldsymbol{\theta},\boldsymbol{z}) d\boldsymbol{\theta} \le \sum r_l \tag{S3.75}$$

Recall $\boldsymbol{\theta}^\star = \arg\min_{\boldsymbol{\theta}\in\Theta(L,p,s,B)} \|f_{\boldsymbol{\theta}} - f_0\|_\infty^2$. By relation (S3.45),

$$\sum_z \int n\, \mathrm{d_{KL}}(\nu(\boldsymbol{\theta}_0), \nu(\boldsymbol{\theta})) q(\boldsymbol{\theta},\boldsymbol{z}) d\boldsymbol{\theta} = \sum_z \frac{n}{2} \int \|f_0 - f_{\boldsymbol{\theta}}\|_2^2 q(\boldsymbol{\theta},\boldsymbol{z}) d\boldsymbol{\theta} \tag{S3.76}$$

$$\le \frac{n}{2} \sum_z \int \|f_{\boldsymbol{\theta}^\star} - f_{\boldsymbol{\theta}}\|_2^2 q(\boldsymbol{\theta},\boldsymbol{z}) d\boldsymbol{\theta} + \frac{n}{2}\|f_{\boldsymbol{\theta}^\star} - f_0\|_\infty^2 \tag{S3.77}$$

$$\le Cn(\sum r_l + \xi) \tag{S3.78}$$

where the above relation is due to (S3.75), which will complete the proof.

We next construct $q \in \mathcal{Q}^{\mathrm{MF}}$ as

$$\overline{w}_{lij} | z_{li} \sim z_{li} \mathcal{N}(\overline{w}_{lij}^*, \sigma_l^2) + (1 - z_{li})\delta_0, \qquad z_{li} \sim \mathrm{Bernoulli}(\gamma_{li}^*)$$

where $\gamma_{li}^* = 1(\|\overline{w}_{lij}^*\|_1 \neq 0)$ and $\sigma_l^2 = \frac{s_l}{8n(L+1)}(4^{L-l}(k_l+1)\log(k_{l+1}2^{k_l+1})\prod_{m=0,m\neq l}^{L} B_m^2)^{-1}$. We next consider (Jantre et al., 2023, Lemma A.7).We upper bound the expectation of the supremum of the L_1 norm of multivariate Gaussian variables:

$$\int \widetilde{W}_l q(\boldsymbol{\theta},\boldsymbol{z}) d\boldsymbol{\theta} \le \int \sup_i \|\overline{w}_{li} - \overline{w}_{li}^*\|_1 q(\boldsymbol{\theta}|\boldsymbol{z}) d\boldsymbol{\theta} \le \int \sup_i \|\overline{w}_{li} - \overline{w}_{li}^*\|_1 q(\boldsymbol{\theta}|\boldsymbol{z} = \underline{1}) d\boldsymbol{\theta}$$

since $q(\boldsymbol{z}) \le 1$. If $z_{li} = 1$, then $\|\overline{w}_{li} - \overline{w}_{li}^*\|_1 = 0$, thus the above integral is maximized at $\boldsymbol{z} = 1$ where $\boldsymbol{z} = 1$ indicates all neurons are present in the network. In this case, all w_{lij} are independent Gaussian random variables. In this direction, we make use of concentration inequalities. Let $Y = \sup_i \|\overline{w}_{li} - \overline{w}_{li}^*\|_1$.

$$\exp(t\mathbb{E}Y) \le \mathbb{E}(\exp(tY)) = \mathbb{E}[\sup_i \exp(t\|\overline{w}_{li} - \overline{w}_{li}^*\|_1)] \tag{S3.79}$$

$$\le \sum_{i=1}^{k_{l+1}} \mathbb{E}[\exp(t\sum_{j=1}^{k_{l+1}} |\overline{w}_{lij} - \overline{w}_{lij}^*|)] \tag{S3.80}$$

$$= \sum_{i=1}^{k_{l+1}} \prod_{j=1}^{k_l+1} 2\exp[\frac{\sigma_l^2 t^2}{2}]\Phi(\sigma_l t) \tag{S3.81}$$

$$\le k_{l+1} 2^{k_l+1} \exp\left[(k_l+1)\frac{\sigma_l^2 t^2}{2}\right] \tag{S3.82}$$

Thus,

$$EY \leq \frac{\log(k_{l+1}2^{k+l+1}) + (k_l+1)\frac{\sigma_l^2 t^2}{2}}{t}. \tag{S3.83}$$

Putting $t = \frac{1}{\sigma_l}\sqrt{\frac{2}{(k_l+1)}\log(k_{l+1}2^{k+l+1})}$ in above equation,

$$EY \leq \sigma_l \sqrt{\frac{k_l+1}{2}}\left[\sqrt{\log(k_{l+1}2^{k+l+1})} + \sqrt{\log(k_{l+1}2^{k+l+1})}\right] \tag{S3.84}$$

$$= \sqrt{2\sigma_l^2(k_l+1)\log(k_{l+1}2^{k+l+1})} \tag{S3.85}$$

$$\leq \sqrt{4\sigma_l^2(k_l+1)\log(k_{l+1}2^{k+l+1})} \tag{S3.86}$$

Similarly,

$$\int \widetilde{W}_l^2 q(\boldsymbol{\theta}, z)d\boldsymbol{\theta} = \int \sup_i(\|\overline{w}_{li} - \overline{w}_{li}^*\|_1)^2 q(\boldsymbol{\theta}, z)d\boldsymbol{\theta} \leq \int \sup_i(\|\overline{w}_{li} - \overline{w}_{li}^*\|_1)^2 q(\boldsymbol{\theta}, z=1)$$

Let $Y' = \sup_i(\|\overline{w}_{li} - \overline{w}_{li}^*\|_1)^2$.

$$\exp(t\mathbb{E}Y') \leq \mathbb{E}(\exp(tY')) = \mathbb{E}[\sup_i \exp(t(\|\overline{w}_{li} - \overline{w}_{li}^*\|_1)^2)] \tag{S3.87}$$

$$\leq \sum_{i=1}^{k_{l+1}} \mathbb{E}[\exp(t(\sum_{j=1}^{k_l+1}|\overline{w}_{lij} - \overline{w}_{lij}^*|)^2)] \tag{S3.88}$$

$$\leq \sum_{i=1}^{k_{l+1}} \mathbb{E}[\exp(t(k_l+1)\sum_{j=1}^{k_l+1}(\overline{w}_{lij} - \overline{w}_{lij}^*)^2)] \tag{S3.89}$$

$$= \sum_{i=1}^{k_{l+1}}\prod_{j=1}^{k_l+1} \mathbb{E}[\exp(t(k_l+1)(\overline{w}_{lij} - \overline{w}_{lij}^*)^2)] \tag{S3.90}$$

$$= \sum_{i=1}^{k_{l+1}}\prod_{j=1}^{k_l+1}\left(\frac{1}{1-2t(k_l+1)\sigma_l^2}\right)^{\frac{1}{2}} \tag{S3.91}$$

$$\leq k_{l+1}\left(\frac{1}{1-2t(k_l+1)\sigma_l^2}\right)^{\frac{(k_l+1)}{2}} \tag{S3.92}$$

Thus,

$$EY' \leq \frac{\log k_{l+1} - \left(\frac{k_l+1}{2}\right)\log(1-2t(k_l+1)\sigma_l^2)}{t}. \tag{S3.93}$$

Putting $t = \frac{1}{4\sigma_l^2(k_l+1)}$ in above equation,

$$EY' \leq 4\sigma_l^2(k_l+1)\left[\log k_{l+1} + \frac{k_l+1}{2}\log 2\right] \tag{S3.94}$$

$$= 4\sigma_l^2(k_l+1)\log(k_{l+1}2^{\frac{k_l+1}{2}}) \tag{S3.95}$$

$$\leq 4\sigma_l^2(k_l+1)\log(k_{l+1}2^{k_l+1}) \tag{S3.96}$$

We also get,

$$\int (\widetilde{W}_l + B_l) q(\boldsymbol{\theta}, z) d\boldsymbol{\theta} = \int \widetilde{W}_l q(\boldsymbol{\theta}, z) d\boldsymbol{\theta} + B_l \le \sqrt{4\sigma_l^2 (k_l + 1)\log(k_{l+1} 2^{k_l+1})} + B_l \le 2B_l$$

$$\begin{aligned}\int (\widetilde{W}_l + B_l)^2 q(\boldsymbol{\theta}, z) d\boldsymbol{\theta} &= \int \widetilde{W}_l^2 q(\boldsymbol{\theta}, z) d\boldsymbol{\theta} + 2B_l \int \widetilde{W}_l q(\boldsymbol{\theta}, z) d\boldsymbol{\theta} + B_l^2 \\ &\le 4\sigma_l^2 (k_l + 1)\log(k_{l+1} 2^{k_l+1}) + 2B_l \sqrt{4\sigma_l^2 (k_l + 1)\log(k_{l+1} 2^{k_l+1})} + B_l^2 \le 4B_l^2\end{aligned}$$

$$\begin{aligned}\int W_l (\widetilde{W}_l + B_l) q(\boldsymbol{\theta}, z) d\boldsymbol{\theta} &= \int \widetilde{W}_l^2 q(\boldsymbol{\theta}, z) d\boldsymbol{\theta} + B_l \widetilde{W}_l q(\boldsymbol{\theta}, z) d\boldsymbol{\theta} \\ &\le 4\sigma_l^2 (k_l + 1)\log(k_{l+1} 2^{k_l+1}) + B_l \sqrt{4\sigma_l^2 (k_l + 1)\log(k_{l+1} 2^{k_l+1})} \\ &\le \sqrt{4\sigma_l^2 (k_l + 1)\log(k_{l+1} 2^{k_l+1})} (\sqrt{4\sigma_l^2 (k_l + 1)\log(k_{l+1} 2^{k_l+1})} + B_l) \\ &\le 2B_l \sqrt{4\sigma_l^2 (k_l + 1)\log(k_{l+1} 2^{k_l+1})}\end{aligned}$$

Let $b_j = (k_j+1)\log(k_{j+1}2^{k_j+1})$. From relation (Jantre et al., 2023, Lemma A.7), we get

$$\int \|f_{\boldsymbol{\theta}} - f_{\boldsymbol{\theta}^\star}\|_2^2 q(\boldsymbol{\theta}, \boldsymbol{z}) d\boldsymbol{\theta}$$

$$\leq \sum_{j=0}^{L} c_{j-1}^2 (4\sigma_j^2 b_j) \left(\prod_{m=j+1}^{L} 4B_m^2\right)$$

$$+ 2\sum_{j=0}^{L}\sum_{j'=0}^{j-1} c_{j-1}c_{j'-1} 2B_j \sqrt{4\sigma_j^2 b_j} \left(\prod_{m=j+1}^{L} 4B_m^2\right) \sqrt{4\sigma_{j'}^2 b_{j'}} \left(\prod_{m=j'+1}^{j-1} 2B_m\right) \tag{S3.97}$$

$$= 4\sum_{j=0}^{L} 4^{L-j}\sigma_j^2 b_j \left(\prod_{m=0}^{j-1} B_m^2\right)\left(\prod_{m=j+1}^{L} B_m^2\right)$$

$$+ 8\sum_{j=0}^{L}\sum_{j'=0}^{j-1} \left(\prod_{m=0}^{j-1} B_m\right)\left(\prod_{m=0}^{j-1} B_m\right) 2B_j \left(\prod_{m=j+1}^{L} 4B_m^2\right)\left(\prod_{m=j'+1}^{j-1} 2B_m\right) \sqrt{4\sigma_j^2 b_j}\sqrt{4\sigma_{j'}^2 b_{j'}} \tag{S3.98}$$

$$= 4\sum_{j=0}^{L} 2^{2L-2j}\sigma_j^2 b_j \prod_{m=0, m\neq j}^{L} B_m^2$$

$$+ 8\sum_{j=0}^{L}\sum_{j'=0}^{j-1} 4^{L-j}2^{j-j'} \left(\prod_{m=0}^{j-1} B_m\right)\left(\prod_{m=0}^{j-1} B_m\right)\left(\prod_{m=j+1}^{L} B_m\right)\left(\prod_{m=j'+1}^{L} B_m\right) \sqrt{\sigma_j^2 b_j}\sqrt{\sigma_{j'}^2 b_{j'}} \tag{S3.99}$$

$$= 4\sum_{j=0}^{L} 2^{2L-2j}\sigma_j^2 b_j \prod_{m=0, m\neq j}^{L} B_m^2$$

$$+ 8\sum_{j=0}^{L}\sum_{j'=0}^{j-1} 4^{L-j}2^{j-j'} \left(\prod_{m=0, m\neq j}^{L} B_m\right)\left(\prod_{m=0, m\neq j}^{L} B_m\right) \sqrt{\sigma_j^2 b_j}\sqrt{\sigma_{j'}^2 b_{j'}} \tag{S3.100}$$

$$= 4\left(\sum_{j=0}^{L} 2^{L-j}\sqrt{\sigma_j^2 b_j}\left(\prod_{m=0, m\neq j}^{L} B_m\right)\right)^2 = 4\left(\sum_{j=0}^{L}\sqrt{\frac{s_j}{8n(L+1)}}\right)^2 \tag{S3.101}$$

$$= \frac{1}{2n(L+1)}\left(\sum_{j=0}^{L}\sqrt{s_j}\right)^2 \leq \frac{\sum_{j=0}^{L} s_j}{2n} \leq \sum_{j=0}^{L} r_l \tag{S3.102}$$

This concludes the proof of (S3.75). Next,

$$\mathrm{d_{KL}}(q,\pi) \le \log\frac{1}{\pi(z)} + 1(z=\gamma^*)\,\mathrm{d_{KL}}(\{\prod_{l=0}^{L-1}\prod_{i=1}^{k_{l+1}}\prod_{j=1}^{k_l+1}\{\gamma^*_{li}\mathcal{N}(\overline{w}^*_{lij},\sigma_l^2)+(1-\gamma^*_{li})\delta_0\}\prod_{j=1}^{k_L+1}\mathcal{N}(\overline{w}^*_{Lj})\},$$

$$\{\prod_{l=0}^{L-1}\prod_{i=1}^{k_{l+1}}\prod_{j=1}^{k_l+1}\{z_{li}\mathcal{N}(0,\sigma_0^2)+(1-z_{li})\delta_0\}\prod_{j=1}^{k_L+1}\mathcal{N}(0,\sigma_0^2)\}) \tag{S3.103}$$

$$= \log\frac{1}{\prod_{l=0}^{L-1}\lambda_l^{s_l}(1-\lambda_l)^{k_{l+1}-s_l}}$$

$$+ \sum_{l=0}^{L-1}\sum_{i=1}^{k_{l+1}}\sum_{j=1}^{k_l+1}\mathrm{d_{KL}}(\gamma^*_{li}\mathcal{N}(\overline{w}^*_{lij},\sigma_l^2)+(1-\gamma^*_{li})\delta_0,\gamma^*_{li}\mathcal{N}(0,\sigma_0^2)+(1-\gamma^*_{li})\delta_0)$$

$$+ \sum_{j=1}^{k_L+1}\mathrm{d_{KL}}(\mathcal{N}(\overline{w}^*_{Lj},\sigma_L^2),\mathcal{N}(0,\sigma_0^2)) \tag{S3.104}$$

$$= s_0\log\frac{1}{\lambda_0} + (k_1,s_0)\log\frac{1}{1-\lambda_0} + \sum_{l=0}^{L-1}\sum_{i=1}^{k_{l+1}}\sum_{j=1}^{k_l+1}\gamma^*_{li}\{\frac{1}{2}\log\frac{\sigma_0^2}{\sigma_l^2}+\frac{\sigma_l^2+\overline{w}^{*2}_{lij}}{2\sigma_0^2}-\frac{1}{2}\}$$

$$+ \sum_{j=1}^{k_L+1}\{\frac{1}{2}\log\frac{\sigma_0^2}{\sigma_L^2}+\frac{\sigma_L^2+\overline{w}^{*2}_{Lj}}{2\sigma_0^2}-\frac{1}{2}\} \tag{S3.105}$$

$$\le r_0 + \frac{s_0k_0+s_0}{2}\left[\frac{\sigma_0^2}{\sigma_0^2}+\frac{B_0^2}{\sigma_0^2(k_0+1)}-1+\log\frac{\sigma_0^2}{\sigma_0^2}\right]$$

$$+ \sum_{l=1}^{L-1}\frac{k_l(k_l+1)}{2}\left[\frac{\sigma_l^2}{\sigma_0^2}+\frac{B_l^2}{\sigma_0^2(k_l+1)}-1+\log\frac{\sigma_0^2}{\sigma_l^2}\right]$$

$$+ \frac{k_L+1}{2}\left[\frac{\sigma_L^2}{\sigma_0^2}+\frac{B_L^2}{\sigma_0^2(k_L+1)}-1+\log\frac{\sigma_0^2}{\sigma_L^2}\right] \tag{S3.106}$$

$$\le r_0 + \frac{s_0}{2}\frac{B_0^2}{\sigma_0^2} + \sum_{l=1}^{L-1}\frac{k_l(k_l+1)}{2}\left[\frac{\sigma_l^2}{\sigma_0^2}+\frac{B_l^2}{\sigma_0^2(k_l+1)}-1+\log\frac{\sigma_0^2}{\sigma_l^2}\right]$$

$$+ \frac{k_L+1}{2}\left[\frac{\sigma_L^2}{\sigma_0^2}+\frac{B_L^2}{\sigma_0^2(k_L+1)}-1+\log\frac{\sigma_0^2}{\sigma_L^2}\right] \tag{S3.107}$$

where the above inequality follows from (Chérief-Abdellatif and Alquier, 2018, Lemma 6.1). Let

$\sigma_0^2 = 1$, and it could be easily derived that $\sigma_l^2 \le 1$.

$$d_{\mathrm{KL}}(q,\pi) \le r_0 + \frac{s_0}{2}B_0^2 + \sum_{l=1}^{L}\frac{k_l(k_l+1)}{2}\left[\frac{B_l^2}{k_l+1} - \log\sigma_l^2\right] + \frac{k_L+1}{2}\left[\frac{B_L^2}{k_L+1} - \log\sigma_L^2\right] \tag{S3.108}$$

$$= r_0 + \frac{s_0}{2}B_0^2 + \sum_{l=1}^{L}\frac{k_l(k_l+1)}{2}\left[\frac{B_l^2}{k_l+1} - \log\left(\frac{s_l}{8n(L+1)}\left[4^{L-l}b_l\prod_{m=0,m\neq l}^{L}B_m^2\right]^{-1}\right)\right]$$
$$+ \frac{k_L+1}{2}\left[\frac{B_L^2}{k_L+1} - \log\left(\frac{1}{8n(L+1)}\left[b_L\prod_{m=0,m\neq l}^{L}B_m^2\right]^{-1}\right)\right] \tag{S3.109}$$

$$= r_0 + \frac{s_0}{2}B_0^2 + \sum_{l=1}^{L}\frac{k_l(k_l+1)}{2}\left[\frac{B_l^2}{k_l+1} - \log\left(\frac{s_l}{8n(L+1)}\left[4^{L-l}b_l\prod_{m=0,m\neq l}^{L}B_m^2\right]^{-1}\right)\right] \tag{S3.110}$$

$$= r_0 + \frac{s_0}{2}B_0^2 + \sum_{l=1}^{L}\frac{k_l}{2}B_l^2 + \sum_{l=1}^{L}\frac{k_l(k_l+1)}{2}\log\left(\frac{8n(L+1)}{s_l}\right)$$
$$+ \sum_{l=1}^{L}k_l(k_l+1)(L-l)\log 2 + \sum_{l=1}^{L}\frac{k_l(k_l+1)}{2}\log(k_l+1)$$
$$+ \sum_{l=1}^{L}\frac{k_l(k_l+1)}{2}\log\left(\log(k_{l+1}2^{k_l+1})\right) + \sum_{l=1}^{L}k_l(k_l+1)\left(\sum_{m=0,m\neq l}^{L}\log B_m\right) \tag{S3.111}$$

$$\le r_0 + \frac{s_0}{2}B_0^2 + \sum_{l=1}^{L}\frac{k_l}{2}B_l^2 + \sum_{l=1}^{L}\frac{k_l(k_l+1)}{2}\log\left(\frac{8n(L+1)}{s_l}\right)$$
$$+ L\sum_{l=1}^{L}k_l(k_l+1) + \sum_{l=1}^{L}\frac{k_l(k_l+1)}{2}\left(\log(k_l+1) + \log(k_{l+1}+k_l+1)\right)$$
$$+ \sum_{l=1}^{L}k_l(k_l+1)\left(\sum_{m=0,m\neq l}^{L}\log B_m\right) \tag{S3.112}$$

$$\le r_0 + \frac{s_0}{2}B_0^2 + \sum_{l=1}^{L}\frac{k_l}{2}B_l^2 + \sum_{l=1}^{L}\frac{k_l(k_l+1)}{2}\log\left(\frac{8n(L+1)}{s_l}\right)$$
$$+ L\sum_{l=1}^{L}k_l(k_l+1) + \sum_{l=1}^{L}\frac{k_l(k_l+1)}{2}\log(k_{l+1}+k_l+1) + \sum_{l=1}^{L}k_l(k_l+1)\left(\sum_{m=0,m\neq l}^{L}\log B_m\right) \tag{S3.113}$$

$$\le r_0 + \frac{s_0}{2}B_0^2 + \sum_{l=1}^{L}k_l(k_l+1)\Big(\frac{B_l^2}{2(k_l+1)} + \left(\sum_{m=0,m\neq l}^{L}\log B_m\right)$$
$$+ L + \log(k_{l+1}+k_l+1) + \frac{1}{2}\log\left(\frac{8n(L+1)}{s_l}\right)\Big) \tag{S3.114}$$

$$\le n\sum_{l=0}^{L}r_l \tag{S3.115}$$

This concludes the proof of (S3.74). □

S3.5 Theorem 2.5

Proof. of Theorem 2.5. This proof is adapted from (Jantre et al., 2023, Theorem 4.4) considering the proposed wsBNN model and notation used in this paper for completeness. No changes in the development. To further enunciate Lemmas 2.2 and 2.3 consider the quantity $\varepsilon_{1n} = \int_{\mathbb{H}^{\complement}_{M_n\epsilon_n}} \left(\frac{\ell(\boldsymbol{\theta})}{\ell(\boldsymbol{\theta}_0)}\right) \pi(\boldsymbol{\theta}) d\boldsymbol{\theta}$ as used in the following proof. Here, ε_{1n} can be split into two parts:

$$\varepsilon_{1n} = \int_{\mathbb{H}^{\complement}_{M_n\epsilon_n} \cap \mathcal{F}(L,k,s,B)} \left(\frac{\ell(\boldsymbol{\theta})}{\ell(\boldsymbol{\theta}_0)}\right) \pi(\boldsymbol{\theta}) d\boldsymbol{\theta} + \int_{\mathbb{H}^{\complement}_{M_n\epsilon_n} \cap \mathcal{F}(L,k,s,B)^{\complement}} \left(\frac{\ell(\boldsymbol{\theta})}{\ell(\boldsymbol{\theta}_0)}\right) \pi(\boldsymbol{\theta}) d\boldsymbol{\theta}$$

where Lemma 2.2 provides a handle on the first term by controlling the covering number of the sieve $\mathcal{F}(L, k, s, B)$ and Lemma 2.3 provide a handle on the second term by controlling $\pi(\mathcal{F}(L, k, s, B)^{\complement})$. Next, consider the quantity

$$\varepsilon_{2n} = \log \int \left(\frac{\ell(\boldsymbol{\theta})}{\ell(\boldsymbol{\theta}_0)}\right) \pi(\boldsymbol{\theta}) d\boldsymbol{\theta}$$

in the following proof. Lemma 2.4 condition 1 provides control on this term. Finally, consider the quantity

$$\varepsilon_{3n} = \mathrm{d}_{\mathrm{KL}}(q, \pi) + \sum_z \int \log \left(\frac{\ell(\boldsymbol{\theta})}{\ell(\boldsymbol{\theta}_0)}\right) q(\boldsymbol{\theta}, z) d\boldsymbol{\theta}$$

in the following proof. Lemma 2.4 condition 2 provides control on this term.

Let ν and $q^\star$ be as in (2.12) and marginal variational posterior, respectively. Now, we start with the KL divergence between the marginal posterior $q^\star$ and true posterior $\nu(|\mathcal{D})$:

$$\mathrm{d}_{\mathrm{KL}}(q^\star, \nu(|\mathcal{D})) = \int_{\mathcal{A}} q^\star(\boldsymbol{\theta}) \log \frac{q^\star(\boldsymbol{\theta})}{\nu(\boldsymbol{\theta}|\mathcal{D})} d\boldsymbol{\theta} + \int_{\mathcal{A}^{\complement}} q^\star(\boldsymbol{\theta}) \log \frac{q^\star(\boldsymbol{\theta})}{\nu(\boldsymbol{\theta}|\mathcal{D})} d\boldsymbol{\theta} \tag{S3.116}$$

$$= -q^\star(\mathcal{A}) \int_{\mathcal{A}} \frac{q^\star(\boldsymbol{\theta})}{q^\star(\mathcal{A})} \log \frac{\nu(\boldsymbol{\theta}|\mathcal{D})}{q^\star(\boldsymbol{\theta})} d\boldsymbol{\theta} - q^\star(\mathcal{A}^{\complement}) \int_{\mathcal{A}} \frac{q^\star(\boldsymbol{\theta})}{q^\star(\mathcal{A}^{\complement})} \log \frac{\nu(\boldsymbol{\theta}|\mathcal{D})}{q^\star(\boldsymbol{\theta})} d\boldsymbol{\theta} \tag{S3.117}$$

$$\geq q^\star(\mathcal{A}) \log \frac{q^\star(\mathcal{A})}{\nu(\mathcal{A}|\mathcal{D})} + q^\star(\mathcal{A}^{\complement}) \log \frac{q^\star(\mathcal{A}^{\complement})}{\nu(\mathcal{A}^{\complement}|\mathcal{D})} \tag{S3.118}$$

$$\geq q^\star(\mathcal{A}) \log q^\star(\mathcal{A}) + q^\star(\mathcal{A}^{\complement}) \log q^\star(\mathcal{A}^{\complement}) - q^\star(\mathcal{A}^{\complement}) \log \nu(\mathcal{A}^{\complement}|\mathcal{D}) \tag{S3.119}$$

$$\geq -q^\star(\mathcal{A}^{\complement}) \log \nu(\mathcal{A}^{\complement}|\mathcal{D}) - \log 2 \tag{S3.120}$$

$$= -q^\star(\mathcal{A}^{\complement}) \left(\underbrace{\log \int_{\mathcal{A}^{\complement}} (\ell(\boldsymbol{\theta}) \,/\, \ell(\boldsymbol{\theta}_0)) \nu(\boldsymbol{\theta}) d\boldsymbol{\theta}}_{\varepsilon_{1n}} - \underbrace{\log \int (\ell(\boldsymbol{\theta}) \,/\, \ell(\boldsymbol{\theta}_0)) \nu(\boldsymbol{\theta}) d\boldsymbol{\theta}}_{\varepsilon_{2n}} \right) - \log 2 \tag{S3.121}$$

where the above lines hold for any set $\mathcal{A}$. The first inequality is due to Jensen's inequality. The second inequality is because the posterior probability $\nu(\mathcal{A}|\mathcal{D}) \leq 1$. The third inequality is due to fundamental inequality in entropy bound $(x \log x + (1-x) \log(1-x) \geq -\log 2)$. In the last step,

we express posterior probability for $\mathcal{A}^{\complement}$ as $\frac{\int_{\mathcal{A}^{\complement}}(\ell(\boldsymbol{\theta})\,/\,\ell(\boldsymbol{\theta}_0))\nu(\boldsymbol{\theta})d\boldsymbol{\theta}}{\int(\ell(\boldsymbol{\theta})\,/\,\ell(\boldsymbol{\theta}_0))\nu(\boldsymbol{\theta})d\boldsymbol{\theta}}$. The above representation is similar to the proof of Theorems 3.1 and 3.2 in (Bhattacharya and Maiti, 2021). For any $q(\boldsymbol{\theta}) \in \mathcal{Q}^{\text{MF}}$,

$$
\begin{aligned}
-q^\star(\mathcal{A}^{\complement})\varepsilon_{1n} &\leq \mathrm{d}_{\mathrm{KL}}(q^\star, \nu(|\mathcal{D})) - q^\star(\mathcal{A}^{c})\varepsilon_{2n} + \log 2 && \text{(S3.122)}\\
&\leq \mathrm{d}_{\mathrm{KL}}(q^\star, \nu(|\mathcal{D})) - q^\star(\mathcal{A}^{\complement})\varepsilon_{2n} + \log 2 \quad \text{((Jantre et al., 2023, Lemma A.5))} && \text{(S3.123)}\\
&\leq \mathrm{d}_{\mathrm{KL}}(q, \nu(|\mathcal{D})) - q^\star(\mathcal{A}^{\complement})\varepsilon_{2n} + \log 2 \qquad (q^\star \text{ is KL minimizer}) && \text{(S3.124)}\\
&\leq \underbrace{\mathrm{d}_{\mathrm{KL}}(q, \nu) + \sum_{\boldsymbol{z}} \int \log \frac{\ell(\boldsymbol{\theta}_0)}{\ell(\boldsymbol{\theta})} q(\boldsymbol{\theta}, \boldsymbol{z}) d\boldsymbol{\theta}}_{\varepsilon_{3n}} + (1 - q^\star(\mathcal{A}^{\complement}))\varepsilon_{2n} + \log 2 && \text{(S3.125)}\\
&= \varepsilon_{3n} + (1 - q^\star(\mathcal{A}^{\complement}))\varepsilon_{2n} + \log 2 && \text{(S3.126)}
\end{aligned}
$$

where the last inequality in the above equation follows since

$$
\begin{aligned}
\mathrm{d}_{\mathrm{KL}}(q, \nu(|\mathcal{D})) &= \sum_{\boldsymbol{z}} \int (\log q(\boldsymbol{\theta}, \boldsymbol{z}) - \log \ell(\boldsymbol{\theta}) - \log \pi(\boldsymbol{\theta}, \boldsymbol{z}) + \log m(\mathcal{D}))q(\boldsymbol{\theta}, \boldsymbol{z})d\boldsymbol{\theta} && \text{(S3.127)}\\
&= \underbrace{\sum_{\boldsymbol{z}} \int (\log q(\boldsymbol{\theta}, \boldsymbol{z}) - \log \pi(\boldsymbol{\theta}, \boldsymbol{z}))q(\boldsymbol{\theta}, \boldsymbol{z})d\boldsymbol{\theta}}_{\mathrm{d}_{\mathrm{KL}}(q,\pi)}\\
&\quad + \sum_{z} \int (\log \ell(\boldsymbol{\theta}_0) - \log \ell(\boldsymbol{\theta}))q(\boldsymbol{\theta}, \boldsymbol{z})d\boldsymbol{\theta}\\
&\quad + \underbrace{\log m(\mathcal{D}) - \log \ell(\boldsymbol{\theta}_0)}_{\varepsilon_{2n}} && \text{(S3.128)}
\end{aligned}
$$

where $m(\mathcal{D})$ is the marginal distribution of data as in (2.10). Now, taking $\mathcal{A} = \mathbb{H}^{c}_{M_n\epsilon_n} = \{\boldsymbol{\theta} : \mathrm{d}_{\mathrm{H}}(\nu(\boldsymbol{\theta}_0), \nu(\boldsymbol{\theta})) > M_n\epsilon_n\}$ If Lemma 2.2 and 2.3 hold, then using (Jantre et al., 2023, Lemma A.8) we bound the term ε_{1n} for any $M_n \to \infty$.

$$
\varepsilon_{1n} \leq -\frac{nCM_n^2\epsilon_n^2}{\sum_{l=0}^{L} u_l}
$$

If Lemma 2.4 condition 1 hold, then using (Jantre et al., 2023, Lemma A.9) we bound the term ε_{2n} for any $M_n \to \infty$.

$$
\varepsilon_{2n} \leq nM_n \left(\sum_{l=0}^{L} r_l + \xi \right)
$$

If Lemma 2.4 condition 2 hold, then using (Jantre et al., 2023, Lemma A.10) we bound the term ε_{3n} for any $M_n \to \infty$.

$$
\varepsilon_{3n} \leq nM_n \left(\sum_{l=0}^{L} r_l + \xi \right)
$$

Substituting these bounds into the equation (S3.126):

$$\frac{nCM_n^2\epsilon_n^2}{\sum_{l=0}^L u_l} q^\star(\mathbb{H}^{\complement}_{M_n\epsilon_n}) \leq nM_n\left(\sum_{l=0}^L r_l + \xi\right) + nM_n\left(\sum_{l=0}^L r_l + \xi\right) + \log 2 \tag{S3.129}$$

$$\leq nM_n\left(\sum_{l=0}^L r_l + \xi\right) + nM_n\left(\sum_{l=0}^L r_l + \xi\right) + M_n\left(\sum_{l=0}^L r_l + \xi\right) \tag{S3.130}$$

Expanding and simplifying:

$$q^\star(\mathbb{H}^{\complement}_{M_n\epsilon_n}) \leq \frac{3M_n(\sum_{l=0}^L r_l + \xi)\sum_{l=0}^L u_l}{C_1 M_n^2 \epsilon_n^2} \tag{S3.131}$$

Taking $\epsilon_n = \sqrt{\left(\sum_{l=0}^L r_l + \xi\right)\sum_{l=0}^L u_l}$ and limits as $M_n \to \infty$, we can say

$$q^\star(\mathbb{H}^{\complement}_{M_n\epsilon_n}) \xrightarrow{d} 0 \quad \text{as} \quad n \to \infty,$$

which completes the proof. □

S3.6 Posterior consistency: illustrative example

We consider a simple one-dimensional Gaussian location model

$$X_1, \ldots, X_n \overset{\text{iid}}{\sim} \mathcal{N}(\theta, \sigma^2),$$

where the variance σ^2 is known, and the parameter of interest is $\theta \in \mathbb{R}$. We assume a conjugate Gaussian prior

$$\theta \sim \mathcal{N}(\mu_0, \tau^2),$$

which leads to a Gaussian posterior, given by,

$$\theta \mid \mathbf{X} \sim \mathcal{N}(\mu_n, s_n^2),$$

where

$$\mu_n = \frac{\frac{n}{\sigma^2}\bar{X}_n + \frac{1}{\tau^2}\mu_0}{\frac{n}{\sigma^2} + \frac{1}{\tau^2}}, \quad s_n^2 = \left(\frac{n}{\sigma^2} + \frac{1}{\tau^2}\right)^{-1}$$

Assume that the observed data $\mathbf{X} = X_1, \ldots, X_n \overset{\text{iid}}{\sim} \mathcal{N}(\theta_0, \sigma^2)$, with true parameter θ_0. $\bar{X}_n$ represents the sample mean. As the sample size increases, the posterior variance

$$s_n^2 = \frac{\sigma^2}{n + \frac{\sigma^2}{\tau^2}} \approx \frac{\sigma^2}{n}, \quad n \to \infty,$$

decreases at the rate $\mathcal{O}(1/n)$, reflecting the fact that posterior uncertainty about θ shrinks at the same rate as the sampling variability of the sample mean. Moreover, by the law of large numbers, the sample mean satisfies

$$\bar{X}_n \xrightarrow{\text{a.s.}} \theta_0,$$

where θ_0 denotes the *true* data-generating parameter of the model. Substituting this limit into the posterior mean expression above, we find that as $n \to \infty$, the contribution of the prior term $\frac{1}{\tau^2}\mu_0$ becomes negligible relative to the data term $\frac{n}{\sigma^2}\bar{X}_n$, implying that $\mu_n \to \theta_0$ almost surely. Hence, the posterior mean converges to the true data-generating parameter θ_0.

Consequently, the posterior distribution $\nu_n(\theta) = \mathcal{N}(\mu_n, s_n^2)$ concentrates around the point mass δ_{θ_0}, demonstrating *posterior consistency* with contraction rate $\mathcal{O}(n^{-1/2})$. This asymptotic behavior aligns with the Bernstein–von Mises theorem, which asserts that under standard regularity conditions, the posterior distribution becomes asymptotically normal around the true parameter θ_0, centered at the maximum likelihood estimator with variance proportional to $1/n$.

Hellinger distance and neighborhoods For two Gaussian distributions with the same variance σ^2, the squared Hellinger distance is

$$\mathrm{d}_{\mathrm{H}}^2\big(\mathcal{N}(\mu_1, \sigma^2), \mathcal{N}(\mu_2, \sigma^2)\big) = 1 - \exp\left[-\frac{(\mu_1 - \mu_2)^2}{8\sigma^2}\right]. \tag{S3.132}$$

Inverting (S3.132), the parameter separation $\Delta(\varepsilon)$ corresponding to a Hellinger radius ε satisfies

$$|\mu_1 - \mu_2| \le \Delta(\varepsilon) = \sqrt{-8\sigma^2 \log(1 - \varepsilon^2)}. \tag{S3.133}$$

Hence, the Hellinger neighborhood $\mathbb{H}_\varepsilon(\delta_{\theta_0})$ around δ_{θ_0} corresponds to the interval

$$\{\, \theta : |\theta - \theta_0| \le \Delta(\varepsilon) \,\}.$$

For small ε, $\Delta(\varepsilon) \approx 2\sqrt{2}\,\sigma\,\varepsilon$. In Bayesian consistency analysis, we study how the posterior probability of this neighborhood approaches one as n increases:

$$\nu\big(H(\theta, \theta_0) \le \varepsilon_n \mid \mathbf{X}\big) \;\to\; 1, \qquad n \to \infty.$$

When $\varepsilon_n = M/\sqrt{n}$, this corresponds to the parametric contraction rate $O(n^{-1/2})$.

Simulation study We simulate the normal model with $\theta_0 = 0$, $\sigma = 1$. We assume a weak prior $\mathcal{N}(0, 10^6)$. For a single dataset at different n, the posterior $\mathcal{N}(\mu_n, s_n^2)$ is plotted (normal curves) together with the interval $\theta_0 \pm \Delta(\varepsilon_n)$ (as shaded regions, each color represents a choice of n) corresponding to $\varepsilon_n = M/\sqrt{n}$ with $M = 1$ as shown in Figure S1. As n grows, the posterior variance s_n^2 decreases at the rate $1/n$, and almost all the posterior mass lies within the Hellinger neighborhood.

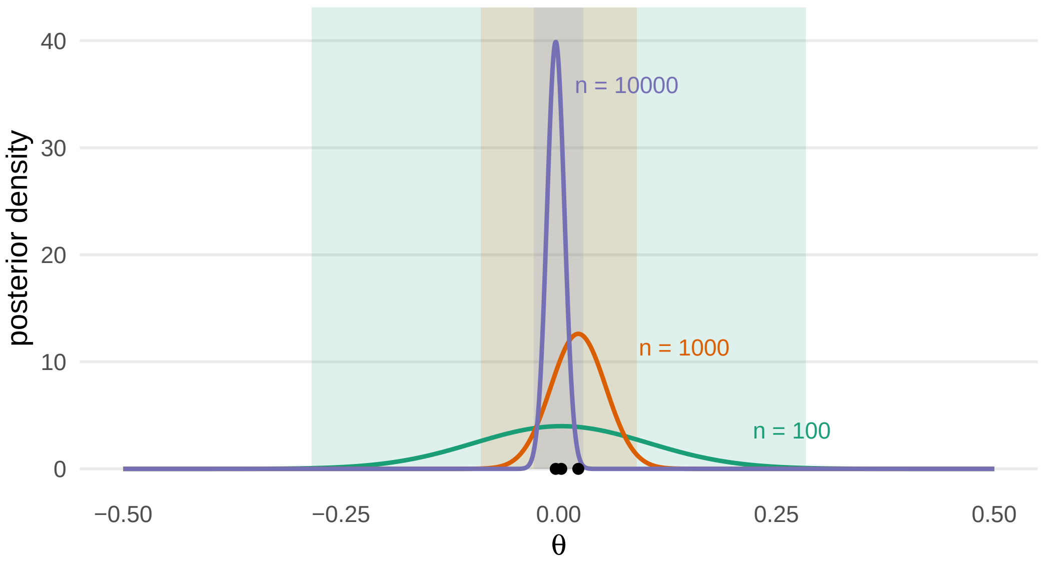

Figure S1: Posterior density with Hellinger neighborhood for the normal-normal model. The colored normal curves shows the posterior density $\nu(\theta \mid \mathbf{X})$ for $n \in \{100, 1000, 10000\}$, and the shaded regions represent the corresponding Hellinger neighborhoods $\mathbb{H}_{\varepsilon_n}(\delta_{\theta_0})$ with $\varepsilon_n = M/\sqrt{n}$ ($M = 1$). The black dots represent the posterior means. The area under the curve within a shaded region represents the posterior mass inside the corresponding neighborhood. As n increases, the posterior concentrates within this region, demonstrating posterior consistency.

S4 Additional experimental details

When implementing the wsBNN model, there are two approaches to consider to manage the first-layer weights.

wsBNN In the first approach, weights for each input feature share a common inclusion probability but are sampled independently from the same spike-and-slab prior with shared parameters, allowing more flexibility in modeling feature-level variability.

wsBNN-tw In the second approach, all weights that come from the same input feature node are tied together and sampled once from a shared spike-and-slab prior. This method reduces the overall parameter space, making it easier to explore the posterior distribution.

In the main paper, we adopted the first approach (shared inclusion probability), which provided improved flexibility and slightly better predictive performance in synthetic data experiments. We note that both formulations yield comparable results in terms of feature selection consistency. See, for example, Table S2, for more details. However, for the real datasets, we used wsBNN-tw because the focus was on feature selection analysis, as highlighted in this supplement.

S4.1 Simulation studies for classification and regression

Classification performance and feature selection consistency We consider a two-class classification problem, motivated by (Friedman, 1991), where data samples $\boldsymbol{x}_i \in \mathbb{R}^d, y_i \in \{0, 1\}, i =$

Table S2: Classification performance of different models on the simulated data, via (3.1), mentioned in the main paper.

	Error analysis†				Feature selection*					
Model	Accuracy	Precision	Recall	F1	FC_1	FC_2	FC_3	FC_1	FC_5	FNR
wsBNN	.95±.06	.95±.04	.95±.06	.95±.06	7	9	10	10	10	0.08±.10
wsBNN-tw	.85±.01	.85±.01	.85±.01	.85±.01	9	3	10	10	10	0.16±.12
STG	.75±.09	.71±.21	.75±.09	.69±.14	0	0	10	10	10	0.40±.00
HC	.64±.01	.46±.11	.64±.01	.50±.02	0	0	0	0	0	1.00±.00
LASSO	.85±.01	.85±.01	.85±.01	.85±.01	8	6	10	10	10	0.12±.09

† Reported as weighted Precision, Recall, and F1-score (on test sets), averages over ten runs.

* Feature selection Consistency (FC) and False Negative Rate (FNR), computed over ten runs.

$1, 2, \ldots$ are generated by the hierarchical model 3.1, as described in the main paper.

The average test accuracy, weighted precision, weighted recall, and weighted F1-score were calculated on simulated data, as summarized in Table S2. Each row in the table represents a different model, while each column denotes a specific metric. The LASSO model was implemented using the `scikit-learn` library (Pedregosa et al., 2011). LASSO performed competitively, slightly trailing behind wsBNN-tw but outperforming both STG and HC. The term wsBNN-tw refers to the wsBNN implementation with tied weights, meaning all weights from an input feature node are shared and sampled once from a spike-and-slab prior. In this context, wsBNN demonstrated better predictive performance than wsBNN-tw, although it showed comparable feature selection performance or a slight advantage over wsBNN-tw.

Regression performance and feature selection consistency To generate the regression dataset (cf. Friedman (1991); Breiman (1996)), the input features $\boldsymbol{x} \in [0,1]^d$ are sampled from a uniform distribution over $[0, 1]$. The target variable $y \in \mathbb{R}$ is calculated using a non-linear function $f(\boldsymbol{x})$ as defined below:

$$f(\boldsymbol{x}) = 10 * \sin(\pi x_1 x_2) + 20(x_3 - 0.5)^2 + 10x_4 + 5x_5 + \xi, \quad \xi \sim N(0, 1). \tag{S4.1}$$

The non-linear function $f(\boldsymbol{x})$ (S4.1) models interactions among input features while promoting sparsity. Specifically, these functions involve only a subset of the input d-dimensional features: $\boldsymbol{x}[:, 0], \boldsymbol{x}[:, 1], \boldsymbol{x}[:, 2], \boldsymbol{x}[:, 3], \boldsymbol{x}[:, 4]$. This selective inclusion ensures that the output y is influenced solely by these five features, making the generated dataset particularly suitable for evaluating feature selection methods. We generate data points (x_i, y_i), $i = 1, 2, \ldots, 5000$. We randomly split the samples into 80% for training and 20% for testing. The training set is further split into a 9:1 ratio, allocating 90% of the samples for training and 10% for validation.

Similar to the classification setting, for each regression model, the evaluation involves selecting the top 10 features, after which the model is retrained using only these features. For regression models, we assess performance using Mean Squared Error (MSE), Bias, and Variance, estimated using the `MLxtend` library[1]. Table S3 shows the average values of the mean squared error, Bias^2,

[1] `https://rasbt.github.io/mlxtend/`

Table S3: Regression performance of different models on the simulated dataset, via (S4.1)

	Error analysis†			Feature selection⋆					
Model	MSE	Bias^2	Variance	FC_1	FC_2	FC_3	FC_1	FC_5	FNR
wsBNN-tw	4.7855±0.54	4.4717±0.22	0.3138±0.33	9	10	1	10	9	.22±.14
STG	23.8605±0.83	23.8535±0.83	0.0070±0.00	10	10	5	10	10	.10±.10
HC	23.2439±1.04	23.2367±1.04	0.0073±0.00	10	10	4	10	10	.12±.09
LASSO	8.5714±0.22	8.5620±0.22	0.0094±0.00	10	10	0	10	0	.40±.00
RF	78.2443±2.74	68.9004±2.63	9.3438±0.34	10	10	10	10	10	.00±.00
GB	23.1800±1.13	18.8587±1.10	4.3213±0.13	10	10	10	10	10	.00±.00

† Evaluated using weighted Mean Squared Error (MSE), Bias^2, and Variance (on test sets), averages over ten runs.

⋆ Feature selection Consistency (FC) and False Negative Rate (FNR), computed over ten runs.

and Variance on the test dataset for different models. wsBNN has the lowest MSE compared to all other models suggesting it generalizes better than other models in predicting the regression outputs. Bias^2 is nearly equal to MSE for all models suggesting that all error comes from Bias^2 rather than variance. wsBNN has a higher variance compared to other models. STG, HC, and LASSO exhibit much higher MSE indicating weaker performance in their predictions. These models also have extremely low variance indicating rigid models that are not adapting to different training sets. wsBNN balances Bias^2 and variance better, allowing for more flexibility in learning.

Similar to the classification studies, we show the consistency of feature selection for the regression data in Table S3. wsBNN shows strong consistency in feature selection, selecting f_1, f_2, f_4, and f_5 in most of the runs while achieving the best MSE. LASSO consistently selects the same features across multiple runs, achieving perfect scores for f_1, f_2, and f_4 while failing to select f_3 and f_5. Both STG and HC models show similar performance in feature selection, consistently selecting features f_1, f_2, f_4, and f_5, but with a diminished selection of f_3. Figure S2 visualizes weight distributions for the regression dataset, showing consistent trends where relevant features exhibit higher positive weight values. The wsBNN model implementation used in this section is based on tied weights in the first layer, i.e., wsBNN-tw.

S4.2 Sensitivity analysis of Inclusion Probability λ_0

The hyperparameter $\lambda_0 \in (0, 1)$ in the spike-and-slab prior controls the prior belief about the inclusion of features. A smaller λ_0 favors sparsity by assigning more probability mass to the spike component (zero weights), whereas a larger λ_0 allows more features to be included.

In this section, we conduct a sensitivity analysis of λ_0 to examine its influence on the number of features selected in the first layer and the model's performance on the test set. We trained our wsBNN model on the simulated dataset for classification by varying $\lambda_0 \in \{.1, .3, .5, .7, .9\}$ and keeping temperature γ as constant at .7. All other hyperparameters, including learning rate, network architecture, and training settings, were kept fixed as mentioned in Section S4.8. For each λ_0, we ran the model for a few runs and averaged the results as shown in Table S4. We visualize the effect of λ_0 on precision@k and test accuracy in Figure S3. We observe that when we increase

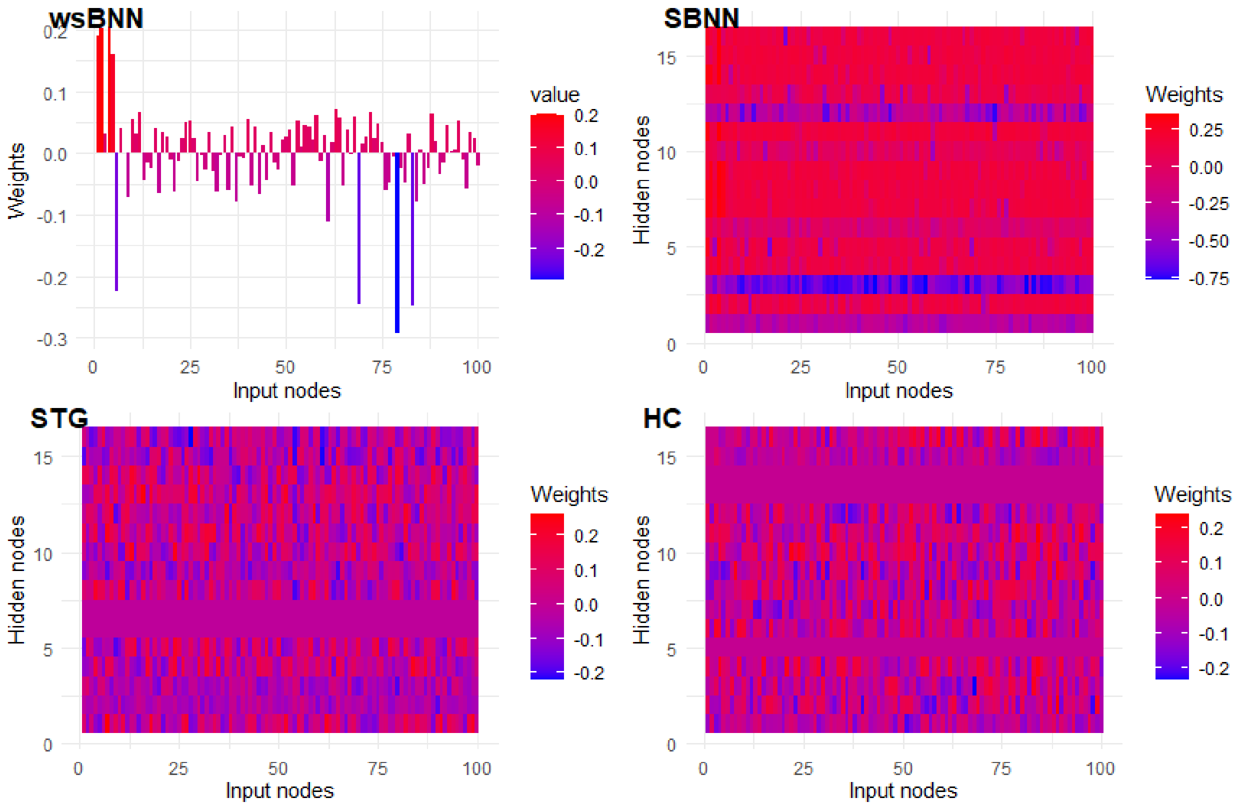

Figure S2: Normalized weights between the input and first hidden layer for four models: wsBNN-tw (top left), SBNN (top right), STG (bottom left), and HC (bottom right) on simulated data S2. The x-axis shows input nodes, while the y-axis indicates weight values (wsBNN) or the number of hidden nodes (SBNN, STG, HC). wsBNN highlights important features (first five) with distinct weights, whereas SBNN, STG, and HC exhibit noisier and less interpretable weight patterns, making it harder to identify key features.

Table S4: Sensitivity analysis of the wsBNN model hyperparameter inclusion probability λ_0

λ_0	**Precision@k**	**Test Accuracy**	**Weighted F1-score**
.1	.30	.6220±.000	.4770±.000
.3	.30	.6500±.000	.5121±.000
.5	.36	.6610±.000	.5261±.000
.7	.36	.6660±.000	.5325±.000
.9	.36	.9520±.004	.9561±.004

λ_0, test accuracy is better at $\lambda_0 = 0.9$ and precision@k (with $k = 10$) increases with increasing λ_0, suggesting the model is selecting more relevant features.

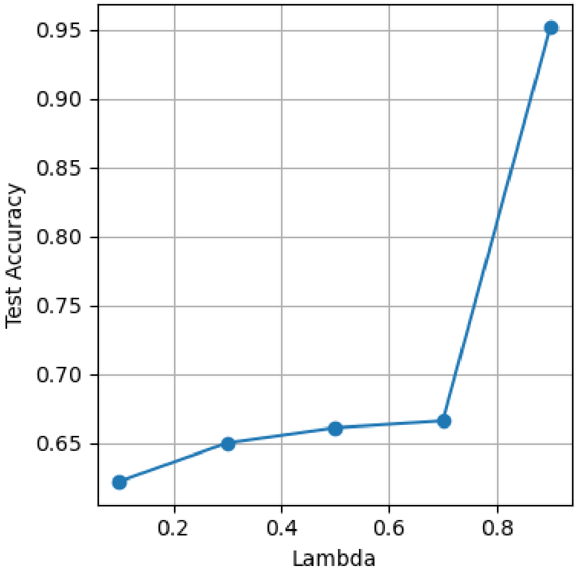

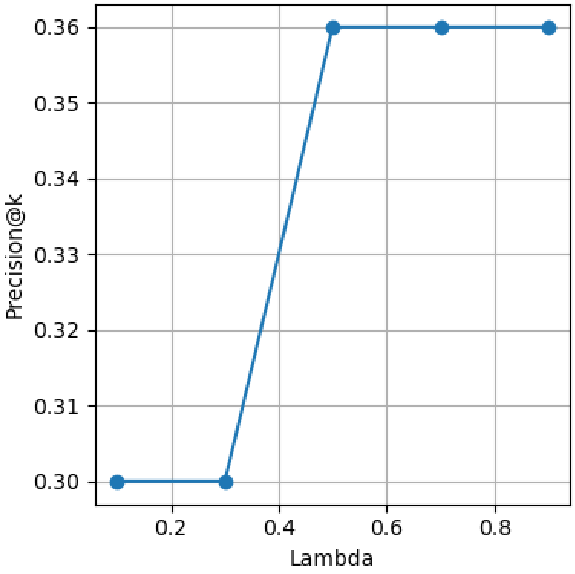

Figure S3: Sensitivity analysis of the wsBNN model with respect to the hyperparameter inclusion probability λ. The figure shows the effect of varying $\lambda \in (0, 1)$ on test accuracy with higher λ values yielding improved performance (left) and the corresponding effect on precision@k (with $k = 10$), where higher λ values approach the higher precision of 0.36 (right).

S4.3 Sensitivity analysis of Temperature γ

In this section, we examine the sensitivity of the parameter gamma γ, which regulates the degree of approximation. We vary the value of gamma $\gamma \in \{0.1, 0.3, 0.5, 0.7, 0.9\}$ after setting inclusion probability $\lambda_0 = .9$ and observe its impact on feature selection. All other hyperparameters, including learning rate, network architecture, and training settings, were kept constant as described in Section S4.8. For each gamma γ, we trained the model over a few runs and averaged the results,

which are presented in Table S5. Furthermore, we visualize the effects of gamma on test accuracy (left) and precision@k with $k = 10$ (right) in Figure S4.

Table S5: Sensitivity analysis of the wsBNN model hyperparameter temperature γ

γ	**Precision@k**	**Test Accuracy**	**Weighted F1-score**
.1	.40	.9310±.033	.9304±.034
.3	.36	.9360±.043	.9356±.043
.5	.30	.9247±.036	.9240±.036
.7	.50	.9297±.036	.9294±.036
.9	.43	.9487±.014	.9484±.014

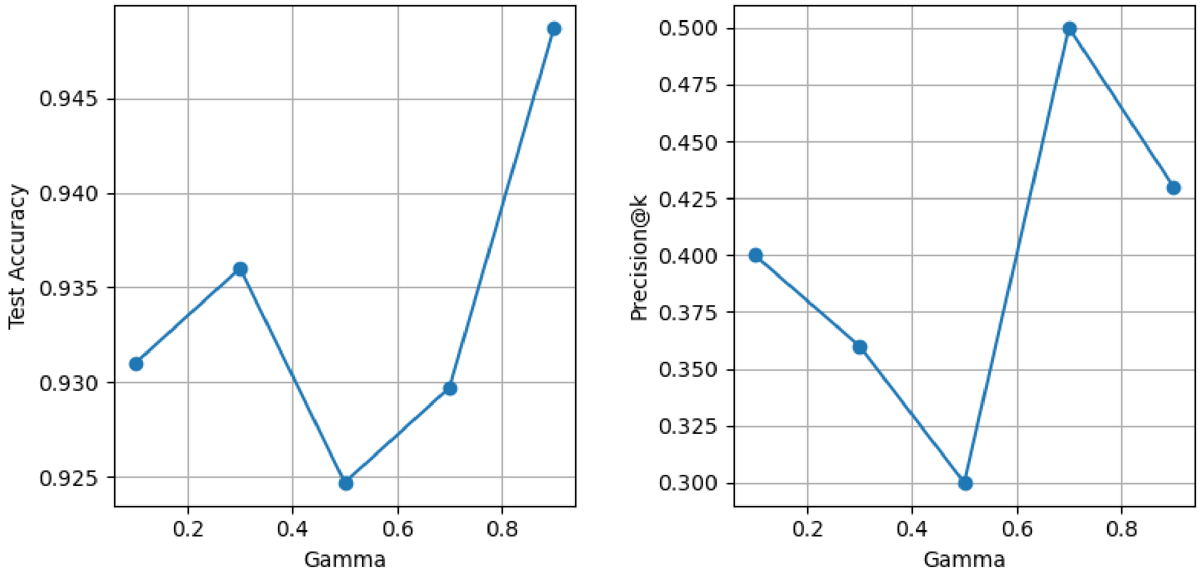

Figure S4: Sensitivity analysis of the wsBNN model regarding the hyperparameter temperature γ. The figure illustrates the impact of γ on test accuracy and precision@k (with $k = 10$, maximum precision 0.5).

S4.4 Training loss progression

Figure S5 shows the training loss evolution across epochs for four models, displaying how efficiently each model minimizes its objective function over time on the simulated classification data, via 3.1, as described in the main paper. A lower, smoother final loss indicates better convergence and training stability. The proposed wsBNN model shows efficient and stable convergence, benefiting from parameter sharing in Bayesian neural networks. On the other hand, sBNN struggles

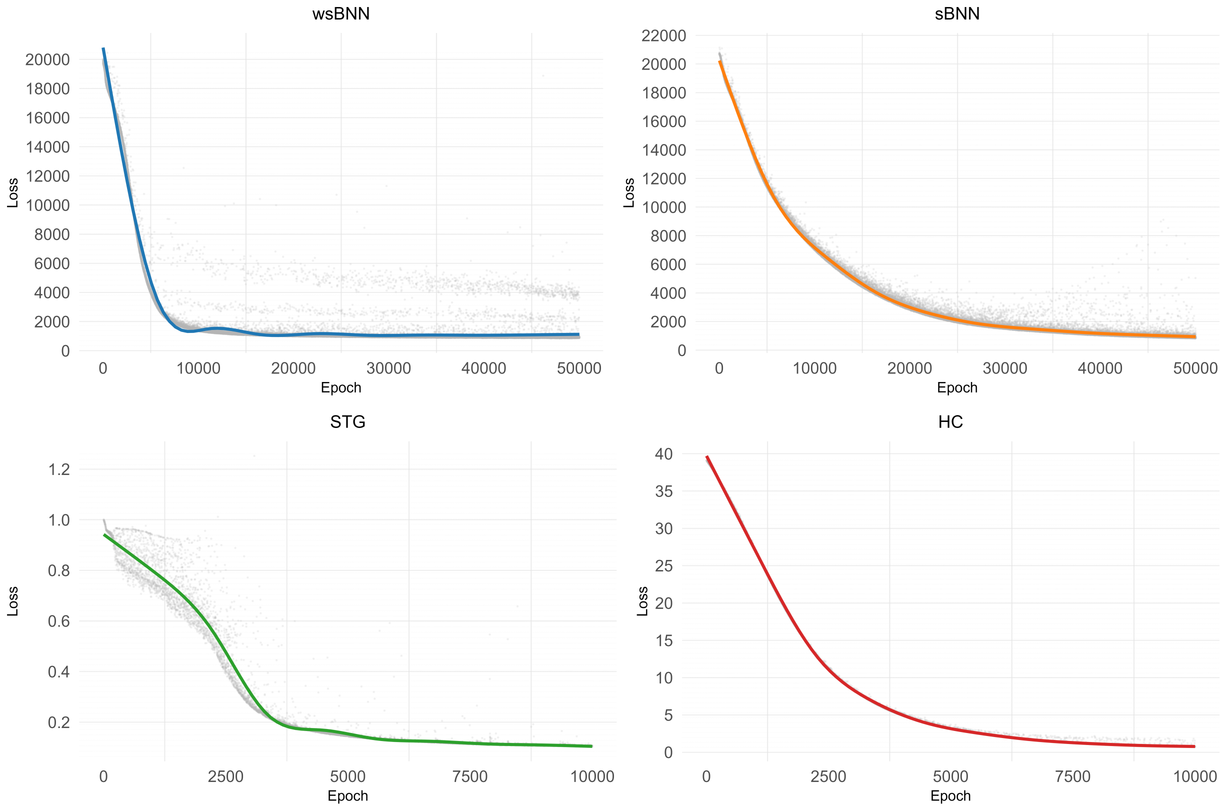

Figure S5: Training loss comparison of wsBNN, sBNN, STG, and HC models until convergence.

with instability due to independent parameter updates and the need for a larger parameter space exploration. The wsBNN architecture addresses these issues by grouping weights from the first layer's features and applying a smooth prior to later layers, reducing model complexity and the number of learnable parameters. Both STG and HC models achieve stable training and convergence; however, STG has mild oscillations during the early training phase, whereas HC maintains a relatively smoother loss trajectory. Nonetheless, these frequentist approaches lack the flexibility and variance control provided by Bayesian counterparts sBNN and wsBNN.

S4.5 Scalability study of wsBNN on real datasets

To assess the scalability of the proposed wsBNN model[2], we compared its training time and peak memory usage against two competing deep learning models—STG and HC—across multiple real datasets (Table S6).

Overall, wsBNN requires moderately higher computational resources than STG and HC. Specifically, wsBNN's training times range from approximately 0.42 seconds (BRCA dataset) to 0.54 seconds (Gisette dataset), which is consistently higher than STG and HC due to the additional computations associated with Bayesian inference and weight-sharing mechanisms. Similarly, wsBNN exhibits higher peak memory usage, with values ranging from around 1.02 MB to 1.32 MB,

[2]The experiments are based on wsBNN-tw, tied weights in the first layer

Table S6: Execution time and memory usage of three competing deep learning models for the four real datasets.

Dataset	Model	Training time (s)	Peak Memory (MB)
BRCA	wsBNN-tw	0.4167	1043.70
	STG	0.0139	556.61
	HC	0.0171	555.64
PCMAC	wsBNN-tw	0.4741	1027.17
	STG	0.0193	625.41
	HC	0.0219	625.36
Gisette	wsBNN-tw	0.5437	1320.33
	STG	0.1112	1065.04
	HC	0.1202	1065.88
Basehock	wsBNN-tw	0.4633	1053.05
	STG	0.0457	672.78
	HC	0.0293	676.91

compared to 0.55–1.06 MB for the competing methods.

While wsBNN incurs a modest computational overhead, these costs are justified by its improved feature selection interpretability and robustness. Importantly, the scalability results indicate that wsBNN remains computationally feasible even for datasets with thousands of features, making it suitable for large-scale feature selection problems in high-dimensional domains such as genomics.

S4.6 Stability analysis using Kuncheva Index

We employ the Kuncheva (2007) Index (KI) to quantify the stability of feature selection across multiple runs of the same model. Let S_a and S_b be the sets of selected features from two different runs, each of size k, and let $r = |S_a \cap S_b|$ be the size of their intersection. Given n as the total number of features, the Kuncheva Index is computed as:

$$\text{KI} = \frac{r \cdot n - k^2}{k \cdot (n - k)}$$

For the simulated datasets, where the ground-truth relevant features are known, we restricted our analysis to the intersection between the model-selected features and the true feature set. Considering d as the total number of true features, the Kuncheva Index is computed as:

$$\text{KI} = \frac{r \cdot d - k^2}{k \cdot (d - k)}$$

The range of KI is $[-1, 1]$, where KI $= 1$ indicates perfect stability (identical selections across runs), KI $= 0$ indicates stability equal to random chance, and KI < 0 indicates less agreement than expected by chance.

Table S7: Kuncheva index (KI) calculated for various models and datasets

models	wsBNN-tw	SBNN	STG	HC	LASSO	RF	GB
Friedman	0.93	0.66	1.00	0.00	0.74	0.55	0.35
BRCA	0.30	0.50	0.08	0.79	0.28	0.53	0.22
PCMAC	0.51	0.50	0.00	1.00	0.71	0.86	0.74
Gisette	0.48	0.76	0.00	0.52	0.59	0.57	0.75
Basehock	0.51	0.64	0.00	0.46	0.79	0.82	0.82

We calculated the KI for all $\binom{n}{2}$ run pairs and reported the average as the stability score. For simulated dataset, we took $n_{\text{runs}} = 10$, $k = 10$, and $d = 5$. Only features belonging to the known ground-truth relevant set were considered in the stability computation. For other datasets, we took $k = 30$. Table S7 presents the stability analysis of selected features across multiple datasets using KI, which measures the consistency of feature selection under repeated runs. Higher KI values indicate more stable feature selection. wsBNN achieves consistently high stability for synthetic data, such as Friedman (0.93), and competitive stability for real-world datasets, notably outperforming STG and matching or approaching the performance of strong baselines like LASSO, RF, and GB in several cases. SBNN shows good stability in datasets like Gisette (0.76) and Basehock (0.64), but is generally less consistent than wsBNN on synthetic data. STG, while achieving perfect stability (1.0) for Friedman and PCMAC, shows zero stability in other datasets, highlighting a lack of robustness. HC demonstrates extremes—either perfect stability (1.0) or very low values (0.0–0.52)—indicating dataset-dependent performance. Upon close inspection, we observe that HC tends to select features in a random manner, often choosing sequential blocks of features rather than selecting truly meaningful and informative features from across the dataset. Overall, wsBNN provides a balanced and reliable feature selection stability profile across diverse datasets, without the instability seen in some competing methods.

S4.7 TCGA-BRCA data

Figure S6 shows the heatmap of the BRCA gene expression dataset, where rows represent gene expression values and columns represent samples. The heatmap is generated using the R package `ComplexHeatmap` for visualizing high-dimensional gene data with clustering and annotations. In addition, we applied the R package `mclust` for model-based clustering of samples, generating the *Cluster-1*, *Cluster-2*, and *Cluster-3* labels shown in the heatmap. The model was fitted using a Gaussian finite mixture model with an EEI covariance structure (equal volume and shape, axis-aligned clusters) and three components, as determined by the `Mclust` function.

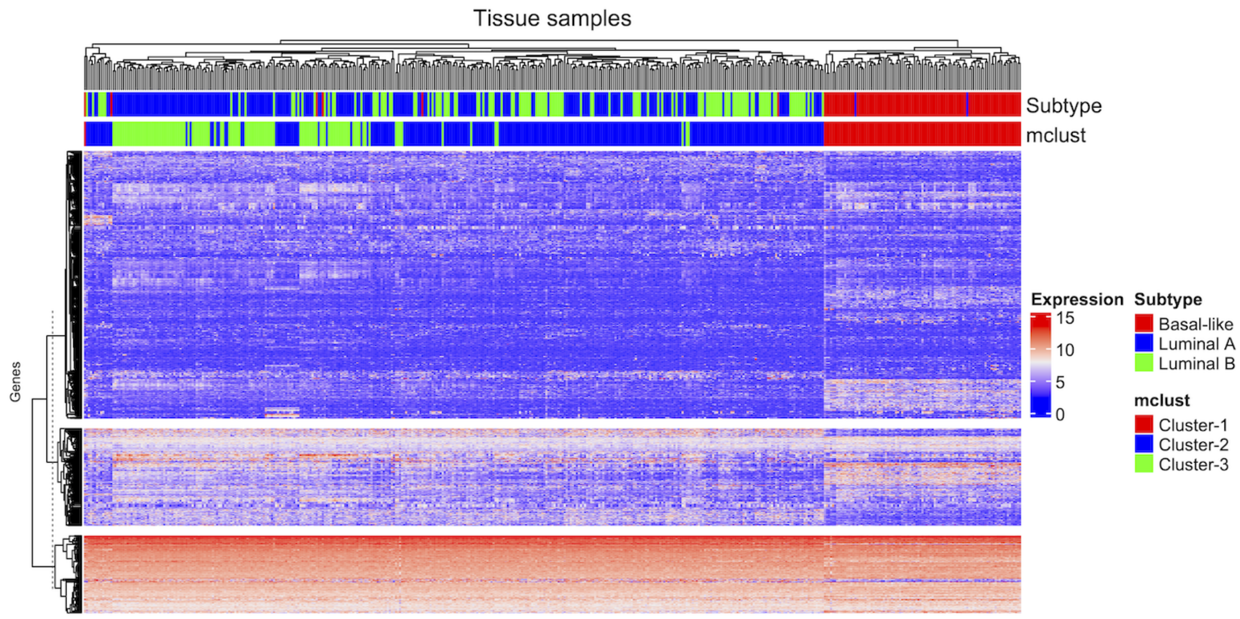

Figure S6: Heatmap of BRCA gene expression data. Tissue samples (columns) correspond to three molecular subtypes: Basal-like (101), Luminal A (233), and Luminal B (127). Hierarchical clustering of both genes and samples was applied to improve visualization, and clustering results obtained using the R package `mclust` were overlaid for comparative analysis.

S4.8 Hyperparameter tuning for deep learning models

For a fair comparison, we kept the same network architecture across all models, e.g., the number of hidden layers and the number of nodes in each layer. We adjusted the number of training epochs according to the convergence dynamics of each model. Specifically, the Bayesian models (wsBNN and sBNN) were trained for 50,000 epochs, while the frequentist models (STG and HC) were trained for 10,000 epochs. In STG and HC, we set a constant $\sigma = 0.5$ and a regularization parameter $\lambda \in \{0.05, 0.5, 0.5\}$ for different datasets. In wsBNN and sBNN, we use inclusion probability $\lambda_0 = .9$ and temperature $\gamma = .7$. We used `ReLU` activation function and `Adam` optimizer for all neural network-based models for comparison. Batch size and learning rate vary in different datasets as reported in Table S8.

Table S8: Hyperparameters selected for different models

Dataset	STG / HC		wsBNN / sBNN	
	Batch size	Learning rate	Batch size	Learning rate
BRCA	294	0.01	92	0.001
PCMAC	450	0.50	163	0.001
Gisette	1000	0.20	163	0.001
Basehock	50	0.50	163	0.001

Table S9: Performance comparison of competing models on real-world datasets

Model	wsBNN-tw	sBNN	STG	HC	LASSO	RF	GB
Accuracy							
BRCA	.7032±.07	.5742±.18	.7559±.13	.7634±.13	.8000±.04	.8882±.03	.8850±.03
PCMAC	.8419±.08	.9079±.02	.4938±.02	.4938±.02	.9044±.01	.9252±.01	.8879±.01
Gisette	.9613±.01	.9753±.01	.4910±.01	.5019±.01	.9791±.00	.9708±.00	.9702±.00
Basehock	.9185±.05	.9496±.05	.4842±.01	.4922±.02	.9659±.01	.9757±.01	.9514±.01
Weighted Precision							
BRCA	.5532±.13	.3894±.20	.7258±.23	.7247±.23	.8625±.04	.8906±.03	.8896±.04
PCMAC	.8781±.05	.9131±.01	.2441±.02	.2441±.02	.9061±.01	.9271±.01	.9000±.01
Gisette	.9615±.01	.9755±.01	.2411±.01	.2521±.01	.9791±.00	.9711±.00	.9703±.00
Basehock	.9310±.04	.9545±.03	.2346±.01	.2427±.02	.9670±.01	.9761±.01	.9541±.01
Weighted Recall							
BRCA	.7032±.07	.5742±.18	.7559±.13	.7634±.13	.8602±.04	.8882±.03	.8850±.03
PCMAC	.8419±.08	.9079±.02	.4938±.02	.4938±.02	.9044±.01	.9252±.01	.8879±.01
Gisette	.9613±.01	.9753±.01	.4910±.01	.5019±.01	.9791±.00	.9708±.00	.9702±.00
Basehock	.9185±.05	.9496±.05	.4842±.01	.4922±.02	.9659±.01	.9757±.01	.9514±.01
Weighted F1-score							
BRCA	.6094±.11	.4472±.20	.7212±.19	.7299±.19	.8598±.04	.8868±.03	.8835±.04
PCMAC	.8339±.09	.9077±.02	.3267±.02	.3267±.02	.9043±.01	.9251±.01	.8870±.01
Gisette	.9613±.01	.9753±.01	.3234±.01	.3356±.02	.9791±.00	.9708±.00	.9702±.00
Basehock	.9171±.06	.9490±.05	.3160±.01	.3249±.02	.9659±.01	.9757±.01	.9514±.01

S4.9 Classification performance of various feature selection models

We now evaluate the performance of different feature selection models based on test F1-score, accuracy, precision, and recall across multiple real-world datasets, including gene expression data. In this study, each model selects its own features, and a new classifier is then trained and tested using only those selected features. This setup is therefore distinct from the approach used in the main paper (Mishra et al., 2026), where retraining and testing were performed using a single common model, RF, to assess the quality of selected features. The results, summarized in Table S9, represent mean values over ten independent runs for each metric. Across datasets, wsBNN consistently achieved comparable accuracy, outperforming sBNN on the BRCA dataset and obtaining comparable or superior results to other state-of-the-art methods on Gisette and Basehock. The key difference between wsBNN and sBNN is that wsBNN groups weights from the same input feature in the first layer and applies smooth priors in later layers, reducing complexity and variance while maintaining interpretability. In contrast, sBNN updates all weights independently, leading to potential cancellation effects when averaging positive and negative weights—an issue reflected in its lower precision on the BRCA dataset.

Both Bayesian models outperformed frequentist baselines (STG and HC) by achieving a better balance across metrics and effectively managing uncertainty. While LASSO showed high accuracy

on some datasets, its linear structure limits interpretability and suitability for genomic applications. wsBNN achieved a strong balance of accuracy, precision, and recall, highlighting the advantages of structured weight sharing and sparsity-inducing priors in enhancing robustness and interpretability in high-dimensional feature selection. Consistent with the findings reported in the main paper (Mishra et al., 2026), ensemble-based models such as Random Forest (RF) and Gradient Boosting (GB) outperformed all other approaches in terms of overall predictive performance.

S4.10 Feature selection consistency on real data, for LASSO, STG, wsBNN

We evaluated the consistency of feature selection on real datasets using the linear model LASSO and compared its performance with the nonlinear models STG and wsBNN. Figure S7 compares the Bayesian-model wsBNN with the classical linear method LASSO, and a non-linear frequentist neural network STG. For each model, the top 30 features were identified across 10 independent runs on four real datasets. LASSO consistently selected a similar set of features across multiple runs, indicating stable feature selection. For all datasets except BRCA, LASSO and wsBNN showed a high degree of overlap in the selected features, while a few features (genes) were uniquely identified by LASSO. A detailed comparison of wsBNN, STG, and RF is provided in the main paper (Mishra et al., 2026).

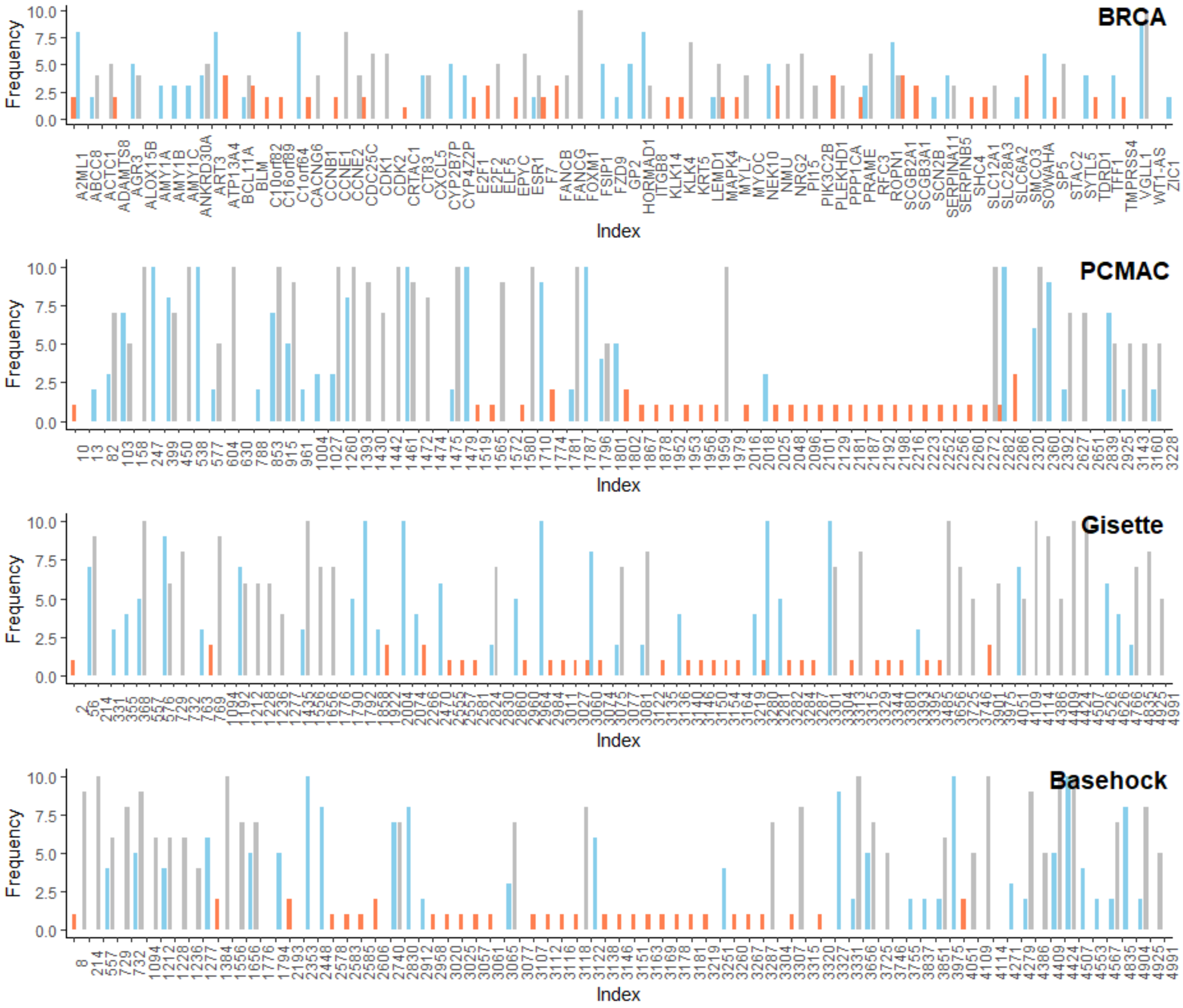

Figure S7: Frequencies of the top 30 features selected across 10 independent runs of four real-world datasets: BRCA, PCMAC, Gisette, and Basehock. The x-axis shows feature indices (names), and the y-axis shows frequencies. Blue bars: wsBNN-tw, orange bars: STG, gray bars: LASSO.

References

Akanksha Mishra, Wei Xia, and Clint Pazhayidam George. A Weight-sharing Bayesian Neural Network for Consistent Feature Selection with Applications in Cancer Gene Expression Data. Technical report, School of Mathematics and Computer Science, Indian Institute of Technology Goa, 2026.

Ulf Grenander. Abstract inference. *(No Title)*, 1981.

Stuart Geman and Chii-Ruey Hwang. Nonparametric maximum likelihood estimation by the method of sieves. *The annals of Statistics*, pages 401–414, 1982.

David Pollard. Bracketing methods in statistics and econometrics. In *Nonparametric and semiparametric methods in econometrics and statistics: Proceedings of the Fifth International Symposium in Eco*, pages 337–355. Cambridge University Press, 1991.

Johannes Schmidt-Hieber. Nonparametric regression using deep neural networks with ReLU activation function. *The Annals of Statistics*, 48(4):1875 – 1897, 2020.

Sanket Jantre, Shrijita Bhattacharya, and Tapabrata Maiti. Layer adaptive node selection in bayesian neural networks: Statistical guarantees and implementation details. *Neural Networks*, 167:309–330, 2023.

Jincheng Bai, Qifan Song, and Guang Cheng. Efficient variational inference for sparse deep learning with theoretical guarantee. *Advances in Neural Information Processing Systems*, 33:466–476, 2020.

Subhashis Ghosal and Aad van der Vaart. Convergence rates of posterior distributions for noniid observations. *The Annals of Statistics*, 35(1):192–223, 2007. ISSN 00905364.

Badr-Eddine Chérief-Abdellatif and Pierre Alquier. Consistency of variational bayes inference for estimation and model selection in mixtures. 2018.

Shrijita Bhattacharya and Tapabrata Maiti. Statistical foundation of variational bayes neural networks. *Neural Networks*, 137:151–173, 2021.

Jerome H Friedman. Multivariate adaptive regression splines. *The annals of statistics*, 19(1):1–67, 1991.

F. Pedregosa, G. Varoquaux, A. Gramfort, V. Michel, B. Thirion, O. Grisel, M. Blondel, P. Prettenhofer, R. Weiss, V. Dubourg, J. Vanderplas, A. Passos, D. Cournapeau, M. Brucher, M. Perrot, and E. Duchesnay. Scikit-learn: Machine learning in Python. *Journal of Machine Learning Research*, 12:2825–2830, 2011.

Leo Breiman. Bagging predictors. *Machine learning*, 24:123–140, 1996.

Ludmila I Kuncheva. A stability index for feature selection. In *Artificial intelligence and applications*, pages 421–427, 2007.
